# Ground State Energy Fluctuations of Pinned Elastic Manifolds

**DOI:** 10.1007/s10955-026-03628-9

**Published:** 2026-05-29

**Authors:** Yan V. Fyodorov, Bertrand Lacroix-A-Chez-Toine, Pierre Le Doussal

**Affiliations:** 1 Department of Mathematics, King’s College London, London, WC2R 2LS United Kingdom; 2 Laboratoire de Physique de l’Ecole Normale Supérieure, ENS, Université PSL, CNRS, Sorbonne Université, Université de Paris, 75005 Paris, France

## Abstract

We describe the atypical fluctuations of the ground state energy of the random elastic manifold, a disordered model defined on a lattice of linear size *L* with internal dimension $0\le d<4$ embedded in a medium of dimension $N\gg 1$. The ground-state energy results from a competition between confinement, elasticity and disorder. We obtain an exact description of the large deviation rate function with speed $NL^d$ and its different phases, corresponding to different patterns of replica symmetry breaking (RSB). Our results show that the ground-state energy satisfies a central limit theorem and we obtain an explicit expression for the rescaled variance. In the (massless) limit of zero confinement, this variance vanishes for short-range disorder and the ground-state energy displays super-concentration. From our results on the large deviation function, we characterise explicitly the left tail of the distribution of the typical fluctuations of the ground state energy. It displays an exponential tail for a one step RSB pattern while for a full RSB pattern it decays super-exponentially with a non trivial exponent $\xi$ that we compute explicitly.

## Introduction

The optimal configuration adopted at equilibrium by many physical systems results from the competition between elasticity, promoting flat, homogeneous configurations, and disorder, promoting rugged, heterogeneous configurations [1]. The elastic manifold is a versatile and well-studied model to describe such physical systems. Let us define it on a *d*-dimensional lattice with linear size *L* and a lattice spacing *a*. At each point $\textbf{x}$ of the lattice we define a *N*-dimensional field $\textbf{u}(\textbf{x})$. The Hamiltonian of the elastic manifold then reads

1 $$\begin{aligned} \mathcal{H}[\textbf{u}]=\frac{1}{2}\sum _{\textbf{x},\textbf{y}}  \textbf{u}(\textbf{x})(\mu \delta _{\textbf{x},\textbf{y}}-t \Delta _{\textbf{x},\textbf{y}})\textbf{u}(\textbf{y})+\sum _{\textbf{x}}\, V(\textbf{u}(\textbf{x}),\textbf{x})\;, \end{aligned}$$

where $\delta _{\textbf{x},\textbf{y}}=1$ if $\textbf{x}=\textbf{y}$ and zero otherwise, $\Delta$ is the Laplacian matrix, $\mu \ge 0$ is the stiffness of the harmonic potential (that we will call mass in the following) and $t\ge 0$ is the interaction strength. In the following we use units such that $t=1$. The random field $V(\textbf{u}(\textbf{x}),\textbf{x})$ is Gaussian with zero average and covariance

2 $$\begin{aligned} \left\langle V(\textbf{u}_1,\textbf{x}_1)V(\textbf{u}_2,\textbf{x}_2) \right\rangle =\delta _{\textbf{x}_1,\textbf{x}_2}\,N\,f\left( \frac{(\textbf{u}_1-\textbf{u}_2)^2}{2N}\right) \;. \end{aligned}$$

The first term in the Hamiltonian (1) is simply optimised by setting $\textbf{u}(\textbf{x})=\textbf{0}$ while the configuration optimising the Hamiltonian in presence of the second (disordered) term is non-trivial. It will be convenient to introduce the expression of the Hamiltonian (1) in Fourier space where the first term is diagonal

3 $$\begin{aligned} \mathcal{H}[\textbf{u}]=\frac{1}{2}\sum _{\textbf{k}}\nu (\textbf{k})\textbf{u}(\textbf{k})\textbf{u}(-\textbf{k})+\sum _{\textbf{x}}\, V(\textbf{u}(\textbf{x}),\textbf{x}) \end{aligned}$$

where $k_j= 2 \pi n_j/L$ and the elastic modulus $\nu (\textbf{k})$ takes the form

4 $$\begin{aligned} \nu (\textbf{k})\simeq \textbf{k}^2 + \mu \;,\;\;|\textbf{k}|a \ll 1\;. \end{aligned}$$

To ensure translation invariance explicitly, we take in the following periodic boundary conditions. In the following, we denote $\int _{\textbf{k}}=L^{-d} \sum _{\textbf{k}}$ for finite *L* and *a*. As we will be interested in the scaling of various observables with *L* in the following, we will keep it finite while the limit $a\rightarrow 0$ (which does not yield any divergence in dimensions $0<d<4$) will often be considered explicitly. In the continuum limit $L\rightarrow \infty$ and $a\rightarrow 0$ one can replace $\int _{\textbf{k}}\rightarrow \int \frac{ d \textbf{k} }{(2 \pi )^d}$. The equilibrium static properties of this Hamiltonian are studied at finite inverse temperature $\beta$ by computing the quenched free energy

5 $$\begin{aligned} \left\langle F_{N,L}(\beta ) \right\rangle =-\frac{1}{\beta }\left\langle \ln Z_{N,L}(\beta ) \right\rangle \;,\;\;Z_{N,L}(\beta )=\int \mathcal{D}  \textbf{u}(\textbf{x})e^{-\beta \mathcal{H}[\textbf{u}]}\;. \end{aligned}$$

As the inverse temperature diverges, the latter converges towards the average ground-state energy

6 $$\begin{aligned} \left\langle \mathcal{E}_{N,L} \right\rangle = \left\langle \min _{\textbf{u}} \mathcal{H}[\textbf{u}] \right\rangle =\lim _{\beta \rightarrow \infty } F_{N,L}(\beta )\;. \end{aligned}$$

Two main methods are available to study this model in the low temperature regime: (i) the functional renormalisation group, valid for any *N* but perturbative around $d=4$ [2], and (ii) the replica saddle point method in the limit $N \rightarrow +\infty$ which is valid for any *d* [3]. The matching between these quite different approaches at large but finite *N* was investigated in [4–7]. The focus of the present article is to compute the fluctuations of the ground state energy in the limit $N\rightarrow \infty$ using approach (ii). It is worth noting that the variance of the *difference* in ground state energy induced by a small perturbing field was computed in that limit in [7], with applications to avalanches and shocks in the closely related problem of Burger’s turbulence [8–10]. However, to our knowledge, neither the variance of the ground state energy, nor its large deviation function, have been explicitly computed previously.

In that limit, the rescaled free-energy is a self-averaging random variables, i.e.

7 $$\begin{aligned} \lim _{N\rightarrow \infty }\mathcal{F}_{N,L}(\beta )=\lim _{N\rightarrow \infty }\frac{F_{N,L}(\beta )}{NL^d}\underset{\mathrm{a.s.}}{=}\lim _{N\rightarrow \infty }\left\langle \mathcal{F}_{N,L}(\beta ) \right\rangle =\mathcal{F}_{d}(\beta ,L)\;, \end{aligned}$$

and the limiting quenched free energy $\mathcal{F}_{d}(\beta ,L)$ has been computed explicitly in the physics literature using replica and saddle-point techniques, notably by Mezard and Parisi [3] (see also [11–13]). These results have recently been re-examined using rigorous methods in a series of papers by Ben Arous and Kivimae [14, 15]. The limiting free energy is then found to be represented via the "Parisi formula" as the solution of an optimisation problem over a space of probability measures, with the optimal probability measure describing the overlap distribution between different configurations optimising the free-energy. The self-averaging property holds similarly at zero temperature and a Parisi formula can similarly be derived for the limiting ground-state energy, which concentrates around the typical value $e_{L,\mathrm typ}(\mu )=\lim _{N\rightarrow \infty }\mathcal{E}_{N,L}/(NL^d)=\lim _{\beta \rightarrow \infty }\mathcal{F}_{d}(\beta ,L)$.

A particularly interesting outcome of the paper by Mezard and Parisi [3] is the computation of the scaling behaviour of the variance of the free energy with the size *L* of the lattice. Using dimensional analysis and renormalisation group arguments, they showed that in the massless limit $\mu \rightarrow 0$ a simple relation exists between the growth exponent of the free energy variance with the lattice size in the limit $L\rightarrow \infty$,

8 $$\begin{aligned} \textrm{Var}(F_{N,L}(\beta ))\sim L^{2\chi }\;,\;\;\textrm{where}\;\;\chi =2\zeta +d-2\;, \end{aligned}$$

and the growth exponent of the transverse fluctuations of the manifold, i.e.

9 $$\begin{aligned} C_{N,L}(\textbf{l},\beta )=\left\langle \frac{1}{Z_{N,L}(\beta )}\int \mathcal{D}  \textbf{u}(\textbf{x})e^{-\beta \mathcal{H}[\textbf{u}]}\left( \textbf{u}(\textbf{x}+\textbf{l})-\textbf{u}(\textbf{x})\right) ^2 \right\rangle \sim |\textbf{l}|^{2\zeta }\;. \end{aligned}$$

They also computed explicitly the value of $\zeta$ in dimensions $0<d<4$ for a family of correlation functions *f*(*q*) as defined in Eq. (2). The variance can be shown to diverge in the continuum limit $L/a\rightarrow \infty$ in any dimension $d\ge 4$. Interestingly, it was found that the exponent $\chi > 0$ is positive when the free-energy is given by its full replica-symmetry broken (FRSB) expression while it is negative or zero otherwise. For negative exponents, the variance of the free-energy $F_{N,L}(\beta )$ decreases with the lattice size *L* and the free-energy is thus a self-averaging variable in the limit $L\rightarrow \infty$. The rescaled free-energy $\mathcal{F}_{N,L}(\beta )$ is instead self-averaging in the limit $L\rightarrow \infty$ whenever the weaker criterion $\zeta < 1$ is satisfied.

The goal of this paper is to study the fluctuations of the rescaled ground-state energy defined as

10 $$\begin{aligned} e_{\min }=\frac{\mathcal{E}_{N,L}}{NL^d}=\frac{1}{NL^d}\min _\mathbf{u(\textbf{x})}\mathcal{H}[\textbf{u}]=\lim _{\beta \rightarrow \infty }\mathcal{F}_{N,L}(\beta )\;. \end{aligned}$$

In particular, we will obtain an explicit expression for the rescaled variance in the large *N* limit, defined as

11 $$\begin{aligned} \mathcal{V}_L(\mu )=\lim _{N\rightarrow \infty }NL^d \textrm{Var}(e_{\min })=\lim _{N\rightarrow \infty }\frac{\textrm{Var}(\mathcal{E}_{N,L})}{N L^d}\;. \end{aligned}$$

This rescaled variance depends non-trivially on the length *L* and the mass $\mu$ and, in particular, in the massless limit $\mu \rightarrow 0$ the variance $\mathcal{V}_L(0)\sim L^{2\tilde{\chi }}$ is predicted to grow with the exponent $\tilde{\chi }=2\zeta +(d-4)/2$ [3]. Thus, our result not only reproduces the scaling exponent obtained by Mezard and Parisi [3] in an alternative way (since $\tilde{\chi }=\chi -d/2$), but provides an exact, quantitative expression much beyond. In addition, we characterise the atypical fluctuations of $e_\textrm{min}$ by computing the large deviation function with speed $NL^d$ in the limit $N\rightarrow \infty$,

12 $$\begin{aligned} \Lambda _L(e,\mu )=-\lim _{N\rightarrow \infty }\frac{1}{NL^d}\ln \left\langle \delta (e-e_{\min }) \right\rangle \;. \end{aligned}$$

We will also sometimes be interested in the continuum limit and define the following large deviation function in that limit

13 $$\begin{aligned} \mathcal{L}(\epsilon ,\mu )=\lim _{L\rightarrow \infty }\Lambda _L(e_{L,\mathrm typ}(\mu )+\epsilon ,\mu )\;. \end{aligned}$$

In the following, we need to distinguish between two classes of disordered potential (see [16] for a detailed physical discussion and [17] for rigorous results):

(i) Short range disorder, such that the covariance function *f*(*q*) satisfies $f(q)=\int _0^{\infty } dk\,e^{-q k^2}\,\tilde{f}(k)$ with positive coefficients $\tilde{f}(k)\ge 0$ for any $k\ge 0$. In this case the fluctuations of the ground state energy $e_{\min }$ can be investigated directly.

(ii) Long range disorder, such that the disordered potential is a random process with stationary increments, such that $f(q) - f(0) =\int _0^{\infty } dk\,e^{-q k^2}\,\tilde{f}(k)$ with positive $\tilde{f}(k)\ge 0$ for any $k\ge 0$. In this case, the fluctuations of the energy difference with respect to the flat reference configuration need to be considered

14 $$\begin{aligned} \Delta e_{\min }= \frac{1}{NL^d}\min _{\textbf{u}(\textbf{x})} ( \mathcal{H}[\textbf{u}] - \mathcal{H}[\textbf{0}] ) = \frac{1}{NL^d}\left( \mathcal{E}_{N,L} - \sum _\textbf{x} V(\textbf{0},\textbf{x})\right) \;. \end{aligned}$$

The random variable $\Delta e_{\min }$ is by definition a negative random variable and the probability that $\Delta e_{\min }>0$ is identically zero. As $V(\textbf{0},\textbf{x})$ is a zero average Gaussian random variable, it is simple to check that $e_{\min }$ and $\Delta e_{\min }$ have the same average value. Finally, let us mention that this average value $\left\langle e_{\min } \right\rangle =\left\langle \Delta e_{\min } \right\rangle$ depends on the microscopic details, in particular the lattice sizes *a* and *L* and it can be shown to diverge for any internal dimension $d\ge 2$ in the continuum limit $L/a\rightarrow \infty$.

As has been shown both in the physics [3] and mathematics [14, 15] literature, the typical value of the ground-state energy is determined by the replica symmetric expression for $\mu$ above the so-called Larkin mass $\mu _{c}$. The latter is defined as the unique solution $\mu$ of $f''(0)I_2(\mu =\mu _{c})=1$, with $I_2(\mu )$ defined by taking $p=2$ in the expression

15 $$\begin{aligned} I_p(x)=\int _\textbf{k}\frac{1}{\left( x-\mu +\nu (\textbf{k})\right) ^p}\;. \end{aligned}$$

This Larkin mass has been recently shown not only to determine the onset of the replica-symmetry breaking transition but also the associated topology trivialisation transition from an energy function (Hamiltonian) with a single minimum (for $\mu \ge \mu _c$) to a Hamiltonian displaying a number of minima growing exponentially with the dimension *N* (for $\mu <\mu _c$) [18, 19]. We will show in the following that the Larkin mass also plays an important role in the description of higher-order cumulants of $e_{\min }$.

In the following, we will focus our attention on the cumulants of $e_{\min }$ and its large deviation function $\Lambda _L(e,\mu )$ for short-range disorder and provide some results for the cumulants of $\Delta e_{\min }$ for long-range disorder.

The paper is structured as follows. In section 2 we summarise the main results of the paper, providing a Parisi formula for the cumulant generating function of $e_{\min }$ as well as explicit expressions for the rescaled variance $\mathcal{V}_L(\mu )$ and the large deviation function $\Lambda _L(e,\mu )$. In section 3 we provide the detailed derivation of the expression of the cumulant generating function as an optimisation problem. In section 4 we analyse the expression of the cumulant generating function and large deviation function at different levels of replica-symmetry breaking. In section 5 we obtain explicit expressions for the large deviation function in the continuous limit in a few specific internal dimensions *d* and for some choice of covariance function *f*(*q*). Finally in section 6 we conclude and provide some ideas for future directions. Some technical details are relegated to the appendices. In appendix A we analyse the average of $e_{\min }$. In appendix B we compute the annealed complexity of minima at fixed energy which provides a bound for the large deviation function.

## Summary of the Main Results

Unless specified otherwise, the results below apply for the ground-state energy $e_{\min }$ for a short-range disorder such that the correlation function decays at infinite distance $\lim _{q\rightarrow \infty }f(q)=0$. The results for the ground-state energy difference $\Delta e_{\min }$ for a long-range disorder are summarised in the last subsection.

### Typical Fluctuations

For any value $\mu >0$, we obtain an explicit expression for the rescaled variance

16 $$\begin{aligned} \mathcal{V}_{L}(\mu )=&\lim _{N \rightarrow \infty }\frac{\textrm{Var}(\mathcal{E}_{N,L})}{N L^d}=f\left( Q_\textrm{typ}\right) -\frac{I_2(\mu ) f'\left( Q_\textrm{typ}\right) ^2}{2}\;, \end{aligned}$$

where $Q_\textrm{typ}$ is the difference between largest and smallest overlap values, i.e. the range of the support of "Parisi function" describing the typical distribution of overlaps. In the RS phase, the distribution of overlaps is supported on a single atom and $Q_\textrm{typ}=0$ while in the replica symmetry broken phases (both 1RSB and FRSB) $Q_\textrm{typ}>0$. The expression for $Q_\textrm{typ}$ is RS, hence vanishes, for any mass larger than the Larkin mass $\mu >\mu _c$ and RSB for $\mu <\mu _c$ [3, 14]. The criterion for selecting the pattern of replica symmetry breaking is not simple but still explicit (see Eq. (A32) and the following paragraph).

In the FRSB phase, $Q_\textrm{typ}$ is given by a simple expression

17 $$\begin{aligned} Q_\textrm{typ}={f''}^{-1}\left( \frac{1}{I_2(\mu )}\right) \;, \end{aligned}$$

where we remind that $I_p(\mu )$ is defined from Eq. (15) and ${f''}^{-1}$ is the inverse function of $f''$. In the 1RSB phase, $Q_\textrm{typ}$ is the positive solution of Eqs. (95-96).

This rescaled variance is strictly positive for any $\mu >0$ and as a direct consequence, the ground-state energy satisfies the central limit theorem

18 $$\begin{aligned} \lim _{NL^d\rightarrow \infty }\textrm{Prob}\left[ \frac{\mathcal{E}_{N,L}-\left\langle \mathcal{E}_{N,L} \right\rangle }{\sqrt{\textrm{Var}(\mathcal{E}_{N,L})}}\le x\right] =\frac{1}{2}\left( 1+\textrm{erf}\left( \frac{x}{\sqrt{2}}\right) \right) \;. \end{aligned}$$

The energy fluctuations are thus of order $\sim (NL^d)^{1/2}$.

As we show below, the value of $Q_\textrm{typ}$ diverges in the limit $\mu \rightarrow 0$ in any internal dimension $0<d<4$ which implies that the rescaled variance in Eq. (16) vanishes. This in turn implies that the ground-state energy density "super-concentrates", similarly to what is observed for spherical spin-models at zero magnetic field [20], and the central limit theorem breaks down in that limit. Our results indicate that in the case $\mu \rightarrow 0$ the limiting distribution takes a different form for different patterns of replica symmetry breaking. Based on results derived below, we expect:

1. For a 1RSB pattern, we conjecture the limiting distribution
  19 $$\begin{aligned} \lim _{NL^d\rightarrow \infty }\textrm{Prob}\left[ \mathcal{E}_{N,L}-\left\langle \mathcal{E}_{N,L} \right\rangle \le x\right] =\mathcal{P}_{d,\mathrm 1RSB}(x)\;. \end{aligned}$$
  The energy fluctuations are thus of order *O*(1). This limiting distribution displays an exponential left tail
  20 $$\begin{aligned} 0<\Sigma _{\min }'(e_\textrm{typ},0)=\lim _{x\rightarrow -\infty }\frac{1}{x}\ln \mathcal{P}_{d,\mathrm 1RSB}(x)<\infty \;, \end{aligned}$$
  where $\Sigma _{\min }(e,\mu )$ is the annealed complexity of minima at energy density *e* and for mass $\mu$ and $e_\textrm{typ}=\lim _{NL^d\rightarrow \infty }\mathcal{E}_{N,L}/(NL^d)$.
2. In contrast, when the solution to Parisi optimization problem is controlled by FRSB pattern, we conjecture the following limiting distribution of typical fluctuations
  21 $$\begin{aligned} \lim _{NL^d\rightarrow \infty }\textrm{Prob}\left[ \frac{\mathcal{E}_{N,L}-\left\langle \mathcal{E}_{N,L} \right\rangle }{(NL^d)^{\chi /d}}\le x\right] =\mathcal{P}_{d,\mathrm FRSB}(x)\;, \end{aligned}$$
  where $0\le \chi <2$ is the exponent of the variance computed by Mezard and Parisi (which depends on the asymptotic behaviour of the covariance function *f*(*q*)). The energy fluctuations in this case are thus of order $\sim (NL^d)^{\chi /d}$ with $0<\chi /d<1/2$. Additionally, we conjecture that the limiting distribution displays the left tail behaviour
  22 $$\begin{aligned} -\ln \mathcal{P}_{d,\mathrm FRSB}(x)\approx r_d(-x)^{\xi }\;,\;\;x\rightarrow -\infty \;,\;\;\textrm{with}\;\;\xi =\frac{d}{d-\chi }\;, \end{aligned}$$
  and $r_d$ is a constant that can be computed explicitly. The exponent $\xi$ corresponds to that of the large deviation function in the vicinity of the typical value, i.e.
  23 $$\begin{aligned} \mathcal{L}(\epsilon )=-\lim _{NL^d\rightarrow \infty }\frac{1}{NL^d}\ln \textrm{Prob}\left[ \frac{\mathcal{E}_{N,L}-\left\langle \mathcal{E}_{N,L} \right\rangle }{NL^d}\le \epsilon \right] =\lim _{L\rightarrow \infty }\Lambda _L(e_{L,\mathrm typ}(0)+\epsilon ,0)\;, \end{aligned}$$
  where the function $\mathcal{L}(\epsilon )\approx r_d(-\epsilon )^{\xi }$ as $\epsilon \rightarrow 0_-$. This exponent can be recovered by a simple matching argument between the tail of the regime of typical fluctuations described by Eq. (22) and the large deviation regime in Eq. (23), expressing both the rescaled variables *x* and $\epsilon$ in terms of $\mathcal{E}_{N,L}-\left\langle \mathcal{E}_{N,L} \right\rangle$ and matching the powers of $NL^d$ in both equations.

The behaviour described above is reminiscent of what has been observed for the fluctuations of the spherical spin model in [21]. It was shown there that under the FRSB ansatz the ground-state fluctuations display a non-trivial exponent while such an exponent is always equal to unity within the 1RSB ansatz. Using an explicit approach to derive the large deviation function $\mathcal{L}(\epsilon )$ in the following sections, we determine independently the FRSB value of $\xi$ in Eq. (22) and of the exponential tail (i.e. an exponent $\xi =1$) in the 1RSB pattern in Eq. (20). Let us now provide more details on our results for the cumulant generating function and large deviation function of the ground-state energy.

### General Expression for the Cumulant Generating Function

The scaled cumulant generating function (CGF) of the ground-state energy $e_{\min }$ is expressed as

24 $$\begin{aligned} \varphi _L(s,\mu )=\lim _{N\rightarrow \infty }\frac{1}{N L^d}\ln \left\langle e^{-s N L^d e_{\min }} \right\rangle ={\left\{ \begin{array}{ll} \displaystyle \max _{g(\textbf{k},z),G_0(\textbf{k}),v(\textbf{k}),w_M}\mathcal{S}_{0,L}(\mu )& \;,\;\;s<0\;, \\ 0& \;,\;\;s=0\;, \\ \displaystyle \min _{g(\textbf{k},z),G_0(\textbf{k}),v(\textbf{k}),w_M}\mathcal{S}_{0,L}(\mu )& \;,\;\;s>0\;, \end{array}\right. } \end{aligned}$$

where the functional $\mathcal{S}_{0,L}(\mu )$ reads

25 $$\begin{aligned} \mathcal{S}_{0,L}(\mu )=&-\frac{s}{2}\int _\textbf{k}\nu (\textbf{k})\left[ g(\textbf{k},s)+G_0(\textbf{k})\right] \end{aligned}$$

26 $$\begin{aligned}&+\frac{s}{2}\left[ -f'(0)\int _\textbf{k}v(\textbf{k})+w_M\,f\left( 0\right) -\int _{s}^{w_M}dz\,f\left( \int _\textbf{k}g(\textbf{k},z)\right) \right] \nonumber \\&+\frac{1}{2}\int _\textbf{k}\left[ -\frac{s}{w_M}\ln v(\textbf{k})+\ln \left( \lambda (\textbf{k},s)+s G_0(\textbf{k})\right) -s\int _{s}^{w_M}\frac{dz}{z^2}\ln \lambda (\textbf{k},z)\right] \;,\nonumber \\ \textrm{with}&\;\;\lambda (\textbf{k},z)=v(\textbf{k})+z\,g(\textbf{k},z)+\int _{z}^{w_M}dw\,g(\textbf{k},w)\;. \end{aligned}$$

Here the (breaking point) parameter $w_M$ satisfies $w_M\ge s$, the functions $v(\textbf{k})\ge 0$ and $G_0(\textbf{k})\ge 0$ are positive and the function $g(\textbf{k},z)$ is a positive non-increasing function defined over the interval $z\in [s,w_M]$. Optimising over $w_M$ yields the explicit boundary condition $g(\textbf{k},z\ge w_M)=0$. The expression above is valid for any pattern of replica-symmetry breaking (replica-symmetric ($w_M=s$), one-step replica symmetry breaking, full replica-symmetry breaking).

**Remark:** For $s\ne 0$, the expression of the cumulant generating function results from the properties of optimal atypical configurations. The values of the different parameters $w_M,v(\textbf{k}),G_0(\textbf{k}),g(\textbf{k},z)$ in the optimisation problem above describe the statistical properties of these optimal atypical configurations (see section 3.4 for a detailed discussion).

As we show in appendix B, using an exact result relying on the Kac-Rice approach, the cumulant generating function is bounded from above as

27 $$\begin{aligned} \varphi _L(s,\mu )\le \frac{s^2 f(0)}{2}+\frac{1}{2}\int _\textbf{k}\ln \left( 1-\frac{s f'(0)}{\nu (\textbf{k})}\right) +\Sigma _{\min }(\mu -s f'(0))\;, \end{aligned}$$

where $\Sigma _{\min }(\mu )$ is the annealed complexity of energy functional minima for the mass parameter $\mu$. For $\mu -s f'(1)>\mu _c$ one finds $\Sigma _{\min }(\mu -s f'(1))=0$ and the term on the right-hand side is shown to coincide with the replica-symmetric (RS) expression of the cumulant generating function, corresponding to $w_M=s$.

The value for the average/typical centered ground state energy can be obtained from the general expression for the CGF above as

28 $$\begin{aligned} e_{L,\mathrm typ}(\mu )=-\partial _s\varphi _L(0,\mu )=\displaystyle \max _{g(\textbf{k},z),G_0(\textbf{k}),v(\textbf{k}),w_M}\mathcal{S}_{L,\mathrm typ}(\mu ) \end{aligned}$$

where the expression of $\mathcal{S}_\textrm{typ}$ is obtained as

29 $$\begin{aligned} \mathcal{S}_{L,\mathrm typ}(\mu )=&-\lim _{s\rightarrow 0}\frac{\mathcal{S}_{0,L}(\mu )}{s}\nonumber \\ =&\frac{1}{2}\int _\textbf{k}\nu (\textbf{k})g(\textbf{k},0)+\frac{1}{2}\left[ f'(0)\int _\textbf{k}v(\textbf{k})-w_M\,f\left( 0\right) +\int _{0}^{w_M}dz\,f\left( \int _\textbf{k}g(\textbf{k},z)\right) \right] \nonumber \\&+\frac{1}{2}\int _\textbf{k}\left[ \frac{1}{w_M}\ln \frac{v(\textbf{k})}{\lambda (\textbf{k},0)}+\int _{0}^{w_M}\frac{dz}{z^2}\ln \frac{\lambda (\textbf{k},z)}{\lambda (\textbf{k},0)}\right] +\frac{1}{2}\int _\textbf{k}G_0(\textbf{k})\left[ \nu (\textbf{k})-\frac{1}{\lambda (\textbf{k},0)}\right] \;. \end{aligned}$$

In the following, we use $g_\textrm{typ}(\textbf{k},z)$ and $w_\textrm{typ}$ as the values of $g(\textbf{k},z)$ and $w_{k}$ optimising Eq. (28). The expression obtained from Eq. (28) corresponds to the limit of zero temperature of the free-energy derived using the replica method in [3] and has recently been obtained rigorously in [15] (although for the latter the equivalence between the formulae is much less obvious).

The expression for the cumulant generating function obtained above allows to derive higher-order cumulants and, in particular, the variance of the rescaled ground state energy. The latter is defined as

30 $$\begin{aligned} \mathcal{V}_{L}(\mu )=&\lim _{N \rightarrow \infty }\frac{\textrm{Var}(\mathcal{E}_{N,L})}{N L^d}=f\left( Q_\textrm{typ}\right) -\frac{I_2(\mu ) f'\left( Q_\textrm{typ}\right) ^2}{2}\;,\;\; \textrm{where}\;\;Q_\textrm{typ}=\int _\textbf{k}g_\textrm{typ}(\textbf{k},0)\;. \end{aligned}$$

The integral $I_2(x)$ is defined in Eq. (15). The expression in Eq. (30) is valid at any level of RSB while the expression of $Q_\textrm{typ}$ depends instead on the level of replica symmetry breaking. In the simplest, replica-symmetric (RS) phase $Q_\textrm{typ}=0$. In the full replica symmetry broken (FRSB) phase it reads instead $Q_\textrm{typ}={f''}^{-1}\left( 1/I_2(\mu )\right)$, i.e. it is expressed from the functional inverse of the second derivative of the covariance function. Finally, in the one-step replica symmetry broken (1RSB) phase it is obtained as the non-zero solution of the following set of self-consistent equations (eliminating the breaking-point parameter $w_\textrm{typ}$ in the following equations)

31 $$\begin{aligned} Q_\textrm{typ}&=\int _\textbf{k}\frac{1}{w_\textrm{typ}}\left( \frac{1}{\nu (\textbf{k})}-\frac{1}{\nu (\textbf{k})+w_\textrm{typ}(f'\left( Q_\textrm{typ}\right) -f'(0))}\right) \;,\end{aligned}$$

32 $$\begin{aligned}&f\left( Q_\textrm{typ}\right) -f(0)-Q_\textrm{typ} f'\left( Q_\textrm{typ}\right) \nonumber \\&=\frac{1}{w_\textrm{typ}^2}\int _\textbf{k}\left[ \frac{w_\textrm{typ}(f'\left( Q_\textrm{typ}\right) -f'(0))}{\nu (\textbf{k})+w_\textrm{typ}(f'\left( Q_\textrm{typ}\right) -f'(0))} -\ln \left( 1+\frac{w_\textrm{typ}(f'\left( Q_\textrm{typ}\right) -f'(0))}{\nu (\textbf{k})}\right) \right] \;. \end{aligned}$$

The variance of the ground state energy then can be computed as a function of the mass $\mu$ and we find that it undergoes a second order transition at the Larkin mass $\mu _{c}$ corresponding to the emergence of replica-symmetry breaking in the typical ground state energy (see Eqs. (157) and (208)).

For short-range correlation functions, using that $f(q)=\int _0^{\infty } dk\,e^{-q k^2}\,\tilde{f}(k),$ and $\tilde{f}(k)\ge 0$ for any $k\ge 0$, one can show explicitly that

33 $$\begin{aligned}&2f''(q)f(q)-f'(q)^2=\int _0^{\infty }\int _0^{\infty }dk_1 dk_2\,e^{-q(k_1^2+k_2^2)}\tilde{f}(k_1)\tilde{f}(k_2)\left( k_1^4+k_2^4-k_1^2 k_2^2\right) \nonumber \\&\ge \int _0^{\infty }\int _0^{\infty } dk_1 dk_2\,e^{-q(k_1^2+k_2^2)} \tilde{f}(k_1)\tilde{f}(k_2)\left( k_1^4+k_2^4-2k_1^2 k_2^2\right) \nonumber \\&=\int _0^{\infty }\int _0^{\infty } dk_1 dk_2 \,e^{-q(k_1^2+k_2^2)}\tilde{f}(k_1)\tilde{f}(k_2)\left( k_1^2-k_2^2\right) ^2\ge 0\;. \end{aligned}$$

The variance is positive in the FRSB phase where $\mathcal{V}_L(\mu )=f(Q_\textrm{typ})-f'(Q_\textrm{typ})^2/(2f''(Q_\textrm{typ}))$ and in the RS phase where $\mathcal{V}_L(\mu )=f(0)-I_2(\mu )f'(0)^2/2$ with $I_2(\mu )\le 1/f''(0)$ as required from the de-Almeida-Thouless criteria on stability of the RS ansatz.

To compare our expression for the variance of the ground state energy with the results obtained by Mezard and Parisi, let us consider an explicit expression for asymptotic behaviour of the short-range correlation function

34 $$\begin{aligned} f(q)\sim -\frac{g}{1-\gamma }q^{1-\gamma }\;,\;\;q\gg 1\;,\;\;\gamma \ge 1\;, \end{aligned}$$

with $g>0$. We will be interested in the limit of large *L* and small $\mu$ where both are finite while the limit $a\rightarrow 0$ can be taken explicitly. Using the behaviour in Eq. (4), we can replace $\nu (\textbf{k})=\mu +\textbf{k}^2$ and introduce a finite circular lower cut-off $(2\pi )/L$ in the $\textbf{k}$ integrals. Let us treat separately the case (i) $d<2$ and $\gamma >\gamma _c=2/(2-d)$ for which the value of $Q_\textrm{typ}$ is obtained from the 1RSB pattern and the case (ii) $d\ge 2$ or $d<2$ and $\gamma < \gamma _c=2/(2-d)$ for which it is obtained from the FRSB pattern.

(i) In the 1RSB pattern, the expression of $Q_\textrm{typ}$ is obtained from Eqs. (31-32). Analysing these equations in the limit $L\rightarrow \infty$ and $\mu \rightarrow 0$ with a fixed dimensionless parameter $x=\mu L^2$, one obtains the scaling form

35 $$\begin{aligned} Q_\textrm{typ}\approx L^{2\zeta _\textrm{1RSB}}\mathcal{Q}_\textrm{1RSB}(\mu L^2)\;,\;\;\zeta _\textrm{1RSB}=\frac{2-d}{2}>0\;, \end{aligned}$$

where the scaling function

36 $$\begin{aligned} \mathcal{Q}_\textrm{1RSB}(x)= &   \frac{1}{w_\textrm{typ}}\frac{2\pi ^{d/2}}{2(2\pi )^2\Gamma (d/2)}\left( -\frac{x}{4\pi ^2}\right) ^{\frac{d-2}{2}}B\left( -\frac{x}{(4\pi )^2};\frac{2-d}{2},0\right) \;,\;\;\nonumber \\  &   w_\textrm{typ}=-\frac{1}{f'(0)}\left( \frac{2d\sin (\frac{d\pi }{2})}{\pi (2-d)}\frac{f(0)}{f'(0)^2}\right) ^{-\frac{2}{4-d}}\;, \end{aligned}$$

and *B*(*x*; *a*, *b*) is the incomplete beta function. This result recovers the exponent derived by Mezard and Parisi [3]. The scaling function behaves asymptotically as

37 $$\begin{aligned} \mathcal{Q}_\textrm{1RSB}(x)\approx {\left\{ \begin{array}{ll}\displaystyle \frac{\pi ^{\frac{d}{2}-2}}{2(2-d)\Gamma \left( \frac{d}{2}\right) w_\textrm{typ}}& \;,\;\;x\ll 1\;, \\ &  \\ \displaystyle \frac{1}{2^d\pi ^{\frac{d}{2}}\Gamma \left( \frac{2-d}{2}\right) w_\textrm{typ}}x^{-\zeta _\textrm{1RSB}}& \;,\;\;x\gg 1\;. \end{array}\right. } \end{aligned}$$

Inserting the value of $Q_\textrm{typ}$ in Eq. (30), the variance of $\mathcal{E}_{N,L}$ can be computed in the scaling limit, yielding

38 $$\begin{aligned}  &   \textrm{Var}(\mathcal{E}_{N,L})\approx NL^d\mathcal{V}_L(\mu )=N L^{2\chi _\textrm{1RSB}}\,V_\textrm{1RSB}(\mu L^2)\;,\;\;\textrm{with} \nonumber \\  &   \chi _\textrm{1RSB}=1-\frac{2-d}{2}\gamma =\frac{\gamma _c-\gamma }{\gamma _c}\le 0\;, \end{aligned}$$

and the scaling function

39 $$\begin{aligned} V_\textrm{1RSB}(x)=\frac{g}{\gamma -1}\left( \mathcal{Q}_\textrm{1RSB}(x)\right) ^{1-\gamma }\;, \end{aligned}$$

so that at large *L* one has $\textrm{Var}(\mathcal{E}_{N,L}) \sim L^{2\chi _\textrm{1RSB}}$ at vanishingly small mass, i.e. $\mu L^2 \rightarrow 0$, while $\textrm{Var}(\mathcal{E}_{N,L}) \sim \mu ^{- \tilde{\chi }} L^d$ with $\tilde{\chi }=(2-d)(1-\gamma )/2$ for fixed $\mu$. Interestingly, both the exponent $\chi$ for the ground state energy variance and $\tilde{\chi }=\chi -d/2$ for the rescaled variance are negative in the 1RSB regime. The result for the exponent $\chi$ in the 1RSB phase is, to the best of our knowledge, new, and it is particularly interesting to note that the relation $\chi =2\zeta +d-2$, which would provide $\chi =0$ is not valid in that phase.

(ii) Under the assumption of validity of FRSB pattern the value of $Q_\textrm{typ}$ satisfies $Q_\textrm{typ}={f''}^{-1}\left( 1/I_2(\mu )\right)$. In the limit $L\rightarrow \infty$ and $\mu \rightarrow 0$ with a fixed dimensionless parameter $x=\mu L^2$, one obtains the scaling form

40 $$\begin{aligned} Q_\textrm{typ}\approx L^{2\zeta _\textrm{FRSB}}\mathcal{Q}_\textrm{FRSB}(\mu L^2)\;,\;\;\zeta _\textrm{FRSB}=\frac{4-d}{2(1+\gamma )}>0\;, \end{aligned}$$

where the scaling function

41 $$\begin{aligned} \mathcal{Q}_\textrm{FRSB}(x)=\left( \frac{1}{g\gamma }\frac{2\pi ^{d/2}}{(2\pi )^4\Gamma (d/2)}\left[ \frac{1}{2(1+\frac{x}{4\pi ^2})}+\frac{d-2}{4}\left( -\frac{x}{4\pi ^2}\right) ^{\frac{d-4}{2}}B\left( -\frac{x}{(4\pi )^2};\frac{4-d}{2},0\right) \right] \right) ^{\frac{1}{1+\gamma }}\;. \end{aligned}$$

This result recovers again the scaling exponent predicted by Mezard and Parisi [3]. The scaling function behaves asymptotically as

42 $$\begin{aligned} \mathcal{Q}_\textrm{FRSB}(x)\approx {\left\{ \begin{array}{ll}\displaystyle \left( \frac{\pi ^{\frac{d}{2}-4}}{8g\gamma (4-d)\Gamma \left( \frac{d}{2}\right) }\right) ^{\frac{1}{1+\gamma }}& \;,\;\;x\ll 1\;, \\ &  \\ \displaystyle \left( -\frac{1}{g\gamma 2^d\pi ^{\frac{d}{2}-1}\Gamma \left( \frac{d-2}{2}\right) \sin \left( \frac{\pi d}{2}\right) }\right) ^{\frac{1}{1+\gamma }}x^{-\zeta _\textrm{FRSB}}& \;,\;\;x\gg 1\;, \end{array}\right. } \end{aligned}$$

where $B_d=-2^{d}\pi ^{\frac{d}{2}-1}\Gamma \left( \frac{d}{2}-1\right) \sin \left( \frac{d\pi }{2}\right)$ appearing in the second line of the equation above takes the finite value $B_2=4\pi$ in dimension $d=2$. Inserting this scaling form in Eq. (30), one can compute the scaling form for the variance as

43 $$\begin{aligned}  &   \textrm{Var}(\mathcal{E}_{N,L})\approx NL^d\mathcal{V}_L(\mu )=N L^{2\chi _\textrm{FRSB}}\,V_\textrm{FRSB}(\mu L^2), \nonumber \\  &   \chi _\textrm{FRSB}=2\zeta _\textrm{FRSB}+d-2=\frac{2+\gamma (d-2)}{1+\gamma }\ge 0\;, \end{aligned}$$

where the expression of the scaling function $V_\textrm{FRSB}(x)$ reads

44 $$\begin{aligned} V_\textrm{FRSB}(x)=\frac{g(1+\gamma )}{2\gamma (\gamma -1)}\left( \mathcal{Q}_\textrm{FRSB}(x)\right) ^{1-\gamma }\;,\;\;1<\gamma \le \gamma _c\;, \end{aligned}$$

so that at large *L* one has $\textrm{Var}(\mathcal{E}_{N,L}) \sim L^{2\chi _\textrm{FRSB}}$ at vanishingly small mass, i.e. $\mu L^2 \rightarrow 0$, while $\textrm{Var}(\mathcal{E}_{N,L}) \sim \mu ^{- \tilde{\chi }} L^d$ with $\tilde{\chi }=(4-d)(1-\gamma )/2/(1+\gamma )$ for fixed $\mu$. The exponent for the variance $\chi$ is positive in the FRSB regime while the exponent for the rescaled variance $\tilde{\chi }=\chi -d/2=2(1-\gamma )\zeta _\textrm{FRSB}$ is negative.

**Fig. 1:**
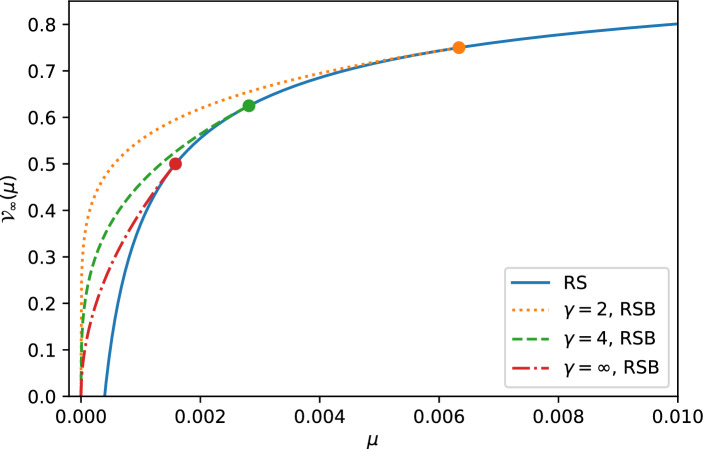
Plot of the rescaled variance $\mathcal{V}_{\infty }(\mu )$ defined in Eq. (30) as a function of the mass $\mu$ for the elastic manifold with internal dimension $d=3$, in the continuous limit $L\rightarrow \infty$ where $\nu (\textbf{k})=\mu +\textbf{k}^2$ and for a short-range covariance of the disorder of the form $f_\gamma (q)=(1+q/(\gamma -1))^{1-\gamma }$ with $\gamma >1$ for $\gamma =2,4,\infty$. Below the RSB transition, i.e. for $\mu <\mu _c$ (with $\mu _c$ indicated by a dot), the variance is given by the FRSB expression (orange dotted, green dashed and red dashed dotted curves for $\gamma =2,4,\infty$ respectively) while it is given by the RS expression (solid blue curve) above the second order transition $\mu >\mu _c$

The exponent in Eq. (43) is in full agreement with the results of Mezard and Parisi [3] using an approach relying on dimensional analysis and renormalisation group. Our approach relies instead on an explicit computation of the ground state energy variance and allows us to provide not only the value for the scaling exponent, but also a quantitative prediction for the associated scaling function. The criterion for the variance increasing with the system size, $\chi \ge 0$, is equivalent to the condition of validity of the FRSB ansatz (either $d \ge 2$ or $d<2$ and $\gamma < \gamma _c=2/(2-d)$). Furthermore, using our results, one can compute the exact expression for the variance for any given correlation function *f*(*q*). In Fig. 1, we plot the rescaled variance as a function of $\mu$ in dimension $d=3$, with elasticity term $\nu (\textbf{k})=\mu +\textbf{k}^2$ in the continuous limit $L\rightarrow \infty$ and $a\rightarrow 0$ and for various short-range correlation functions.

Our results show that for any value $\mu >0$, the rescaled variance $\mathcal{V}_L(\mu )$ in Eq. (30) is positive. Interestingly however as $\mu \rightarrow 0$ the value of $Q_\textrm{typ}$ diverges in any internal dimension $0<d<4$ and our result for the rescaled variance in Eq. (30) indicates that the variance vanishes in that limit (see also Eq. (44) and Fig. 1). If the value of $\chi$ is given by its FRSB expression, the exponent $\xi$ of the large deviation function reads

45 $$\begin{aligned} \xi _\textrm{FRSB}=\frac{d}{d-\chi _\textrm{FRSB}}=\frac{d(1+\gamma )}{d+2(\gamma -1)}\;,\;d\ge 2\;\;\textrm{or}\;\;0<d<2\;\;\textrm{and}\;\;1<\gamma <\gamma _c=\frac{2}{2-d}\;. \end{aligned}$$

In any dimension $\xi _\textrm{FRSB}$ is a decreasing function of $\gamma$ with $\xi _\textrm{FRSB}\rightarrow 2$ as $\gamma \rightarrow 1$ and $\xi _\textrm{FRSB}\rightarrow 1$ as $\gamma \rightarrow \gamma _c$ in dimension $0<d<2$ and $\xi _\textrm{FRSB}\rightarrow d/2$ as $\gamma \rightarrow \infty$ for $4>d\ge 2$.

We now provide detailed results on the large deviation function of the ground-state energy.

### Ground State Energy Large Deviation Function for Short-Ranged Correlated Disorder

The LDF of the ground-state energy $e_{\min }$ is defined in Eq. (12) displays several branches reflecting different pattern of replica symmetry breaking. In this section, we provide explicit results for the expression of these branches for short-ranged correlation functions *f*(*q*).

#### Replica-Symmetric Branch

The expression of the large deviation function within the replica-symmetric (RS) ansatz can be obtained either by applying the replica method or using the lower bound obtained from applying the Kac-Rice method. The RS branch can be expressed parametrically as

46 $$\begin{aligned} \Lambda _{L,\mathrm RS}(e,\mu )=&\frac{s^2}{2}f\left( 0\right) -\frac{1}{2}\int _\textbf{k}\left[ \frac{s f'(0)}{\nu (\textbf{k})-s f'(0)}+\ln \left( 1-\frac{s f'(0)}{\nu (\textbf{k})}\right) \right] \;,\end{aligned}$$

47 $$\begin{aligned} \textrm{where}\;\;e=&-s f(0)+\frac{f'(0)}{2}\int _\textbf{k}\frac{1}{\nu (\textbf{k})-s f'(0)}\;. \end{aligned}$$

This RS solution is only stable for $s\ge s_\textrm{AT}=(\mu -\mu _c)/f'(0)$ or conversely $e\le e_\textrm{AT}$ obtained by inserting the value of $s_\textrm{AT}$ in the expression for $\epsilon$ above. The value of $s_\textrm{AT}$ is found to satisfy

48 $$\begin{aligned} f''(0)\int _\textbf{k}\frac{1}{(\nu (\textbf{k})-s_\textrm{AT}f'(0))^2}=f''(0)I_2(\mu -s_\textrm{AT}f'(0))=1\;,\;\;\mathrm{i.e.}\;\;s_\textrm{AT}=\frac{\mu -\mu _c}{f'(0)}\;, \end{aligned}$$

where the last identity stems from $\mu _c$ being the (unique) solution to $f''(0)I_2(\mu _c)=1$. The corresponding energy value can be computed as

49 $$\begin{aligned} e_\textrm{AT}=(\mu _c-\mu )\frac{f(0)}{f'(0)}+\frac{f'(0)}{2}I_1(\mu _c)\;. \end{aligned}$$

Using that for a short range correlation function $f(0)f''(0)-f'(0)^2/2>0$, the CGF is shown to be convex in the regime of stability of the RS ansatz $s\ge s_\textrm{AT}$. The RS branch of the LDF is similarly convex in the region $e\le e_\textrm{AT}$. For $\mu >\mu _c$, one has that $s_\textrm{AT}<0$ and the typical ground-state energy takes its replica-symmetric expression $e_{L,\mathrm typ}(\mu )=f'(0)I_1(\mu )/2$. The behaviour of the LDF in the vicinity of its typical value can be investigated using Eqs. (46-47), yielding to the second order

50 $$\begin{aligned} \Lambda _{L,\mathrm RS}(e,\mu )&=\frac{\displaystyle \left( e-e_{L,\mathrm RS}(\mu )\right) ^2}{\displaystyle 2\mathcal{V}_L(\mu )}+O\left( e-e_{L,\mathrm RS}(\mu )\right) ^3\;,\end{aligned}$$

51 $$\begin{aligned} e_{L,\mathrm RS}(\mu )&=\frac{f'(0)I_1(\mu )}{2}\;,\;\;\mathcal{V}_L(\mu )=f(0)-I_2(\mu )\frac{f'(0)^2}{2}\;. \end{aligned}$$

The denominator in this expression can be identified as the RS expression of the rescaled variance in Eq. (96). The quadratic behaviour of the LDF is consistent with the central limit theorem describing the typical fluctuations of $e_{\min }$. The asymptotic behaviour of the large deviation function as $e\rightarrow -\infty$ is universal and reads

52 $$\begin{aligned} \Lambda _{L,\mathrm RS}(e,\mu )\approx \frac{e^2}{2f(0)}\;,\;\;e\rightarrow -\infty \;. \end{aligned}$$

Let us now consider the results obtained for branches with replica-symmetry breaking, starting with the case of one-step replica-symmetry breaking.

#### One Step Replica Symmetry Broken Branch

The 1RSB branch of the large deviation function can be obtained parametrically as well using the identities

53 $$\begin{aligned} \Lambda _{L,\mathrm 1RSB}(e,\mu )&=\frac{s^2}{2}f\left( Q\right) -\frac{1}{2}\int _\textbf{k}\left[ \ln \left( 1-\frac{s f'(Q)}{\nu (\textbf{k})}\right) +\frac{s f'(Q)}{\nu (\textbf{k})-s f'(Q)}\right] \;,\end{aligned}$$

54 $$\begin{aligned} \textrm{where}\;\;e&=-w f(0)-(s-w)\left( f(Q)-\frac{Q f'(Q)}{2}\right) \nonumber \\&\quad +\frac{f'(0)}{2}\int _\textbf{k}\frac{1}{\nu (\textbf{k})-s f'(Q)+w(f'(Q)-f'(0))}\;. \end{aligned}$$

The parameters *w* and *Q* are obtained for fixed *e* (or equivalently fixed *s*) as the solutions to the following equations

55 $$\begin{aligned}&Q=\frac{1}{w}\int _\textbf{k}\left[ \frac{1}{\nu (\textbf{k})-s f'(Q)}-\frac{1}{\nu (\textbf{k})-s f'(Q)+w(f'(Q)-f'(0))}\right] \;,\end{aligned}$$

56 $$\begin{aligned}&f(0)-f(Q)+Q f'(Q)\nonumber \\&\quad =\frac{1}{w^2}\int _\textbf{k}\left[ \ln \left( 1+\frac{w(f'(Q)-f'(0))}{\nu (\textbf{k})-s f'(Q)}\right) -\frac{w(f'(Q)-f'(0))}{\nu (\textbf{k})-s f'(Q)+w(f'(Q)-f'(0))}\right] \;. \end{aligned}$$

Note that $Q=0$ and $w=s$ is a trivial solution of the above equations and inserting these values Eqs. (53-54), the parametric expression for the RS branch of the large deviation function in Eqs. (46-47) is recovered. Thus, the 1RSB branch can only be obtained from the non-trivial solution of Eqs. (55-56).

For a mass $\mu <\mu _\textrm{dis}$, where the value of $\mu _\textrm{dis}$ reads

57 $$\begin{aligned} \mu _\textrm{dis}=\mu _c-\frac{f'(0)f^{(3)}(0)}{2I_3(\mu _c)f''(0)^3}\;, \end{aligned}$$

the transition from the RS to the 1RSB branch of the CGF occurs as $w\rightarrow s=s_\textrm{dis}$ and the parameter *Q* is discontinuous at the transition taking the value 0 within the RS phase and $Q_\textrm{dis}>0$ in the 1RSB phase. The parameters $s_\textrm{dis},Q_\textrm{dis}$ of the transition are obtained by solving the following equations

58 $$\begin{aligned}&Q_\textrm{dis}=\frac{1}{s_\textrm{dis}}\int _\textbf{k}\left[ \frac{1}{\nu (\textbf{k})-s_\textrm{dis} f'(Q_\textrm{dis})}-\frac{1}{\nu (\textbf{k})-s_\textrm{dis} f'(0)}\right] \;,\end{aligned}$$

59 $$\begin{aligned}&f(0)-f(Q_\textrm{dis})+Q_\textrm{dis} f'(Q_\textrm{dis})=\frac{1}{s_\textrm{dis}^2}\int _\textbf{k}\left[ \ln \frac{\nu (\textbf{k})-s_\textrm{dis} f'(0)}{\nu (\textbf{k})-s_\textrm{dis} f'(Q_\textrm{dis})}-\frac{s_\textrm{dis}(f'(Q_\textrm{dis})-f'(0))}{\nu (\textbf{k})-s_\textrm{dis} f'(0)}\right] \;. \end{aligned}$$

The corresponding transition in the large deviation function occurs at the energy

60 $$\begin{aligned} e_\textrm{dis}=-s_\textrm{dis} f(0)+\frac{f'(0)}{2}\int _\textbf{k}\frac{1}{\nu (\textbf{k})-s_\textrm{dis} f'(0)}\;, \end{aligned}$$

is of second order and takes the form

61 $$\begin{aligned}&\Lambda _{L,\mathrm 1RSB}(e,\mu )-\Lambda _{L,\mathrm RS}(e,\mu )\nonumber \\&\quad =\frac{1}{2}\left( \frac{1}{\partial _s^2\varphi _{L,\mathrm 1RSB}(s_\textrm{dis},\mu )}-\frac{1}{\partial _s^2\varphi _{L,\mathrm RS}(s_\textrm{dis},\mu )}\right) \left( e-e_\textrm{dis}\right) ^2+O(e-e_\textrm{dis})^3\;, \end{aligned}$$

where the values of $\partial _s^2\varphi _{L,\mathrm 1RSB}(s_\textrm{dis},\mu )$ and $\partial _s^2\varphi _{L,\mathrm RS}(s_\textrm{dis},\mu )$ are given explicitly in Eq. (164).

For a mass $\mu >\mu _\textrm{dis}$, the transition for the CGF occurs as $Q\rightarrow 0$ vanishes continuously, corresponding to $s\rightarrow s_\textrm{AT}$, while *w* takes the value $w_\textrm{AT}=-f^{(3)}(0)/(2(f''(0))^3 I_3(\mu _c))\ge s_\textrm{AT}$ at the transition. The corresponding transition for the large deviation function occurs at the energy $e_\textrm{AT}$ defined in Eq. (49)

**Fig. 2:**
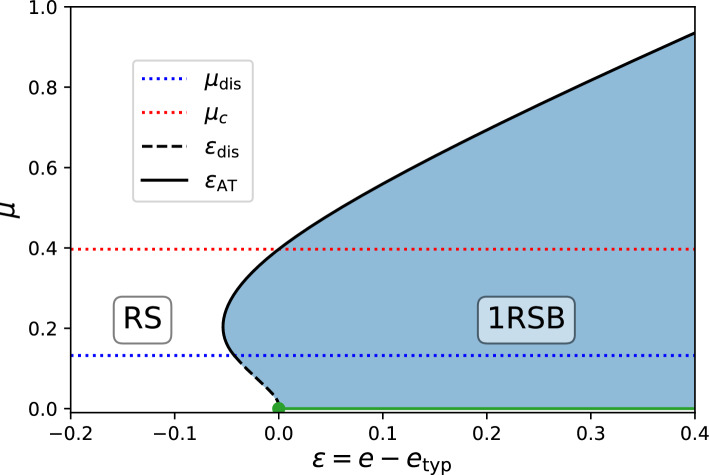
Phase diagram of the large deviation function $\mathcal{L}(\epsilon ,\mu )=\lim _{L\rightarrow \infty }\Lambda _L(e_\textrm{typ}+\epsilon ,\mu )$ for the centred ground state energy $\epsilon _{\min }=e_{\min }-e_\textrm{typ}$ of the elastic manifold $d=1$ with exponential correlation function $f_{\infty }(q)=\exp (-q)$ (see section 5.1 for details). For any fixed value of $\mu$, the RS phase (white) and 1RSB phase (blue) are separated by a transition line. The transition occurs at $\epsilon =\epsilon _\textrm{dis}$ given by Eq. (230) (dashed black curve) for $\mu \le \mu _\textrm{dis}$ (indicated by the horizontal dotted blue line) and is of second order while for $\mu \ge \mu _\textrm{dis}$ it is of third order and occurs at $\epsilon _\textrm{AT}$ given by Eq. (230) (solid black curve). The typical value of the centred energy $\epsilon _\textrm{typ}$, where the LDF achieves its unique zero, corresponds to the vertical line $\epsilon =0$. For $\mu \ge \mu _{c}$ (indicated by the horizontal dotted red line), it is given by its RS expression while for $\mu <\mu _{c}$ it is given by its 1RSB expression. For $\mu =0$, one expects that the large deviation function of speed *N* is only given by its RS expression and describes any $\epsilon \le 0$. On the other hand, the fluctuations for $\epsilon \ge 0$ (indicated by the green line) are described by a large deviation regime with higher speed

The continuous transition occurring as $Q\rightarrow 0$, i.e. as the trivial and non-trivial solutions of the above equation match, occurs precisely at energy $e=e_\textrm{AT}$. This continuous transition leads to a third order transition in the large deviation function

62 $$\begin{aligned} \Lambda _{L,\mathrm 1RSB}(e,\mu )-\Lambda _{L,\mathrm RS}(e,\mu )=I_3(\mu _c)\frac{f'(0)^3(\mu -\mu _c)}{6\mathcal{V}_L(\mu _c)^3(\mu -\mu _\textrm{dis})}\left( e-e_\textrm{AT}\right) ^3+O(e-e_\textrm{AT})^4\;, \end{aligned}$$

where $\mathcal{V}_L(\mu _c)=f(0)-f'(0)^2/(2f''(0)$, we used the identity $\mu _c=\mu -s_\textrm{AT} f'(0)$ and we remind that $I_p(x)$ is defined in Eq. (15). In Fig. 2, we show the phase diagram of the large deviation function, where the RS phase (in white) is separated from the 1RSB phase (in blue) by the transition line (black curve). For $\mu < \mu _\textrm{dis}$, the transition is discontinuous (dashed curve), i.e. $Q_\textrm{dis}>0$ at the transition while it is continuous (solid curve) for $\mu > \mu _\textrm{dis}$.

In the vicinity of the typical value, the large deviation function vanishes quadratically

63 $$\begin{aligned} \Lambda _{L,\mathrm 1RSB}(e,\mu )&=\frac{\displaystyle \left( e-e_{L,\mathrm 1RSB}(\mu )\right) ^2}{\displaystyle 2\mathcal{V}_L(\mu )}+O\left( e-e_{L,\mathrm 1RSB}(\mu )\right) ^3\;,\end{aligned}$$

64 $$\begin{aligned} e_{L,\mathrm 1RSB}(\mu )&=\frac{f'(0)I_1(\mu )}{2}+w_\textrm{typ}\left( f(Q_\textrm{typ})-f(0)-\frac{Q_\textrm{typ}}{2}(f'(Q_\textrm{typ})+f'(0))\right) \;,\end{aligned}$$

65 $$\begin{aligned} \mathcal{V}_L(\mu )&=f(Q_\textrm{typ})-I_2(\mu )\frac{f'(Q_\textrm{typ})^2}{2}\;. \end{aligned}$$

and the pair $Q_\textrm{typ},w_\textrm{typ}$ satisfy the integral equations

66 $$\begin{aligned} Q_\textrm{typ}&=\frac{1}{w_\textrm{typ}}\int _\textbf{k}\left[ \frac{1}{\nu (\textbf{k})}-\frac{1}{\nu (\textbf{k})+w_\textrm{typ}(f'(Q_\textrm{typ})-f'(0))}\right] \;,\end{aligned}$$

67 $$\begin{aligned}&f(0)-f(Q_\textrm{typ})+Q_\textrm{typ} f'(Q_\textrm{typ}) \nonumber \\&=\frac{1}{w_\textrm{typ}^2}\int _\textbf{k}\left[ \ln \left( 1+\frac{w_\textrm{typ}(f'(Q_\textrm{typ})-f'(0))}{\nu (\textbf{k})}\right) -\frac{w_\textrm{typ}(f'(Q_\textrm{typ})-f'(0))}{\nu (\textbf{k})+w_\textrm{typ}(f'(Q_\textrm{typ})-f'(0))}\right] \;. \end{aligned}$$

This behaviour is compatible with a smooth matching between the large deviation regime and the tail of the Gaussian distribution of typical fluctuations.

Finally, the asymptotic behaviour of the large deviation function as $e\rightarrow +\infty$ can be obtained and shown to be non universal, in contrast to the behaviour as $e\rightarrow -\infty$ (see Eq. (52)). A careful analysis shows that in this regime $Q\rightarrow \infty$ with the scaling $s=\mu /f'(Q)+O(1)$ and $e=\mu Q/2+O(1)$ while the parameter *w* reaches an asymptotic value $w_{\infty }=O(1)$. The large deviation function thus behaves as

68 $$\begin{aligned} \Lambda _{L,\mathrm 1RSB}(e,\mu )\approx \frac{\displaystyle \mu ^2 f\left( 2e/\mu \right) }{\displaystyle 2f'\left( 2e/\mu \right) ^2}\;,\;\;e\rightarrow +\infty \;. \end{aligned}$$

This result concludes our results on the one-step replica symmetry broken branch. Next we describe our results for th full replica symmetry broken branch.

#### Full Replica Symmetry Broken Branch

The FRSB branch of the large deviation function can be expressed parametrically as

69 $$\begin{aligned} \Lambda _{L,\mathrm FRSB}(e,\mu )=&\frac{s^2}{2}f\left( Q\right) -\frac{1}{2}\int _\textbf{k}\left[ \ln \left( 1-\frac{s f'(Q)}{\nu (\textbf{k})}\right) +\frac{s f'(Q)}{\nu (\textbf{k})-s f'(Q)}\right] \;,\;\;\textrm{where}\;\; \nonumber \\&\int _\textbf{k}\frac{f''(Q)}{(\nu (\textbf{k})-s f'(Q))^2}=1\;,\end{aligned}$$

70 $$\begin{aligned} \textrm{and}\;\;e=&-s f(0)-\frac{1}{2}\int _0^{Q}(z(q)-s)\left[ q f''(q)-f'(q)\right] \nonumber \\&+\frac{f'(0)}{2}\int _\textbf{k}\frac{1}{\nu (\textbf{k})-s f'(Q)+\int _0^{Q}z(q)f''(q)dq}\;. \end{aligned}$$

The function *z*(*q*) appearing in the expression above satisfies

71 $$\begin{aligned} \forall q\in [0,Q]\;,\;\;\int _\textbf{k}\frac{f''(q)}{(\nu (\textbf{k})-s f'(Q)+\int _q^{Q}z(r)f''(r)dr)^2}=1\;. \end{aligned}$$

The transition between the RS and FRSB branches occurs as $e\rightarrow e_\textrm{AT}$ and is of third order

72 $$\begin{aligned} \Lambda _{L,\mathrm FRSB}(e,\mu )-\Lambda _{L,\mathrm RS}(e,\mu )=I_3(\mu _c)\frac{f'(0)^3 s_\textrm{AT}}{6\mathcal{V}_L(\mu _c)^3(s_\textrm{AT}-z(0))}\left( e-e_\textrm{AT}\right) ^3+O(e-e_\textrm{AT})^4\;. \end{aligned}$$

**Fig. 3:**
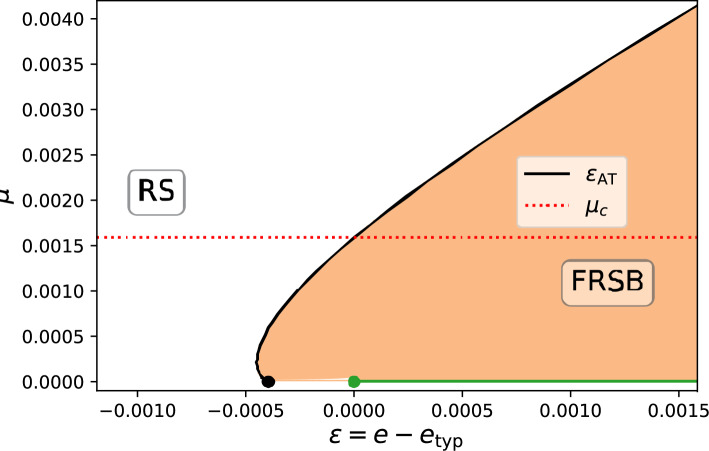
Phase diagram of the large deviation function $\mathcal{L}(\epsilon ,\mu )=\lim _{L\rightarrow \infty }\Lambda _L(e_\textrm{typ}+\epsilon ,\mu )$ for the centred ground state energy $\epsilon _{\min }=e_{\min }-e_\textrm{typ}$ of the elastic manifold of internal dimension $d=3$ with elasticity term $\nu (\textbf{k})=\mu +\textbf{k}^2$ and exponential correlation function $f_{\infty }(q)=\exp (-q)$ (see section 5.3 for details). For any fixed value of $\mu$, the RS phase (white) and FRSB phase (orange) are separated by a transition line. The transition occurs at $\epsilon =\epsilon _\textrm{AT}$ given by Eq. (245) (solid black curve) and is of third order. The typical value of the centred energy $\epsilon _\textrm{typ}$, where the LDF achieves its unique zero, corresponds to the value $\epsilon =0$. For $\mu \ge \mu _{c}$ (indicated by the horizontal dotted red line), it is given by its RS expression while for $\mu <\mu _{c}$ it is given by its FRSB expression. For $\mu =0$, in addition to the RS phase (for energies below the black dot) and the FRSB phase (for energies between the black and green dot), an additional phase appears (represented by the solid green line) where the large deviation of speed $NL^d$ is infinite and a large deviation regime with higher speed describes the fluctuations

In Fig. 3, we show the phase diagram of the large deviation function, where the RS phase (in white) is separated from the FRSB phase (in orange) by the transition line (black curve). The transition between a RS and FRSB phase is always continuous.

For any $\mu >0$, the large deviation function vanishes quadratically in the vicinity of the typical value with

73 $$\begin{aligned} \Lambda _{L,\mathrm 1RSB}(e,\mu )&=\frac{\displaystyle \left( e-e_{L,\mathrm FRSB}(\mu )\right) ^2}{\displaystyle 2\mathcal{V}_L(\mu )}+O\left( e-e_{L,\mathrm 1RSB}(\mu )\right) ^3\;,\end{aligned}$$

74 $$\begin{aligned} e_{L,\mathrm FRSB}(\mu )&=-\frac{1}{2}\int _0^{Q_\textrm{typ}}dq\,z_\textrm{typ}(q)\left[ q f''(q)-f'(q)\right] \nonumber \\&\quad +\frac{f'(0)}{2}\int _\textbf{k}\frac{1}{\nu (\textbf{k})+\int _0^{Q_\textrm{typ}}z_\textrm{typ}(q)f''(q)dq}\;,\end{aligned}$$

75 $$\begin{aligned} \mathcal{V}_L(\mu )&=f(Q_\textrm{typ})-I_2(\mu )\frac{f'(Q_\textrm{typ})^2}{2}\;,\;\;\textrm{where}\;\;Q_\textrm{typ}={f''}^{-1}\left( \frac{1}{I_2(\mu )}\right) \;,\end{aligned}$$

76 $$\begin{aligned} \textrm{and}\;\;\forall q\in [0,Q_\textrm{typ}]&\;,\;\;\int _\textbf{k}\frac{f''(q)}{(\nu (\textbf{k})+\int _q^{Q_\textrm{typ}}z_\textrm{typ}(r)f''(r)dr)^2}=1\;. \end{aligned}$$

The expression above is fully consistent with the ground state energy $e_{\min }$ satisfying the central limit theorem.

Finally, the asymptotic behaviour of the large deviation function as $e\rightarrow +\infty$ can be explored. The asymptotic behaviour is exactly the same as for the 1RSB case in Eq. (68). Note that this behaviour is qualitatively very different from the large deviation function of spherical spin-glasses. For the latter, as conjectured in [21] and subsequently proved rigorously in [22], the large deviation function with speed *N* of the ground-state energy diverges at a finite value of energy indicating a transition towards a regime of large deviation with speed $N^2$ (see also [23–25] for similar results for the SK model). The presence of the harmonic confinement seems to prevent the occurrence of such transition for the elastic manifold. Next we will summarise the results in the limit of zero confinement, where that transition is instead expected to occur.

### Massless Limit and Superconcentration

In this section, we consider the large deviation of the centred ground-state energy $\epsilon _{\min }=e_{\min }-e_\textrm{typ}$ in the massless limit $\mu \rightarrow 0$ and continuum limit $L\rightarrow \infty$. To this end, we study

77 $$\begin{aligned} \mathcal{L}(\epsilon )=\lim _{L\rightarrow \infty }\Lambda _L(e_\textrm{typ}(L,0)+\epsilon ,0)\;. \end{aligned}$$

To make our computation as explicit as possible, we will consider the following covariance function

78 $$\begin{aligned} f_{\gamma }(q)=\left( 1+\frac{q}{\gamma -1}\right) ^{1-\gamma }\;,\;\;\gamma > 1\;. \end{aligned}$$

Note that the asymptotic behaviour of this function is not exactly that of Eq. (34) although quite similar but it has the advantage to have a well-defined limit $f_{\infty }(q)=e^{-q}$ as $\gamma \rightarrow \infty$.

#### FRSB Branch

To prevent divergence of the expressions, we will work in the following with the variable $\epsilon =e-e_\textrm{typ}$ which remains of order *O*(1) in the limit $\mu \rightarrow 0$ in any dimension. Let us first consider the case $d\ge 2$ or $1\le \gamma \le 2/(d-2)$ where the large deviation function is given in the vicinity of its typical value by its FRSB branch. From the definition of *Q* in that phase in Eq. (69), the value of *s* can be expressed as a function of *Q* in the limit $\mu \rightarrow 0$ as

79 $$\begin{aligned} s=-\frac{1}{f'(Q)}\left( \frac{\displaystyle f''(Q)}{\displaystyle B_d}\right) ^{\frac{2}{4-d}}\;, \end{aligned}$$

where $B_d=-2^{d}\pi ^{\frac{d}{2}-1}\Gamma \left( \frac{d}{2}-1\right) \sin \left( \frac{d\pi }{2}\right)$. Note that $B_2=4\pi$ is finite. Inserting this expression for *s* in Eq. (69), the large deviation function and energy can then both be expressed parametrically in terms of the sole parameter *Q* as

80 $$\begin{aligned} \mathcal{L}_\textrm{FRSB}(\epsilon )=\left( \frac{f''(Q)}{B_d}\right) ^{\frac{4}{4-d}}\left( \frac{f(Q)}{2f'(Q)^2}-\frac{1}{d\,f''(Q)}\right) \;. \end{aligned}$$

Using the explicit expression for the function *z*(*q*) in Eq. (192), the energy can similarly be expressed as

81 $$\begin{aligned} \epsilon =&\left( \frac{\displaystyle f''(Q)}{\displaystyle B_d}\right) ^{\frac{2}{4-d}}\left( \frac{f(Q)}{f'(Q)}-\frac{f'(Q)}{(d-2)f''(Q)}\right) -\frac{4-d}{2(d-2)}\int _{Q}^{\infty }dq\left( \frac{\displaystyle f''(q)}{\displaystyle B_d}\right) ^{\frac{2}{4-d}}\;. \end{aligned}$$

One may expect the expression above to diverge in $d=2$. However, the diverging terms cancel, yielding the finite expression

82 $$\begin{aligned} \epsilon =\frac{1}{4\pi }\left[ \frac{f(Q)f''(Q)}{f'(Q)}-\frac{f'(Q)}{2}-\frac{1}{2}\int _{Q}^{\infty }dq \frac{f'(q)f^{(3)}(q)}{f''(q)}\right] \;,\;\;d=2\;. \end{aligned}$$

Finally, using these expressions for the explicit covariance function $f_{\gamma }(q)$, one obtains the following expression

83 $$\begin{aligned} \mathcal{L}_\textrm{FRSB}(\epsilon )=\frac{1}{\xi }\left( \left( \frac{\gamma }{B_d(\gamma -1)}\right) ^{\frac{2}{4-d}}\frac{\xi (2+\gamma (d-2))}{2d\gamma }\right) ^{\xi -1}(-\epsilon )^{\xi }\;,\;\;\xi =\frac{d(1+\gamma )}{d+2(\gamma -1)}\;. \end{aligned}$$

Note that this expression for the large deviation function is only valid in the FRSB phase, i.e. for energies $\epsilon$ larger than

84 $$\begin{aligned} \epsilon _\textrm{AT}=\left( \frac{\displaystyle f''(0)}{\displaystyle B_d}\right) ^{\frac{2}{4-d}}\left( \frac{f(0)}{f'(0)}-\frac{f'(0)}{(d-2)f''(0)}\right) -\frac{4-d}{2(d-2)}\int _{0}^{\infty }dq\left( \frac{\displaystyle f''(q)}{\displaystyle B_d}\right) ^{\frac{2}{4-d}}\;. \end{aligned}$$

For fluctuations with $\epsilon <\epsilon _\textrm{AT}$, one needs to use the expression for the RS branch.

#### 1RSB Branch

The behaviour of the large deviation for $d<2$ and $\gamma \ge 2/(d-2)$ can be understood by analogy with the study of spherical spin glasses in [21]. It was found there that the full large deviation function is given at zero magnetic field by its RS branch while the 1RSB phase is limited to a point. One can check similarly here that the large deviation function undergoes a discontinuous transition at the value $e_\textrm{dis}\equiv e_\textrm{typ}$ from $Q=0$ in the RS phase to $Q\rightarrow \infty$ in the 1RSB phase. In the RS phase, the large deviation function can be obtained in the simple parametric form

85 $$\begin{aligned} \mathcal{L}_\textrm{RS}(\epsilon )=\frac{s^2}{2}f(0)-\frac{\displaystyle \left( -s f'(0)\right) ^{\frac{d}{2}}}{\displaystyle d B_d}\;, \end{aligned}$$

where the energy difference reads

86 $$\begin{aligned} \epsilon =(w_\textrm{typ}-s)f(0)-\frac{f'(0)}{(2-d)B_d}\left[ \left( -w_\textrm{typ}f'(0)\right) ^{\frac{d}{2}-1}-\left( -sf'(0)\right) ^{\frac{d}{2}-1}\right] \;. \end{aligned}$$

Note that here as well, one may expect this expression to diverge in $d=2$, however the diverging terms cancel, yielding the finite expression

87 $$\begin{aligned} \epsilon =(w_\textrm{typ}-s)f(0)-\frac{f'(0)}{8\pi }\ln \frac{s}{w_\textrm{typ}}\;,\;\;d=2\;. \end{aligned}$$

Both the LDF $\mathcal{L}_\textrm{RS}(\epsilon )$ and the expression of the energy difference $\epsilon$ must vanish as $s\rightarrow w_\textrm{typ}$, which can simply be obtained from the expression of the LDF as

88 $$\begin{aligned} w_\textrm{typ}=\left( \frac{2 (-f'(0))^{\frac{d}{2}}}{d B_d f(0)}\right) ^{\frac{2}{4-d}}\;. \end{aligned}$$

Finally, one can check that the large deviation function vanishes linearly as the free energy difference vanishes, i.e.

89 $$\begin{aligned} \mathcal{L}_\textrm{RS}(\epsilon )=-w_\textrm{typ}\epsilon +O(\epsilon )^2\;. \end{aligned}$$

As we show in appendix B, the RS expression for the LDF can be simply expressed in terms of the annealed complexity of minima $\Sigma _{\min }(e,\mu )$, i.e.

90 $$\begin{aligned} \mathcal{L}_\textrm{RS}(\epsilon )=-\Sigma _{\min }(e_\textrm{typ}+\epsilon ,0)\;. \end{aligned}$$

Thus, in the limit $\mu \rightarrow 0$ studied here, one can check that the typical energy $e_\textrm{typ}$ coincides with the lowest argument value where the annealed complexity vanishes. This property was first obtained for the pure spherical *p*-spin in [26] and confirmed more recently for arbitrary mixture in [21, 22].

### Long-Range Disorder

In this section, we summarise the results obtained for the cumulant generating function of the ground-state energy difference $\Delta e_{\min }$. The latter reads

91 $$\begin{aligned} \phi _L(s,\mu )=\lim _{NL^d\rightarrow \infty }\frac{1}{N L^d}\ln \left\langle e^{-s N L^d \Delta \epsilon _{\min }} \right\rangle ={\left\{ \begin{array}{ll} \displaystyle \max _{g(\textbf{k},z),G_0(\textbf{k}),v(\textbf{k}),w_M}\mathcal{S}_{1,L}(\mu )& \;,\;\;s<0\;, \\ 0& \;,\;\;s=0\;, \\ \displaystyle \min _{g(\textbf{k},z),G_0(\textbf{k}),v(\textbf{k}),w_M}\mathcal{S}_{1,L}(\mu )& \;,\;\;s>0\;, \end{array}\right. } \end{aligned}$$

where the functional $\mathcal{S}_{1,L}(\mu )$ reads

92 $$\begin{aligned} \mathcal{S}_{1,L}(\mu )=&-\frac{s}{2}\int _\textbf{k}\nu (\textbf{k})\left[ g(\textbf{k},s)+G_0(\textbf{k})\right] +\frac{s^2}{2}\left[ f(0)-2f\left( \frac{1}{2}\int _\textbf{k}\,\left[ g(\textbf{k},s)+G_0(\textbf{k})\right] \right) \right] \end{aligned}$$

93 $$\begin{aligned}&+\frac{s}{2}\left[ -f'(0)\int _\textbf{k}v(\textbf{k})+w_M\,f\left( 0\right) -\int _{s}^{w_M}dz\,f\left( \int _\textbf{k}g(\textbf{k},z)\right) \right] \nonumber \\&+\frac{1}{2}\int _\textbf{k}\left[ -\frac{s}{w_M}\ln v(\textbf{k})+\ln \left( \lambda (\textbf{k},s)+s G_0(\textbf{k})\right) -s\int _{s}^{w_M}\frac{dz}{z^2}\ln \lambda (\textbf{k},z)\right] \;,\nonumber \\ \textrm{where}&\;\;\lambda (\textbf{k},z)=v(\textbf{k})+z\,g(\textbf{k},z)+\int _{z}^{w_M}dw\,g(\textbf{k},w)\;. \end{aligned}$$

As in the case of the short-range disorder, the (breaking point) parameter $w_M\ge s$, the functions $v(\textbf{k})\ge 0$ and $G_0(\textbf{k})\ge 0$ are positive and the function $g(\textbf{k},z)$ is a positive non-increasing function defined over the interval $z\in [s,w_M]$. Optimising over $w_M$ yields the explicit boundary condition $g(\textbf{k},z\ge w_M)=0$. The expression above is valid for any pattern of replica-symmetry breaking (replica-symmetric ($w_M=s$), one-step replica symmetry breaking, full replica-symmetry breaking).

The average/typical ground state energy value takes the same form as for short-range disorder in Eq. (28). The expression for the cumulant generating function obtained above allows to derive higher-order cumulants and the rescaled variance in particular. The latter is defined as

94 $$\begin{aligned} \mathcal{V}_{L}^\mathrm{l.r.}(\mu )=&\lim _{N L^d\rightarrow \infty }N L^d \textrm{Var}(\Delta e_{\min })=\partial _s^2\phi _L(0,\mu )\nonumber \\ =&f\left( Q_\textrm{typ}\right) +f(0)-2 f\left( \frac{Q_\textrm{typ}-I_2(\mu ) f'\left( Q_\textrm{typ}\right) }{2}\right) -\frac{I_2(\mu ) f'\left( Q_\textrm{typ}\right) ^2}{2}\;,\;\; \nonumber \\&\textrm{where}\;\;Q_\textrm{typ}=\int _\textbf{k}g_\textrm{typ}(\textbf{k},0)\;. \end{aligned}$$

The integral $I_2(x)$ is defined in Eq. (15). The expression in Eq. (30) is valid at any level of RSB while the expression of $Q_\textrm{typ}$ depends instead on the level of replica symmetry breaking. In the simplest, replica-symmetric (RS) phase $Q_\textrm{typ}=0$. In the full replica symmetry broken (FRSB) phase it reads instead $Q_\textrm{typ}={f''}^{-1}\left( 1/I_2(\mu )\right)$, i.e. it is expressed from the functional inverse of the second derivative of the covariance function. Finally, in the one-step replica symmetry broken (1RSB) phase it is obtained as the non-zero solution of the following set of self-consistent equations (eliminating the breaking-point parameter $w_\textrm{typ}$ in the following equations)

95 $$\begin{aligned} Q_\textrm{typ}&=\int _\textbf{k}\frac{1}{w_\textrm{typ}}\left( \frac{1}{\nu (\textbf{k})}-\frac{1}{\nu (\textbf{k})+w_\textrm{typ}(f'\left( Q_\textrm{typ}\right) -f'(0))}\right) \;,\end{aligned}$$

96 $$\begin{aligned}&f\left( Q_\textrm{typ}\right) -f(0)-Q_\textrm{typ} f'\left( Q_\textrm{typ}\right) \nonumber \\&=\frac{1}{w_\textrm{typ}^2}\int _\textbf{k}\left[ \frac{w_\textrm{typ}(f'\left( Q_\textrm{typ}\right) -f'(0))}{\nu (\textbf{k})+w_\textrm{typ}(f'\left( Q_\textrm{typ}\right) -f'(0))}-\ln \left( 1+\frac{w_\textrm{typ}(f'\left( Q_\textrm{typ}\right) -f'(0))}{\nu (\textbf{k})}\right) \right] \;. \end{aligned}$$

## Optimisation Problem

In this section, we derive the general expression of the cumulant generating function for the ground-state energy in terms of a functional optimisation problem.

### Integer Moments

To compute the large deviation function, we first compute exactly the integer moments of the partition function. The integer moments of the centred free-energy read [13]

97 $$\begin{aligned}&\left\langle Z_{N,L,\eta }(\beta )^n \right\rangle \nonumber \\&=\int \prod _{a=1}^n \mathcal{D}  \textbf{u}_a(\textbf{x})\,\left\langle e^{-\beta \sum _{a=1}^n \left( \mathcal{H}[\textbf{u}_a]-\eta \mathcal{H}[\textbf{0}]\right) } \right\rangle \nonumber \\ =&\int \prod _{a=1}^n \mathcal{D}  \textbf{u}_a(\textbf{x})\,\exp \left[ \frac{N}{2}\left( \beta ^2\sum _\textbf{x}\sum _{a,b}\left[ f\left( \frac{(\textbf{u}_a(\textbf{x})-\textbf{u}_b(\textbf{x}))^2}{2N}\right) -2\eta f\left( \frac{\textbf{u}_a(\textbf{x})^2}{2N}\right) +\eta f(0)\right] \right. \right. \nonumber \\&\left. \left. -\frac{\beta }{2}\sum _{a}\sum _{\textbf{x},\textbf{y}}  \textbf{u}_a(\textbf{x})(\mu \delta _{\textbf{x},\textbf{y}}-t \Delta _{\textbf{x},\textbf{y}})\textbf{u}_a(\textbf{y})\right) \right] \;, \end{aligned}$$

where the parameter $\eta =0,1$ allows to treat on the same footing short-range and long-range disorder. This expression can be rewritten in terms of the fields in momentum space as $\textbf{u}_a(\textbf{k})$, which reads

98 $$\begin{aligned}&\left\langle Z_{N,L,\eta }(\beta )^n \right\rangle \nonumber \\&=\int \prod _{a=1}^n \mathcal{D}  \textbf{u}_a(\textbf{k})\,\exp \left[ \frac{N}{2}\left( \beta ^2\sum _\textbf{x}\sum _{a,b}\left[ f\left( \int _\textbf{k}\int _\mathbf{k'}\frac{(\textbf{u}_a(\textbf{k})-\textbf{u}_b(\textbf{k}))\cdot (\textbf{u}_a(\textbf{k}')-\textbf{u}_b(\textbf{k}'))}{2N}e^{i\textbf{x}\cdot (\textbf{k}+\textbf{k}')}\right) \right. \right. \right. \nonumber \\&\left. \left. \left. -2\eta f\left( \int _\textbf{k}\int _\mathbf{k'}\frac{\textbf{u}_a(\textbf{k})\cdot \textbf{u}_a(\textbf{k}')}{N}e^{i\textbf{x}\cdot (\textbf{k}+\textbf{k}')}\right) +\eta f(0)\right] -\frac{\beta L^d}{2}\sum _{a=1}^n\int _\textbf{k}\nu (\textbf{k})\frac{\textbf{u}_a(\textbf{k})\cdot \textbf{u}_a(-\textbf{k})}{N}\right) \right] \;. \end{aligned}$$

In Eq. (97), all the terms are invariant by translation in the spatial space $\textbf{x}$ such that we expect the overlaps not to depend explicitly on the position. Thus we can replace

99 $$\begin{aligned}&\sum _\textbf{x}\sum _{a,b} f\left( \int _\textbf{k}\int _\mathbf{k'}\frac{(\textbf{u}_a(\textbf{k})-\textbf{u}_b(\textbf{k}))\cdot (\textbf{u}_a(\textbf{k}')-\textbf{u}_b(\textbf{k}'))}{2N}e^{i\textbf{x}\cdot (\textbf{k}+\textbf{k}')}\right) \nonumber \\&\leftrightarrow L^d\sum _{a,b} f\left( \frac{1}{L^d}\sum _\textbf{x}\int _\textbf{k}\int _\mathbf{k'}\frac{(\textbf{u}_a(\textbf{k})-\textbf{u}_b(\textbf{k}))\cdot (\textbf{u}_a(\textbf{k}')-\textbf{u}_b(\textbf{k}'))}{2N}e^{i\textbf{x}\cdot (\textbf{k}+\textbf{k}')}\right) \nonumber \\&=L^d\sum _{a,b} f\left( \int _\textbf{k}\frac{(\textbf{u}_a(\textbf{k})-\textbf{u}_b(\textbf{k}))\cdot (\textbf{u}_a(-\textbf{k})-\textbf{u}_b(-\textbf{k}))}{2N}\right) \;. \end{aligned}$$

Using this hypothesis, one can express explicitly the integer moments of the partition function in terms of the overlap matrix $\textbf{G}_{ab}(\textbf{k})=(\textbf{u}_a(\textbf{k})\cdot \textbf{u}_b(-\textbf{k}))/N$ as

100 $$\begin{aligned} \left\langle Z_{N,L,\eta }(\beta )^n \right\rangle =&C_{N,n}\int \prod _{a,b=1}^n \mathcal{D}  \textbf{G}_{ab}(\textbf{k})\,\left( \det \textbf{G}(\textbf{k})\right) ^{\frac{n+1}{2}}\exp \left( N L^d S_n[\textbf{G}(\textbf{k})]\right) \;,\end{aligned}$$

101 $$\begin{aligned} S_n=&\frac{\beta ^2}{2}\sum _{a,b}\left[ f\left( \int _\textbf{k}\left[ \frac{\textbf{G}_{aa}(\textbf{k})+\textbf{G}_{bb}(\textbf{k})}{2}-\textbf{G}_{ab}(\textbf{k})\right] \right) -2\eta f\left( \int _\textbf{k}\frac{\textbf{G}_{aa}(\textbf{k})}{2}\right) +\eta f(0)\right] \end{aligned}$$

102 $$\begin{aligned}&+\frac{1}{2}\int _\textbf{k}\textrm{Tr}\ln \left[ \textbf{G}(\textbf{k})\right] -\frac{\beta }{2}\sum _{a=1}^n\int _\textbf{k}\nu (\textbf{k})\textbf{G}_{aa}(\textbf{k})+c_n\;,\nonumber \\ \textbf{G}_{ab}(\textbf{k})=&\frac{\textbf{u}_a(\textbf{k})\cdot \textbf{u}_b(-\textbf{k})}{N}\;. \end{aligned}$$

The expression for the moments of the partition function can be obtained at the leading (exponential) order in $NL^d$ by using a saddle-point method with respect to the matrix $\textbf{G}(\textbf{k})$, i.e.

103 $$\begin{aligned} \lim _{N\rightarrow \infty }\frac{1}{N L^d}\ln \left\langle Z_{N,L,\eta }(\beta )^n \right\rangle =\max _{\textbf{G}(\textbf{k})}S_{n,\eta }[\textbf{G}(\textbf{k})]\;. \end{aligned}$$

Supposing that the limit of high-dimension $NL^d \rightarrow \infty$ and zero temperature $\beta \rightarrow \infty$ can be exchanged, the scaled cumulant generating function of $e_{\min }$ (or $\Delta e_{\min }$ for long-range disorder) can be computed by taking the limit $n\rightarrow 0$ and $\beta \rightarrow \infty$ such that the parameter $s=n\beta$ is fixed, yielding

104 $$\begin{aligned} \lim _{\beta \rightarrow \infty }\lim _{N\rightarrow \infty }\frac{1}{N L^d}\ln \left\langle Z_{N,L,\eta }(\beta )^{s/\beta } \right\rangle =\lim _{N\rightarrow \infty }\frac{1}{N L^d}\ln \left\langle e^{-s NL^d(e_{\min }-\eta e_\textrm{flat})} \right\rangle \;, \end{aligned}$$

where $e_\textrm{flat}=\mathcal{H}[\textbf{0}]/(NL^d)$ is the intensive energy of the flat configuration $\textbf{u}(\textbf{x})=\textbf{0}$. From the expression of the cumulant generating function, we will be able to reconstruct the large deviation function by Legendre transform

105 $$\begin{aligned} \Lambda _L(e,\mu )=-\min _{s\in \mathbb {R}}\left[ s\epsilon +\varphi _L(s,\mu )\right] \;. \end{aligned}$$

In the following, we analyse this limit explicitly.

### Parisi Scheme

Let us suppose that the matrix $\textbf{G}(\textbf{k})$ takes the Parisi hierarchical form with *k* replica-symmetry breaking. We introduce the $k+2$ overlap functions $G_l(\textbf{k})$ for $l=0,\cdots ,k+1$ characterising the Parisi blocks satisfying

106 $$\begin{aligned} \forall \textbf{k}\,,\,\,G_0(\textbf{k})\le G_1(\textbf{k})\le \cdots \le G_k(\textbf{k})\le G_{k+1}(\textbf{k})=G_d(\textbf{k})\;. \end{aligned}$$

We similarly introduce the parameters $m_l$ for $l=0,\cdots ,k+1$ encoding the size of the Parisi blocks with

107 $$\begin{aligned} m_0=n\ge m_1\ge \cdots \ge m_k\ge m_{k+1}=1\;. \end{aligned}$$

The functional $S_{n,\eta }$ can be expressed explicitly in terms of these different parameters as

108 $$\begin{aligned}&S_{n,\eta }\nonumber \\&\quad =\frac{n\beta ^2}{2}\left[ f(0)+\sum _{l=0}^k (m_l-m_{l+1})f\left( \int _\textbf{k}\left[ G_d(\textbf{k})-G_l(\textbf{k})\right] \right) -2n\eta f\left( \frac{1}{2}\int _\textbf{k}\,G_d(\textbf{k})\right) +n\eta f(0)\right] \nonumber \\&\qquad +\frac{1}{2}\int _\textbf{k}\left[ \ln \left( n G_0(\textbf{k})+\sum _{l=0}^k m_{l+1}(G_{l+1}(\textbf{k})-G_{l}(\textbf{k}))\right) \right. \nonumber \\&\quad \left. +n\sum _{l=0}^{k}\left( \frac{1}{m_{l+1}}-\frac{1}{m_{l}}\right) \ln \left( \sum _{j=l}^k m_{j+1}(G_{j+1}(\textbf{k})-G_{j}(\textbf{k}))\right) \right] \nonumber \\&\qquad -\frac{n\beta }{2}\int _\textbf{k}\nu (\textbf{k})G_{d}(\textbf{k})+c_n\;. \end{aligned}$$

Instead of keeping track of all the individual parameters, it will be convenient to rewrite the functional $S_{n,\eta }$ in terms of the Parisi overlap function

109 $$\begin{aligned} \tilde{g}(\textbf{k},x)=G_d(\textbf{k})-G_0(\textbf{k})-\sum _{l=1}^{k+1}(G_{l}(\textbf{k})-G_{l-1}(\textbf{k}))\Theta (x-m_l)\;, \end{aligned}$$

and the associated Parisi eigenvalue function

110 $$\begin{aligned} \tilde{\lambda }(\textbf{k},x)=(1-m_k)\tilde{g}(\textbf{k},m_k)+x\,\tilde{g}(\textbf{k},x)+\int _{x}^{m_k}dy\,\tilde{g}(\textbf{k},y)\;. \end{aligned}$$

For any fixed value of $\textbf{k}$, the function $\tilde{g}(\textbf{k},x)$ is a piece-wise constant non-decreasing function of *x* while $\tilde{\lambda }(\textbf{k},x)$ is a piece-wise constant non-increasing function. The functional $S_{n,\eta }$ can now be rewritten solely in terms of the function $\tilde{g}(\textbf{k},x)$, the parameter $m_k$ and the smallest overlap function $G_0(\textbf{k})$ as follows

111 $$\begin{aligned} S_{n,\eta }=&-\frac{n\beta }{2}\int _\textbf{k}\nu (\textbf{k})\left[ \tilde{g}(\textbf{k},n)+G_0(\textbf{k})\right] +\frac{\eta n^2\beta ^2}{2}\left[ f(0)-2 f\left( \frac{1}{2}\int _\textbf{k}\,\left[ \tilde{g}(\textbf{k},n)+G_0(\textbf{k})\right] \right) \right] \nonumber \\&+\frac{n\beta ^2}{2}\left[ f(0)+(m_k-1)\,f\left( \int _\textbf{k}\tilde{g}(\textbf{k},m_k)\right) -\int _{n}^{m_k}dx\,f\left( \int _\textbf{k}\tilde{g}(\textbf{k},x)\right) \right] \nonumber \\&+\frac{1}{2}\int _\textbf{k}\left[ -\frac{n}{m_k}\ln \tilde{\lambda }(\textbf{k},m_k)+\ln \left( \tilde{\lambda }(\textbf{k},n)+n G_0(\textbf{k})\right) -n\int _{n}^{m_k}\frac{dx}{x^2}\ln \tilde{\lambda }(\textbf{k},x)\right] +c_n\;.\nonumber \\ \end{aligned}$$

In the next section, we consider how to obtain the expression of this functional in the scaling limit $\beta \rightarrow \infty$ with $n=s/\beta$ and $s=O(1)$.

### Zero-Temperature Limit

To obtain the expression of the cumulant generating function of the ground-state energy, we need to take the specific scaling limit $n\rightarrow 0$ and $\beta \rightarrow \infty$ with fixed $s=n\beta =O(1)$. In this scaling limit, we expect the other parameters to take the following scaling forms

112 $$\begin{aligned}&v(\textbf{k})=\beta (G_d(\textbf{k})-G_k(\textbf{k}))=O(1)\;,\;\;w_M=\beta m_k=O(1)\;,\end{aligned}$$

113 $$\begin{aligned}&g(\textbf{k},z)=\tilde{g}\left( \textbf{k},\frac{z}{\beta }\right) \;,\;\;\lambda (\textbf{k},z)=\beta \tilde{\lambda }\left( \textbf{k},\frac{z}{\beta }\right) =v(\textbf{k})+z\,g(\textbf{k},z)+\int _{z}^{w_M}dw\,g(\textbf{k},w)\;, \end{aligned}$$

where the function $g(\textbf{k},w\ge w_M)=0$. Introducing these scaling forms and taking the scaling limit in the expression of $S_n$, one obtains the following expression

114 $$\begin{aligned} \mathcal{S}_{\eta ,L}(\mu )=&-\frac{s}{2}\int _\textbf{k}\nu (\textbf{k})\left[ g(\textbf{k},s)+G_0(\textbf{k})\right] +\frac{\eta s^2}{2}\left[ f(0)-2f\left( \frac{1}{2}\int _\textbf{k}\,\left[ g(\textbf{k},s)+G_0(\textbf{k})\right] \right) \right] \nonumber \\&+\frac{s}{2}\left[ -f'(0)\int _\textbf{k}v(\textbf{k})+w_M\,f\left( 0\right) -\int _{s}^{w_M}dz\,f\left( \int _\textbf{k}g(\textbf{k},z)\right) \right] \nonumber \\&+\frac{1}{2}\int _\textbf{k}\left[ -\frac{s}{w_M}\ln v(\textbf{k})+\ln \left( \lambda (\textbf{k},s)+s G_0(\textbf{k})\right) -s\int _{s}^{w_M}\frac{dz}{z^2}\ln \lambda (\textbf{k},z)\right] \;. \end{aligned}$$

The cumulant generating function $\varphi _L(s,\mu )$ of the ground-state energy can now be expressed in terms of a functional optimisation of $\mathcal{S}_{0,L}(\mu )$,

115 $$\begin{aligned} \varphi _L(s,\mu )={\left\{ \begin{array}{ll} \displaystyle \max _{g(\textbf{k},z),v(\textbf{k}),w_M,G_0(\textbf{k})}\mathcal{S}_{0,L}(\mu )\;,\;\;s<0\;, \\ \displaystyle \min _{g(\textbf{k},z),v(\textbf{k}),w_M,G_0(\textbf{k})}\mathcal{S}_{0,L}(\mu )\;,\;\;s\ge 0\;. \end{array}\right. } \end{aligned}$$

The cumulant generating function $\phi _L(s,\mu )$ of the ground-state energy difference is given by a similar expression, replacing $\mathcal{S}_{0,L}(\mu )\rightarrow \mathcal{S}_{1,L}(\mu )$. The average ground-state energy (difference) can be extracted from this expression and is again obtained from a functional optimisation problem as

116 $$\begin{aligned} e_{L,\mathrm typ}(\mu )=\lim _{N\rightarrow \infty }\left\langle e_{\min } \right\rangle =\lim _{N\rightarrow \infty }\left\langle \Delta e_{\min } \right\rangle =-\partial _s\varphi _L(0,\mu )=\max _{g(\textbf{k},z),v(\textbf{k}),w_M,G_0(\textbf{k})} \mathcal{S}_{L,\mathrm typ}(\mu )\;, \end{aligned}$$

where the functional $\mathcal{S}_{L,\mathrm typ}(\mu )$ to optimise reads

117 $$\begin{aligned}&\mathcal{S}_{L,\mathrm typ}(\mu ) =\frac{1}{2}\int _\textbf{k}\nu (\textbf{k})\left[ g(\textbf{k},0)+G_0(\textbf{k})\right] +\frac{1}{2}\left[ f'(0)\int _\textbf{k}v(\textbf{k})-w_M\,f\left( 0\right) +\int _{0}^{w_M}dz\,f\left( \int _\textbf{k}g(\textbf{k},z)\right) \right] \nonumber \\&+\frac{1}{2}\int _\textbf{k} \left[ \frac{1}{w_M}\ln \frac{v(\textbf{k})}{\lambda (\textbf{k},0)}-\frac{G_0(\textbf{k})}{\lambda (\textbf{k},0)}+\int _{0}^{w_M}\frac{dz}{z^2}\ln \frac{\lambda (\textbf{k},z)}{\lambda (\textbf{k},0)}\right] \;. \end{aligned}$$

An explicit expression for the rescaled variance can similarly be obtained as

118 $$\begin{aligned} \mathcal{V}_L(\mu )&=\lim _{N\rightarrow \infty }N L^d \left\langle \left( e_{\min }-\eta e_\textrm{flat}-\left\langle e_{\min } \right\rangle \right) ^2 \right\rangle \nonumber \\&=f\left( \int _\textbf{k}g_\textrm{typ}(\textbf{k},0)\right) +\eta f(0)-2\eta f\left( \frac{1}{2}\int _\textbf{k}\,\left[ g_\textrm{typ}(\textbf{k},0)+G_\textrm{typ}(\textbf{k})\right] \right) \nonumber \\&\qquad -\frac{1}{2}\int _\textbf{k}\left( \frac{G_\textrm{typ}(\textbf{k})}{\lambda _\textrm{typ}(\textbf{k},0)}\right) ^2\;, \end{aligned}$$

where $G_\textrm{typ}(\textbf{k})$, $g_\textrm{typ}(\textbf{k},z)$ and $\lambda _\textrm{typ}(\textbf{k},z)=v_\textrm{typ}(\textbf{k})+z\,g_\textrm{typ}(\textbf{k},z)+\int _{z}^{w_M}dw\,g_\textrm{typ}(\textbf{k},w)$ are the optimal functions obtained from the optimisation problem in Eq. (116). Optimising explicitly the expression of $\mathcal{S}_{L,\mathrm typ}(\mu )$ in Eq. (117) with respect to $G_0(\textbf{k})$ and $g(\textbf{k},z)$ provides explicit expressions for $G_\textrm{typ}(\textbf{k})$ and $\lambda _\textrm{typ}(\textbf{k},0)$,

119 $$\begin{aligned}&\lambda _\textrm{typ}(\textbf{k},0)=\frac{1}{\nu (\textbf{k})}\;,\;\;G_\textrm{typ}(\textbf{k})=-\lambda _\textrm{typ}(\textbf{k},0)^2 f'\left( Q_\textrm{typ}\right) =-\frac{f'\left( Q_\textrm{typ}\right) }{\nu (\textbf{k})^2}\;, \nonumber \\&\quad \textrm{where}\;\;Q_\textrm{typ}=\int _\textbf{k} g_\textrm{typ}(\textbf{k},0)\;. \end{aligned}$$

Inserting these expressions into the rescaled variance, the rescaled variance reads

120 $$\begin{aligned} \mathcal{V}_L(\mu )=&\lim _{N\rightarrow \infty }N L^d \left\langle \left( e_{\min }-\eta e_\textrm{flat}-\left\langle e_{\min } \right\rangle \right) ^2 \right\rangle \nonumber \\ =&f\left( Q_\textrm{typ}\right) +\eta f(0)-2\eta f\left( \frac{Q_\textrm{typ}-I_2(\mu ) f'\left( Q_\textrm{typ}\right) }{2}\right) -\frac{I_2(\mu ) f'\left( Q_\textrm{typ}\right) ^2}{2}\;, \end{aligned}$$

thus depending solely on $Q_\textrm{typ}$ defined above and on the integrals $I_2(x)$ defined in Eq. (15). The expression for $e_{L,\mathrm typ}(\mu )$ within the RS ansatz is simply obtained by taking the limit $w_M\rightarrow 0$ in Eq. (117) where $\lambda (\textbf{k},0)=v(\textbf{k})$ and $g(\textbf{k},0)=0$ and optimising over the remaining parameters $v(\textbf{k})$ and $G_0(\textbf{k})$, taking respectively the values $v_\textrm{typ}(\textbf{k})=1/\nu (\textbf{k})$ and $G_\textrm{typ}(\textbf{k})=-f'(0)/\nu (\textbf{k})^2$.

### Interpretation of the Optimal Fluctuations

Let us briefly discuss the probabilistic meaning of these parameters in the case where $s\ge 0$. To this end, we define the cumulative distribution $x_{\beta }(q)$ of overlaps between replicas

121 $$\begin{aligned} q_{ab}=\int _\textbf{k}\frac{\textbf{u}_a(\textbf{k})\cdot \textbf{u}_b(-\textbf{k})}{N}\;, \end{aligned}$$

at finite inverse temperature $\beta$. This function is a non-decreasing function of *q* with the boundary conditions $x_{\beta }(0\le q\le q_0)=n$, with $q_0$ the minimal overlap and $0<n<1$, while $x_{\beta }(q\ge q_d)=1$ with $q_d=q_{aa}$ the maximal (self)-overlap. The boundary condition can be interpreted as imposing an atom at $q=0$ with probability weight *n*. If the optimal solution corresponds to a finite number *k* of replica-symmetry breaking, the function $x_{\beta }(q)$ displays a finite number $k+1$ of steps. One may then associate an overlap function to this cumulative distribution function which reads

122 $$\begin{aligned} q_{\beta }(x)=q_0(\beta )\Theta (x-n)+\sum _{l=1}^{k+1}(q_{l}(\beta )-q_{l-1}(\beta ))\Theta (x-x_l(\beta ))\;, \end{aligned}$$

where $x_l(\beta )=\textrm{Prob}.\left[ q\le q_l(\beta )\right]$ for $l\ge 1$ and $x_0(\beta )=n$. If the solution is full replica-symmetry broken instead, the cumulative distribution function $x_{\beta }(q)$ is a continuous increasing function of $q\in [q_0(\beta ),q_d(\beta )]$ and we define $q_{\beta }(x)=x_{\beta }^{-1}(x)$ as its functional inverse defined over the interval $x\in [n,1]$ while $q_{\beta }(x)=0$ for $x\in [0,n)$. In the limit of zero-temperature, where $\beta \rightarrow \infty$ and $n\rightarrow 0$ with fixed $s=n\beta =O(1)$ on which we concentrate in this manuscript, the probability distribution function of the overlap $x_{\beta }'(q)$ localises in a vicinity of order $1/\beta$ around the self-overlap $q_d(\beta )$, yielding the following scaling form for the overlap function

123 $$\begin{aligned} q_d(\beta )-q_{\beta }(x)\approx Q(\beta x)+\frac{v}{\beta }\Theta (x-1)\;,\;\;\beta \gg 1\;, \end{aligned}$$

with $q_d=\displaystyle \lim _{\beta \rightarrow \infty }q_d(\beta )$ and where the parameters defined in that equation are related to the optimisation functions as

124 $$\begin{aligned} Q(z)=\lim _{\beta \rightarrow \infty }\left[ q_{d}(\beta )-q_{\beta }\left( \frac{z}{\beta }\right) \right] =\int _\textbf{k}g(\textbf{k},z)\;,\;\;v=\lim _{\beta \rightarrow \infty }\beta (q_d(\beta )-q_{k}(\beta ))=\int _\textbf{k}v(\textbf{k})\;, \end{aligned}$$

with the remaining optimisation parameters satisfying

125 $$\begin{aligned} w_M=\lim _{\beta \rightarrow \infty }\beta x_{k}(\beta )\;,\;\;q_0=\lim _{\beta \rightarrow \infty }q_0(\beta )=\int _\textbf{k}G_0(\textbf{k})\;. \end{aligned}$$

In particular, these identities yield $Q(z\ge w_M)=0$ and $Q(s)=q_d-q_0$. In the next section, we analyse the expression of the cumulant generating function and large deviation function in the different patterns of replica-symmetry.

## Analysis of the Different Phases

Let us now consider in more detail the expression of the cumulant generating function, large deviation function and variance in the different phase of replica-symmetry for short-range disorder.

### Replica Symmetric Phase

In the replica symmetric (RS) phase, the parameter $w_M=s$ or equivalently $g(\textbf{k},z\ge s)=0$. The functional $\mathcal{S}_{0,L}(\mu )$ to optimise simplifies drastically and takes the simpler RS form

126 $$\begin{aligned} \mathcal{S}_\textrm{RS}[G_0,v]=&-\frac{s}{2}\int _\textbf{k}\nu (\textbf{k})G_0(\textbf{k}) +\frac{s}{2}\left[ -f'(0)\int _\textbf{k}v(\textbf{k})+s\,f\left( 0\right) \right] +\frac{1}{2}\int _\textbf{k}\ln \left( 1+\frac{s G_0(\textbf{k})}{v(\textbf{k})}\right) \;, \end{aligned}$$

which now depends only on the two functional parameters $G_0(\textbf{k})$ and $v(\textbf{k})$. Computing the functional derivative of $\mathcal{S}_\textrm{RS}$ with respect to $G_0(\textbf{k})$ and $v(\textbf{k})$, one obtains explicit equations satisfied by their optimal values $G_\textrm{RS}(\textbf{k})$ and $v_\textrm{RS}(\textbf{k})$, namely

127 $$\begin{aligned} \frac{\delta \mathcal{S}_\textrm{RS}}{\delta G_0(\textbf{k})}[G_\textrm{RS},v_\textrm{RS}]&=0=-\frac{s}{2}\nu (\textbf{k})+\frac{s}{2(v_\textrm{RS}(\textbf{k})+s G_\textrm{RS}(\textbf{k}))}\;,\end{aligned}$$

128 $$\begin{aligned} \frac{\delta \mathcal{S}_\textrm{RS}}{\delta v(\textbf{k})}[G_\textrm{RS},v_\textrm{RS}]&=0=-\frac{s}{2}f'(0)+\frac{1}{2}\left[ \frac{1}{v_\textrm{RS}(\textbf{k})+s G_\textrm{RS}(\textbf{k})}-\frac{1}{v_\textrm{RS}(\textbf{k})}\right] \;. \end{aligned}$$

The solution to these equations reads

129 $$\begin{aligned} v_\textrm{RS}(\textbf{k})=\frac{1}{\nu (\textbf{k})-sf'(0)}\;,\;\;G_\textrm{RS}(\textbf{k})=-\frac{f'(0)}{\nu (\textbf{k})(\nu (\textbf{k})-sf'(0))}\;. \end{aligned}$$

Note that the parameter $v_\textrm{RS}$ must be positive, which is only possible if

130 $$\begin{aligned} s>\frac{\min _\textbf{k}\nu (\textbf{k})}{f'(0)}=\frac{\mu }{f'(0)}\;, \end{aligned}$$

where we remind that $f'(0)<0$. This bond indicates that the replica-symmetric ansatz to the CGF cannot describe the function for all $s\in \mathbb {R}$.

Inserting the optimal values of $G_\textrm{RS}(\textbf{k})$ and $v_\textrm{RS}(\textbf{k})$ in the functional $\mathcal{S}_\textrm{RS}$, the RS expression for the CGF reads

131 $$\begin{aligned} \varphi _{L,\mathrm RS}(s,\mu )=&\mathcal{S}_\textrm{RS}[G_\textrm{RS},v_\textrm{RS}] =\frac{s^2}{2}f(0)+\frac{1}{2}\int _\textbf{k}\ln \left( 1-\frac{s f'(0)}{\nu (\textbf{k})}\right) \;. \end{aligned}$$

Let us define for fixed *s* the centred energy $e_{L,\mathrm RS}(s,\mu )$ as

$$\begin{aligned} e_{L,\mathrm RS}(s,\mu )=-\partial _s\varphi _{L,\mathrm RS}(s,\mu )=&-s f\left( 0\right) +\frac{f'(0)}{2}\int _\textbf{k}\frac{1}{\nu (\textbf{k})-sf'(0)}\;. \end{aligned}$$

From this expression, one may easily check that

132 $$\begin{aligned} e_{L,\mathrm RS}(\mu )=e_{L,\mathrm RS}(s=0,\mu )=-\partial _s\varphi _{L,\mathrm RS}(s,\mu )=\frac{f'(0)}{2}\int _\textbf{k}\frac{1}{\nu (\textbf{k})}=\frac{f'(0)}{2}I_1(\mu )\;, \end{aligned}$$

where the function $I_1(x)$ is defined in Eq. (15). To ensure that each energy corresponds to a single value of *s*, one must ensure that the CGF is convex, i.e. that its second derivative

133 $$\begin{aligned}&\partial _s^2\varphi _{L,\mathrm RS}(s,\mu )=-\partial _s e_{L,\mathrm RS}(s,\mu ) \nonumber \\&\quad =f(0)-\frac{1}{2}\int _\textbf{k}\left( \frac{f'(0)}{\nu (\textbf{k})-s f'(0)}\right) ^2=f(0)-\frac{f'(0)^2}{2}I_2(\mu -s f'(0))\;, \end{aligned}$$

is positive for any *s* where the CGF is given by its RS ansatz. Thus, one must have throughout the RS phase that $I_2(\mu -s f'(0))\le f(0)/(2f'(0)^2)$. The rescaled variance is obtained within the RS ansatz by taking $s=0$ in the expression above

134 $$\begin{aligned} \mathcal{V}_\textrm{RS}(\mu )=\partial _s^2\varphi _{L,\mathrm RS}(0,\mu )=f(0)-\frac{I_2(\mu ) f'(0)^2}{2}\;. \end{aligned}$$

Finally, in the range where the CGF is convex, the associated large deviation function can be expressed parametrically in terms of *s* as

135 $$\begin{aligned}&\Lambda _{L,\mathrm RS}(e,\mu )=s\partial _s\varphi _{L,\mathrm RS}(s,\mu )-\varphi _{L,\mathrm RS}(s,\mu ) \nonumber \\&\quad =\frac{s^2}{2}f\left( 0\right) -\frac{1}{2}\int _\textbf{k}\left[ \frac{s f'(0)}{\nu (\textbf{k})-s f'(0)}+\ln \left( 1-\frac{s f'(0)}{\nu (\textbf{k})}\right) \right] \;,\end{aligned}$$

136 $$\begin{aligned} \textrm{where}\;\;e=&-s f(0)+\frac{f'(0)}{2}\int _\textbf{k}\frac{1}{\nu (\textbf{k})-s f'(0)}\;. \end{aligned}$$

Both the criterion of positivity of $v_\textrm{RS}(\textbf{k})$ and of convexity of the CGF indicate that the RS ansatz cannot describe the full range of values of $s\in \mathbb {R}$. In the next section, we will see the correct critetion ensuring the stability of the RS ansatz.

### Replica Symmetry Breaking

The replica symmetric solution to the cumulant generating function can be shown to be unstable for any $s<s_\textrm{AT}$ where $s_\textrm{AT}$ is the solution of

137 $$\begin{aligned} f''(0)\int _\textbf{k}&\frac{1}{(\nu (\textbf{k})-s_\textrm{AT} f'(0))^2}=f''(0) I_2\left( \mu -s_\textrm{AT} f'(0)\right) =1\;. \end{aligned}$$

A similar criterion appears in the definition of the Larkin mass $\mu _c$ such that the typical energy is RS for $\mu >\mu _c$ and RSB for $\mu <\mu _c$ [3, 14]. In particular $\mu _c$ is defined as the solution of $f''(0) I_2\left( \mu _c\right) =1$. Comparing with the expression above, it is simple to conclude that

138 $$\begin{aligned} s_\textrm{AT}=\frac{\mu -\mu _c}{f'(0)}\;. \end{aligned}$$

Note that $s_\textrm{AT}$ only provides a criterion for continuous transitions but discontinuous transitions may occur as well. These transitions will be considered below in the section on the one-step replica symmetry breaking. Using the definition of $s_\textrm{AT}$ in Eq. (137) as well as the expression in Eq. (133), and one can show that

139 $$\begin{aligned} \partial _s^2\varphi _{L,\mathrm RS}(s_\textrm{AT},\mu )=f\left( 0\right) -\frac{f'(0)^2}{2}\int _\textbf{k}\frac{1}{(\nu (\textbf{k})-s_\textrm{AT} f'(0))^2}=f\left( 0\right) -\frac{f'(0)^2}{2f''(0)}>0\;, \end{aligned}$$

indicating that the CGF is convex for any $s\ge s_\textrm{AT}$. The identity in Eq. (139) is easily proven using the identity $f(q)=\int _0^{\infty }dk e^{-q k^2} \tilde{f}(k)$ with $\tilde{f}(k)\ge 0$ by the chain of inequalities

140 $$\begin{aligned}&f''(0)f(0)-\frac{f'(0)^2}{2}=\int _0^{\infty }\int _0^{\infty } dk_1 dk_2 \tilde{f}(k_1)\tilde{f}(k_2)\left( k_1^4+k_2^4-k_1^2 k_2^2\right) \nonumber \\&\quad >\int _0^{\infty }\int _0^{\infty } dk_1 dk_2 \tilde{f}(k_1)\tilde{f}(k_2)\left( k_1^4+k_2^4-2k_1^2 k_2^2\right) \nonumber \\&\qquad =\int _0^{\infty }\int _0^{\infty } dk_1 dk_2 \tilde{f}(k_1)\tilde{f}(k_2)\left( k_1^2-k_2^2\right) ^2\ge 0\;. \end{aligned}$$

We have now identified one explicit condition of instability of the RS solution. Let us now analyse the expression of the cumulant generating function and large deviation function when the replica-symmetry is broken.

### One-Step Replica Symmetry Broken Solution

Let us now consider the 1RSB solution. The expression for the functional to optimise reads in that phase

141 $$\begin{aligned} \mathcal{S}_\textrm{1RSB}=&-\frac{s}{2}\left[ \int _\textbf{k}\nu (\textbf{k}) (G(\textbf{k})+G_0(\textbf{k}))\right] +\frac{s}{2}\left[ s f\left( Q\right) -v f'(0)+w\left( f(0)-f\left( Q\right) \right) \right] \end{aligned}$$

142 $$\begin{aligned}&+\frac{1}{2}\int _\textbf{k}\left[ \ln \left( 1+\frac{s G_0(\textbf{k})}{v(\textbf{k})+w G(\textbf{k})}\right) -\frac{s}{w}\ln \left( \frac{v(\textbf{k})}{v(\textbf{k})+w G(\textbf{k})}\right) \right] \;, \end{aligned}$$

where $$v=\int _\textbf{k}v(\textbf{k})$$ and $$Q=\int _\textbf{k}G(\textbf{k})$$.

The optimisation equations of $\mathcal{S}_\textrm{1RSB}$ with respect to the parameters $G(\textbf{k}),G_0(\textbf{k}),v(\textbf{k}),w$ respectively read

143 $$\begin{aligned} \frac{\delta \mathcal{S}_\textrm{1RSB}}{\delta G(\textbf{k})}=&-\frac{s}{2}\nu (\textbf{k})+\frac{s}{2}(s-w)f'(Q)+\frac{1}{2}\left[ \frac{s-w}{v(\textbf{k})+w G(\textbf{k})}+\frac{w}{v(\textbf{k})+w G(\textbf{k})+s G_0(\textbf{k})}\right] =0\;, \end{aligned}$$

144 $$\begin{aligned} \frac{\delta \mathcal{S}_\textrm{1RSB}}{\delta G_0(\textbf{k})}=&-\frac{s}{2}\nu (\textbf{k})+\frac{s}{2(v(\textbf{k})+w G(\textbf{k})+s G_0(\textbf{k}))}=0\;,\end{aligned}$$

145 $$\begin{aligned} \frac{\delta \mathcal{S}_\textrm{1RSB}}{\delta v(\textbf{k})}=&-\frac{s}{2}f'(0)-\frac{s}{2}\left[ \frac{G_0(\textbf{k})}{(v(\textbf{k})+w G(\textbf{k}))(v(\textbf{k})+w G(\textbf{k})+s G_0(\textbf{k}))}+\frac{G(\textbf{k})}{v(\textbf{k})(v(\textbf{k})+w G(\textbf{k}))}\right] =0\;,\end{aligned}$$

146 $$\begin{aligned} \frac{\partial \mathcal{S}_\textrm{1RSB}}{\partial w}=&\frac{1}{2}\int _\textbf{k}\left[ \frac{G(\textbf{k})}{w}\left( \frac{s-w}{v(\textbf{k})+w G(\textbf{k})}+\frac{w}{v(\textbf{k})+w G(\textbf{k})+s G_0(\textbf{k})}\right) +\frac{s}{w^2}\ln \left( \frac{v(\textbf{k})}{v(\textbf{k})+w G(\textbf{k})}\right) \right] \nonumber \\&+\frac{s}{2}\left( f(0)-f(Q)\right) =0\;. \end{aligned}$$

Solving the first three equations, explicit expressions for $v(\textbf{k})$, $G_0(\textbf{k})$ and $G(\textbf{k})$ can be found in terms of *Q* and *w*. They read

147 $$\begin{aligned} G_0(\textbf{k})&=\frac{1}{s}\left[ \frac{1}{\nu (\textbf{k})}-\frac{1}{\nu (\textbf{k})-s f'(Q)}\right] \; ,\end{aligned}$$

148 $$\begin{aligned} G(\textbf{k})&=\frac{1}{w}\left[ \frac{1}{\nu (\textbf{k})-s f'(Q)}-\frac{1}{\nu (\textbf{k})-s f'(Q)+\Sigma }\right] \;,\end{aligned}$$

149 $$\begin{aligned} v(\textbf{k})&= \frac{1}{\nu (\textbf{k})-s f'(Q)+\Sigma }\;,\;\;\textrm{where}\;\;\Sigma =w(f'(Q)-f'(0))\;. \end{aligned}$$

The remaining equation can be re-expressed using the identities above as

150 $$\begin{aligned}&f(0)-f(Q)+Q f'(Q)\nonumber \\&\quad =\frac{1}{w^2}\int _\textbf{k}\left[ \ln \left( 1+\frac{w(f'(Q)-f'(0))}{\nu (\textbf{k})-s f'(Q)}\right) -\frac{w(f'(Q)-f'(0))}{\nu (\textbf{k})-s f'(Q)+w(f'(Q)-f'(0))}\right] \;. \end{aligned}$$

Finally, using the definition of *Q* and $q_0$, one obtains two additional self-consistent integral equations

151 $$\begin{aligned} Q(s)\equiv Q&=\frac{1}{w}\int _\textbf{k}\left[ \frac{1}{\nu (\textbf{k})-s f'(Q)}-\frac{1}{\nu (\textbf{k})-s f'(Q)+w(f'(Q)-f'(0))}\right] \;. \end{aligned}$$

The CGF can now be expressed solely in terms of the parameters *s*, *Q* and *w* as

152 $$\begin{aligned} \varphi _{L,\mathrm 1RSB}(s,\mu ) =&\frac{s}{2}\left[ s f\left( 0\right) +(w-s)\left( f(0)-f\left( Q\right) +Q f'(Q)\right) \right] \nonumber \\&+\frac{1}{2}\int _\textbf{k}\left[ \ln \left( 1-\frac{s f'(Q)}{\nu (\textbf{k})}\right) +\frac{s}{w}\ln \left( 1+\frac{w(f'(Q)-f'(0))}{\nu (\textbf{k})-s f'(Q)}\right) \right] \;. \end{aligned}$$

From the expression above, one can obtain the average ground-state energy difference as

153 $$\begin{aligned}&e_{L,\mathrm 1RSB}(\mu )=-\partial _s\varphi _{L,\mathrm 1RSB}(0,\mu ) \nonumber \\&\quad =e_{L,\mathrm RS}(\mu )+w_\textrm{typ}\left( f(Q_\textrm{typ})-f(0)-\frac{Q_\textrm{typ}}{2} \left[ f'(Q_\textrm{typ})+f'(0)\right] \right) \;, \end{aligned}$$

where $Q_\textrm{typ}=Q(s=0)$ and $w_\textrm{typ}=w(s=0)$ are the optimal value of *Q* and *w* for $s=0$, satisfying

154 $$\begin{aligned} Q_\textrm{typ}&=\int _\textbf{k}\frac{1}{w_\textrm{typ}}\left( \frac{1}{\nu (\textbf{k})}-\frac{1}{\nu (\textbf{k})+w_\textrm{typ}(f'\left( Q_\textrm{typ}\right) -f'(0))}\right) \;,\end{aligned}$$

155 $$\begin{aligned}&f\left( Q_\textrm{typ}\right) -f(0)-Q_\textrm{typ} f'\left( Q_\textrm{typ}\right) \nonumber \\&=\frac{1}{w_\textrm{typ}^2}\int _\textbf{k}\left[ \frac{w_\textrm{typ}(f'\left( Q_\textrm{typ}\right) -f'(0))}{\nu (\textbf{k})+w_\textrm{typ}(f'\left( Q_\textrm{typ}\right) -f'(0))}-\ln \left( 1+\frac{w_\textrm{typ}(f'\left( Q_\textrm{typ}\right) -f'(0))}{\nu (\textbf{k})}\right) \right] \;. \end{aligned}$$

The expression for the rescaled variance can be derived from Eq. (152) as

156 $$\begin{aligned} \mathcal{V}_{L,\mathrm 1RSB}(\mu )=\partial _s^2\varphi _{L,\mathrm 1RSB}(0,\mu )=f\left( Q_\textrm{typ}\right) -\frac{I_2(\mu ) f'\left( Q_\textrm{typ}\right) ^2}{2}\;. \end{aligned}$$

The transition from the RS to the 1RSB phase in the variance is always continuous and occurs at the Larkin mass $\mu _{c}$ satisfying $I_2(\mu _{c})=1/f''(0)$. Expanding Eqs. (154-155) in the vicinity of $\mu _{c}$ one obtains at leading order $w_\textrm{typ}\approx -f^{(3)}(0)/(2f''(0)^3 I_3(\mu _{c}))$ and $Q_\textrm{typ}\approx -(\mu -\mu _{c})/(w_\textrm{typ}f''(0))$, which yields a second order transition in the variance

157 $$\begin{aligned} \mathcal{V}_{L,\mathrm 1RSB}(\mu )-\mathcal{V}_{L,\mathrm RS}(\mu )=2\frac{I_3(\mu _{c})^2 f''(0)^3}{f^{(3)}(0)}f'(0)(\mu -\mu _{c})^2+O(\mu -\mu _{c})^3\;. \end{aligned}$$

Let us investigate transitions between the RS and 1RSB phases. There are two types of transitions that may occur, i.e. continuous transitions as $Q\rightarrow 0$ and discontinuous transitions as $w\rightarrow s$. These transitions can be investigated by considering the expansion of

158 $$\begin{aligned}&\varphi _{L,\mathrm 1RSB}(s,\mu )-\varphi _{L,\mathrm RS}(s,\mu )\nonumber \\&\quad =\frac{1}{2}\int _\textbf{k} \left[ \ln \frac{\nu (\textbf{k})-s f'(Q)}{\nu (\textbf{k})-s f'(0)}+\frac{s}{w}\left( \frac{2w-s}{w}\ln \left( 1+\frac{w(f'(Q)-f'(0))}{\nu (\textbf{k})-s f'(Q)}\right) \right. \right. \nonumber \\&\qquad \left. \left. +\frac{s-w}{w}\left[ 1-\frac{1}{1+\frac{w(f'(Q)-f'(0))}{\nu (\textbf{k})-s f'(Q)}}\right] \right) \right] \;, \end{aligned}$$

in terms of these parameters. It is simple to check that this expression vanishes as the *s* dependent parameters $Q\rightarrow 0$ or $m\rightarrow s$. In order to obtain the location of these transitions, one may expand the self-consistent equations Eqs. (151) and (150) around these solutions. Expanding Eq. (151) up to first order in *Q* or Eq. (150) up to second order both yield the same equation as (137) for the location of the continuous transition, which occurs at $s=s_\textrm{AT}$. At the transition, the parameter *w* takes the value

159 $$\begin{aligned} w_\textrm{AT}\equiv w(s_\textrm{AT})=-\frac{I_2(\mu -s_\textrm{AT}f'(0))^3}{2I_3(\mu -s_\textrm{AT}f'(0))}f^{(3)}(0)=-\frac{f^{(3)}(0)}{2(f''(0))^3 I_3(\mu _c)}\;. \end{aligned}$$

Setting $w\rightarrow s$ in Eqs. (151) and (150) yields the two simpler equations for the parameters $s_\textrm{dis}$ and $Q_\textrm{dis}=Q(s_\textrm{dis})$, which read

160 $$\begin{aligned}&Q_\textrm{dis}=\frac{1}{s_\textrm{dis}}\int _\textbf{k}\left[ \frac{1}{\nu (\textbf{k})-s_\textrm{dis} f'(Q_\textrm{dis})}-\frac{1}{\nu (\textbf{k})-s_\textrm{dis} f'(0)}\right] \;,\end{aligned}$$

161 $$\begin{aligned}&f(0)-f(Q_\textrm{dis})+Q_\textrm{dis} f'(Q_\textrm{dis})=\frac{1}{s_\textrm{dis}^2}\int _\textbf{k}\left[ \ln \frac{\nu (\textbf{k})-s_\textrm{dis} f'(0)}{\nu (\textbf{k})-s_\textrm{dis} f'(Q_\textrm{dis})}-\frac{s_\textrm{dis}(f'(Q_\textrm{dis})-f'(0))}{\nu (\textbf{k})-s_\textrm{dis} f'(0)}\right] \;. \end{aligned}$$

Expanding these equations as $Q_\textrm{dis}\rightarrow 0$, one may again check that $s_\textrm{dis}\rightarrow s_\textrm{AT}$. The transition is expected to be discontinuous for sufficiently low values of $\mu <\mu _\textrm{dis}$ while it is continuous for $\mu >\mu _\textrm{dis}$. At the transition, one should have $s_\textrm{dis}=s_\textrm{AT}=w_\textrm{AT}$, yielding

162 $$\begin{aligned} \mu _\textrm{dis}=\mu _c+f'(0)w_\textrm{AT}=\mu _c-\frac{f'(0)f^{(3)}(0)}{2(f''(0))^3 I_3(\mu _c)}<\mu _c\;. \end{aligned}$$

One may now investigate the nature of the transition between the two branch of the CGF. For $\mu >\mu _\textrm{dis}$, the transition is continuous (in terms of *Q*) and occurs as $s\rightarrow s_\textrm{AT}$. The CGF displays a third order transition

163 $$\begin{aligned} \varphi _{L,\mathrm 1RSB}(s,\mu )-\varphi _{L,\mathrm RS}(s,\mu )&=I_3\left( \mu _c\right) \frac{s_\textrm{AT}f'(0)^3}{6(s_\textrm{AT}-w_\textrm{AT})}(s-s_\textrm{AT})^3+O(s-s_\textrm{AT})^4\nonumber \\&=I_3\left( \mu _c\right) \frac{\mu -\mu _c}{6(\mu -\mu _\textrm{dis})}f'(0)^3(s-s_\textrm{AT})^3+O(s-s_\textrm{AT})^4\;. \end{aligned}$$

For $\mu <\mu _\textrm{dis}$ instead, the transition is discontinuous (in terms of *Q*) and occurs as $s\rightarrow s_\textrm{dis}$. The transition in the CGF is of second order

164 $$\begin{aligned}&\varphi _{L,\mathrm 1RSB}(s,\mu )-\varphi _{L,\mathrm RS}(s,\mu ) \nonumber \\&=\frac{\partial _s^2\varphi _{L,\mathrm 1RSB}(s_\textrm{dis},\mu )-\partial _s^2\varphi _{L,\mathrm RS}(s_\textrm{dis},\mu )}{2}(s-s_\textrm{dis})^2+O(s-s_\textrm{dis})^3\nonumber \\&\textrm{where}\;\;\partial _s^2\varphi _{L,\mathrm RS}(s_\textrm{dis},\mu ) \nonumber \\&=2f(0)-f'(0)^2 I_2(\mu -s_\textrm{dis}f'(0))\;,\nonumber \\&\partial _s^2\varphi _{L,\mathrm 1RSB}(s_\textrm{dis},\mu )\nonumber \\&=\frac{2\left[ f(Q_\textrm{dis})f'(0)(f'(0)-2f'(Q_\textrm{dis}))+(f(0)+Q_\textrm{dis}f'(0))f'(Q_\textrm{dis})^2\right] \,I_2(\mu -s_\textrm{dis}f'(0))}{(f'(Q_\textrm{dis})-f'(0))^2\,I_2(\mu -s_\textrm{dis}f'(0))-2(f(0)-f(Q_\textrm{dis})+Q_\textrm{dis} f'(Q_\textrm{dis}))}\nonumber \\&-\frac{4f(Q_\textrm{dis})[f(0)-f(Q_\textrm{dis})+Q_\textrm{dis}f'(Q_\textrm{dis})]}{(f'(Q_\textrm{dis})-f'(0))^2\,I_2(\mu -s_\textrm{dis}f'(0))-2(f(0)-f(Q_\textrm{dis})+Q_\textrm{dis} f'(Q_\textrm{dis}))}\;. \end{aligned}$$

As $s_\textrm{dis}\rightarrow s_\textrm{AT}$, one has that $Q_\textrm{dis}\rightarrow 0$ and the coefficients in the second order expansion above match.

The convexity of the CGF $\varphi _{L,\mathrm 1RSB}(s,\mu )$ is not straightforward to check. In fact even making sure that the expression for the rescaled variance is positive within the 1RSB phase is hard. Supposing this convexity, the large deviation function associated to this expression for the cumulant generating function can be obtained parametrically in terms of *s* as

165 $$\begin{aligned} \Lambda _{L,\mathrm 1RSB}(e,\mu )&=s\partial _s\varphi _{L,\mathrm 1RSB}(s,\mu )-\varphi _{L,\mathrm 1RSB}(s,\mu )\nonumber \\&=\frac{s^2}{2}f\left( Q\right) -\frac{1}{2}\int _\textbf{k}\left[ \ln \left( 1-\frac{s f'(Q)}{\nu (\textbf{k})}\right) +\frac{s f'(Q)}{\nu (\textbf{k})-s f'(Q)}\right] \;,\end{aligned}$$

166 $$\begin{aligned} \textrm{where}\;\;e&=-w f(0)-(s-w)\left( f(Q)-\frac{Q f'(Q)}{2}\right) \nonumber \\&+\frac{f'(0)}{2}\int _\textbf{k}\frac{1}{\nu (\textbf{k})-s f'(Q)+w(f'(Q)-f'(0))}\;, \end{aligned}$$

where $Q\equiv Q(s)$ and $w\equiv w(s)$ are the solutions of Eqs. (150-151). The transitions of the CGF are reflected in the large deviation function with transition of the same order. In particular, the transition is of second order for $\mu <\mu _\textrm{dis}$ and at the transition

167 $$\begin{aligned}&\Lambda _{L,\mathrm 1RSB}(e,\mu )-\Lambda _{L,\mathrm RS}(e,\mu )=\left( \frac{1}{\partial _s^2\varphi _{L,\mathrm 1RSB}(s_\textrm{dis},\mu )}-\frac{1}{\partial _s^2\varphi _{L,\mathrm RS}(s_\textrm{dis},\mu )}\right) \frac{(e-e_\textrm{dis})^2}{2}\end{aligned}$$

168 $$\begin{aligned}&\quad +O(e-e_\textrm{dis})^3\;, \end{aligned}$$

where the value of energy at the transition reads

169 $$\begin{aligned} e_\textrm{dis}=-s_\textrm{dis}f(0)+\frac{f'(0)}{2}\int _\textbf{k}\frac{1}{\nu (\textbf{k})-s_\textrm{dis} f'(0)}\;. \end{aligned}$$

For $\mu >\mu _\textrm{dis}$, the large deviation function displays a third order transition

170 $$\begin{aligned} \Lambda _{L,\mathrm 1RSB}(e,\mu )-\Lambda _{L,\mathrm RS}(e,\mu )=I_3(\mu _c)\frac{f'(0)^3 s_\textrm{AT}}{6\mathcal{V}_L(\mu _c)^3(s_\textrm{AT}-z(0))}\left( e-e_\textrm{AT}\right) ^3+O(e-e_\textrm{AT})^4\;, \end{aligned}$$

where the value of energy at the transition reads

171 $$\begin{aligned} e_\textrm{AT}=-s_\textrm{AT}f(0)+\frac{f'(0)}{2}\int _\textbf{k}\frac{1}{\nu (\textbf{k})-s_\textrm{AT} f'(0)}=-(\mu -\mu _c)\frac{f(0)}{f'(0)}+\frac{f'(0)}{2}I_1(\mu _c)\;. \end{aligned}$$

Finally, let us analyse the behaviour of the cumulant generating function as $s\rightarrow -\infty$. For a short range correlation functions, using the identity $f(q)=\int _0^{\infty }dk\,e^{-q k^2}\,\tilde{f}(k)$ with $\tilde{f}(k)\ge 0$, one can check that $f'(q)$ is a negative decreasing function of *q* which satisfies $f'(q)\rightarrow 0$ as $q\rightarrow \infty$. We suppose additionally that $f'(q)$ only reaches zero asymptotically as $q\rightarrow \infty$ and $q f'(q)\rightarrow 0$ in that limit. A consistent solution for *Q* as $s\rightarrow -\infty$ is then provided by $Q\rightarrow \infty$ with fixed $s f'(Q)\rightarrow \min _\textbf{k}\nu (\textbf{k})=\mu$. This ensures that the first term in the integral in Eq. (151) diverges. Using our assumptions, the left-hand side of Eq. (150) reaches the finite value *f*(0) as $s\rightarrow -\infty$. It is thus natural to expect that *w* reaches a finite value $w_{\infty }$ in that limit. This asymptotic value must satisfy

172 $$\begin{aligned} f(0)=\frac{1}{w_{\infty }^2}\int _\textbf{k}\left[ \ln \left( 1-\frac{w_{\infty }f'(0)}{\nu (\textbf{k})-\mu }\right) +\frac{w_{\infty }f'(0)}{\nu (\textbf{k})-\mu -w_{\infty }f'(0)}\right] \;. \end{aligned}$$

Note that the case of the toy model ($d=0$) needs to be considered separately. Using these results, the energy $e_{L,\mathrm 1RSB}(s,\mu )$ in Eq. (166) behaves asymptotically as

173 $$\begin{aligned} e_{L,\mathrm 1RSB}(s,\mu )\sim s\frac{Q f'(Q)}{2}\sim \frac{\mu Q}{2}\,\;\;s\rightarrow -\infty \;. \end{aligned}$$

The large deviation function on the other hand behaves as

174 $$\begin{aligned} \Lambda _{L,\mathrm 1RSB}(e_{L,\mathrm 1RSB}(s,\mu ),\mu )\sim \frac{s^2}{2}f(Q)\sim \frac{\mu ^2 f(Q)}{2f'(Q)^2}\;,\,\;\;s\rightarrow -\infty \;. \end{aligned}$$

Thus, as $s\rightarrow -\infty$, one obtains that $\epsilon \rightarrow +\infty$ and the asymptotic behaviour of the large deviation function is independent of the dimension but depends explicitly on the correlation function with

175 $$\begin{aligned} \Lambda _{L,\mathrm 1RSB}(e,\mu )\approx \frac{\displaystyle \mu ^2 f\left( 2e/\mu \right) }{\displaystyle 2f'\left( 2e/\mu \right) ^2}\;,\,\;\;e\rightarrow +\infty \;. \end{aligned}$$

Note that, in stark distinction with the large deviation for the the spherical spin model [21, 22], the LDF with speed *N* for the elastic manifold is defined over the whole range of centred energy $e\in \mathbb {R}$ for any positive value of $\mu >0$. It is thus expected that for $\mu >0$ there is no large deviation regime with speed $N^2$. Now that we have treated in detail the case of one-step replica symmetry broken ansatz, we turn to the full replica symmetry pattern.

### Full Replica Symmetry Broken Solution

In the full replica symmetry broken (FRSB) solution, there is no simplification to the functional $\mathcal{S}_{0,L}(\mu )=\mathcal{S}_\textrm{FRSB}$ in Eq. (114). The optimal value of the different parameters ($g(\textbf{k},z),G_0(\textbf{k}),v(\textbf{k}),w_M$) is obtained by extremisation of the functional $\mathcal{S}_\textrm{FRSB}$. We compute below the (functional) derivatives with respect to these different parameters. Taking the derivative of $\mathcal{S}_\textrm{FRSB}$ with respect to the parameter $w_M$ and evaluating at the optimal solution yields

$$\begin{aligned} \frac{\partial \mathcal{S}_\textrm{FRSB}}{\partial w_M}=&\frac{s}{2}\left[ f\left( 0\right) -f\left( \int _\textbf{k}g(\textbf{k},w_M)\right) \right] +\frac{s}{2w_M^2}\int _\textbf{k}\ln \frac{v(\textbf{k})}{\lambda (\textbf{k},w_M)}=0\;, \end{aligned}$$

which using $\lambda (\textbf{k},z)=v(\textbf{k})+z\,g(\textbf{k},z)+\int _{z}^{w_M}dw\,g(\textbf{k},w)$ is simply enforced by ensuring the boundary condition $g(\textbf{k},w_M)=0$. The functional derivative with respect to $g(\textbf{k},z)$ evaluated at the optimal solution reads, for $z>s$,

176 $$\begin{aligned} \frac{\delta \mathcal{S}_\textrm{FRSB}}{\delta g(\textbf{k},z)}=&-\frac{s}{2}f'\left( \int _\textbf{k}g(\textbf{k},z)\right) +\frac{1}{2(\lambda (\textbf{k},s)+s G_0(\textbf{k}))}-\frac{s}{2}\int _{s}^{w_M}\frac{dw\left( \Theta (z-w)+z\delta (z-w)\right) }{w^2\,\lambda (\textbf{k},w)}\nonumber \\&=-\frac{s}{2}f'\left( \int _\textbf{k}g(\textbf{k},z)\right) -\frac{s}{2z\lambda (\textbf{k},z)}+\frac{1}{2(\lambda (\textbf{k},s)+s G_0(\textbf{k}))}-\frac{s}{2}\int _{s}^{z}\frac{dw}{w^2\,\lambda (\textbf{k},w)}\nonumber \\&=-\frac{s}{2}\left[ f'\left( \int _\textbf{k}g(\textbf{k},z)\right) +\frac{G_0(\textbf{k})}{\lambda (\textbf{k},s)(\lambda (\textbf{k},s)+s G_0(\textbf{k}))}-\int _{s}^{z}\frac{dw\,\partial _w g(\textbf{k},w)}{\lambda (\textbf{k},w)^2}\right] =0\;, \end{aligned}$$

where we used the identity $\partial _w\lambda (\textbf{k},w)=w\,\partial _w g(\textbf{k},w)$. Taking the functional derivative of $\mathcal{S}_\textrm{FRSB}$ with respect to $G_0(\textbf{k})$ and evaluating at the optimal solution yields

177 $$\begin{aligned} \frac{\delta \mathcal{S}_\textrm{FRSB}}{\delta G_0(\textbf{k})}=&-\frac{s}{2}\nu (\textbf{k})+\frac{s}{2(\lambda (\textbf{k},s)+s G_0(\textbf{k}))}=0\;. \end{aligned}$$

Using this equation as well as the limit $z\rightarrow s$ of Eq. (176), one can obtain the following explicit expressions

178 $$\begin{aligned} \lambda (\textbf{k},s)&=\frac{1}{\nu (\textbf{k})-s f'\left( Q(s)\right) }\;,\end{aligned}$$

179 $$\begin{aligned} G_0(\textbf{k})&=\frac{1}{s}\left[ \frac{1}{\nu (\textbf{k})}-\frac{1}{\nu (\textbf{k})-s f'\left( Q(s)\right) }\right] \;, \end{aligned}$$

where we have defined $Q(z)=\int _\textbf{k}g(\textbf{k},z)$.

Taking the functional derivative of $\mathcal{S}_\textrm{FRSB}$ with respect to $v(\textbf{k})$ and evaluating at the optimal solution yields

180 $$\begin{aligned} \frac{\delta \mathcal{S}_\textrm{FRSB}}{\delta v(\textbf{k})}=&-\frac{s}{2}f'\left( 0\right) -\frac{s}{2w_M v(\textbf{k})}+\frac{1}{2(\lambda (\textbf{k},s)+s G_0(\textbf{k}))}-\frac{s}{2}\int _{s}^{w_M}\frac{dw}{w^2\,\lambda (\textbf{k},w)}\nonumber \\&=-\frac{s}{2}\left[ f'\left( 0\right) +\frac{G_0(\textbf{k})}{\lambda (\textbf{k},s)(\lambda (\textbf{k},s)+s G_0(\textbf{k}))}-\int _{s}^{w_M}\frac{dw\,\partial _w g(\textbf{k},w)}{\lambda (\textbf{k},w)^2}\right] =0\;. \end{aligned}$$

Note that the equation above matches the limit $z\rightarrow w_M$ of Eq. (176) and thus does not provide additional information. To analyse the optimal solution further, it will be convenient to introduce the following function

181 $$\begin{aligned} \sigma (\textbf{k},z)=\frac{1}{s(\lambda (\textbf{k},s)+s G_0(\textbf{k}))}-\frac{1}{z \lambda (\textbf{k},z)}-\int _{s}^{z}\frac{dw}{w^2\,\lambda (\textbf{k},w)}\;. \end{aligned}$$

This function can be shown to coincide with the Parisi function associated to the inverse overlap matrix $\textbf{G}(\textbf{k})^{-1}$. It can be used to re-express Eq. (176) as

182 $$\begin{aligned} \frac{\delta \mathcal{S}_\textrm{FRSB}}{\delta g(\textbf{k},z)} =\frac{s}{2}\left[ \sigma (\textbf{k},z)-f'\left( \int _\textbf{k}g(\textbf{k},z)\right) \right] =\frac{s}{2}\left[ \sigma (\textbf{k},z)-f'\left( Q(z)\right) \right] =0\;. \end{aligned}$$

Interestingly, from this expression, the optimal solution for that function is independent of the Fourier wave-vector $\textbf{k}$, i.e. $\sigma (\textbf{k},z)\equiv \sigma (z)$. The eigenvalue function of the inverse overlap matrix $\textbf{G}(\textbf{k})^{-1}$ is simply given by the inverse of that of $\textbf{G}(\textbf{k})$, yielding the simple identity

183 $$\begin{aligned} \frac{1}{\lambda (\textbf{k},z)}&=\frac{1}{v(\textbf{k})}+w_M \sigma (w_M)-z\,\sigma (z)-\int _{z}^{w_M}du\,\sigma (u)=\frac{1}{\lambda (\textbf{k},s)}+s\sigma (s)-z\sigma (z)\nonumber \\&\quad +\int _{s}^z du\,\sigma (u)\;. \end{aligned}$$

As Eq. (176) is valid over the whole interval $z\in [s,w_M]$, we may take an additional derivative with respect to *z* and obtain after some simplifications

184 $$\begin{aligned} \int _\textbf{k}\partial _z g(\textbf{k},z)f''\left( \int _\textbf{k}\,g(\textbf{k},z)\right) -\frac{\partial _z g(\textbf{k},z)}{\lambda (\textbf{k},z)^2}=Q'(z)f''\left( Q(z)\right) -\frac{\partial _z g(\textbf{k},z)}{\lambda (\textbf{k},z)^2}=0\;. \end{aligned}$$

Multiplying by $\lambda (\textbf{k},z)^2$ and integrating this equation with respect to the Fourier wavevector $\textbf{k}$ yields the simple identity

185 $$\begin{aligned} \int _\textbf{k}\partial _z g(\textbf{k},z)\left[ \int _\textbf{k}\lambda (\textbf{k},z)^2f''\left( \int _\textbf{k}\,g(\textbf{k},z)\right) -1\right] =Q'(z)\left[ \int _\textbf{k}\lambda (\textbf{k},z)^2 f''(Q(z))-1\right] =0\;. \end{aligned}$$

The FRSB solution must satisfy $\partial _z g(\textbf{k},z)> 0$ in the interval $z\in [s,w_M]$, thus one must have

186 $$\begin{aligned} f''\left( Q(z)\right) =f''\left( \int _\textbf{k}\,g(\textbf{k},z)\right) =\frac{1}{\int _\textbf{k}\lambda (\textbf{k},z)^2}\;. \end{aligned}$$

In particular, for $z=s$, using the identity $1/\lambda (\textbf{k},s)=\nu (\textbf{k})-s f'(Q(s))$, it yields the following self-consistent equation

187 $$\begin{aligned} f''\left( Q(s)\right) =\frac{1}{I_2(\mu -s f'(Q(s)))}\;. \end{aligned}$$

If the function $f''$ is bijective, it has a well-defined inverse ${f''}^{-1}$, allowing to express directly *Q*(*z*) as well as the optimal value of $\sigma (z)$, which satisfies the self-consistent equation

188 $$\begin{aligned}&\sigma (z)=f'\left[ {f''}^{-1}\left( \frac{1}{\int _\textbf{k}\lambda (\textbf{k},z)^2}\right) \right] \nonumber \\&\quad =f'\left[ {f''}^{-1}\left( \frac{1}{\int _\textbf{k}\left( \lambda (\textbf{k},s)^{-1}+s\sigma (s)-z\sigma (z)+\int _{s}^z du\,\sigma (u)\right) ^{-2}}\right) \right] \;. \end{aligned}$$

Note that from the expression above, one can express explicitly

189 $$\begin{aligned} \frac{1}{\lambda (\textbf{k},z)}&=\frac{1}{\lambda (\textbf{k},s)}+s f'(Q(s))-z f'(Q(z))+\int _s^z dw f'(Q(z))=\nu (\textbf{k})-z f'(Q(z)) \nonumber \\&\quad +\int _s^z dw f'(Q(w))\nonumber \\&=\nu (\textbf{k})-s f'(Q(s))-\int _{Q(s)}^{Q(z)}dq\,z(q)\,f''(q)\;. \end{aligned}$$

It yields the identity

190 $$\begin{aligned} f''(Q(z))&=\frac{1}{\int _\textbf{k}\left( \nu (\textbf{k})-s f'(Q(s))-\int _{Q(s)}^{Q(z)}dq\,z(q)\,f''(q)\right) ^{-2}}=\frac{1}{I_2(\mu (z,s))}\;,\end{aligned}$$

191 $$\begin{aligned} \mu (z,s)&=\mu -s f'(Q(s))-\int _{Q(s)}^{Q(z)}dq\,z(q)\,f''(q)=\mu -z\sigma (z)+\int _{s}^z du\,\sigma (u)\;. \end{aligned}$$

With that equation, one can simply express *Q*(*z*) or its inverse function *z*(*q*). Taking the form $\nu (\textbf{k})=\mu +\textbf{k}^2$, the integral above can be computed explicitly and the following expression for the function *z*(*q*) can be obtained

192 $$\begin{aligned} z(q)=-\frac{2}{4-d}B_d^{-\frac{2}{4-d}}\frac{f^{(3)}(q)}{f''(q)^{\frac{2(3-d)}{4-d}}}\;,\;\;B_d=-2^{d}\pi ^{\frac{d}{2}-1}\Gamma \left( \frac{d}{2}-1\right) \sin \left( \frac{d\pi }{2}\right) \;. \end{aligned}$$

Coming back to the general case and taking a derivative with respect to *z* finally yields

193 $$\begin{aligned}&z=-\frac{\left( \int _\textbf{k}\lambda (\textbf{k},z)^2\right) ^{3}}{2\int _\textbf{k}\lambda (\textbf{k},z)^3}f^{(3)}\left( {f''}^{-1}\left( \frac{1}{\int _\textbf{k}\lambda (\textbf{k},z)^2}\right) \right) \nonumber \\&\quad =-\frac{I_2(\mu (z,s))^3}{2I_3(\mu (z,s))}f^{(3)}\left( {f''}^{-1}\left( \frac{1}{I_2(\mu (z,s))}\right) \right) \;. \end{aligned}$$

This equation provides an explicit expression for *z* as a function of the combination $\mu (z,s)$, which can, after some manipulations, be used to obtain an explicit expression for $\sigma (z)$. In order for the solution to be truly FRSB, one must ensure that *Q*(*z*) is a non-increasing function of *z*, i.e. $\sigma (z)=f'(Q(z))$ is a non-increasing function of *z*. Taking a derivative with respect to *z* in Eq. (193), one obtains

194 $$\begin{aligned} -\frac{1}{z\sigma '(z)}=&\frac{3}{2}\left( 2-\frac{I_4(\mu (z,s))I_2(\mu (z,s))}{I_3(\mu (z,s))^2}\right) \frac{f^{(3)}\left( Q(z)\right) }{f''\left( Q(z)\right) ^2}-\frac{f^{(4)}\left( Q(z)\right) }{f^{(3)}\left( Q(z)\right) f''\left( Q(z)\right) }\;. \end{aligned}$$

Thus, if the right-hand-side above is positive, the solution will be full replica symmetry broken while it will have a finite level of replica symmetry breaking if the latter is negative.

The expression of $\lambda (\textbf{k},z)$ can be further simplified using the identity $\sigma (z)=f'(Q(z))$ as

195 $$\begin{aligned} \lambda (\textbf{k},z)&=\frac{1}{\lambda (\textbf{k},s)^{-1}+s\sigma (s)-z\sigma (z)+\int _{s}^z du\,\sigma (u)}=\frac{1}{\lambda (\textbf{k},s)^{-1}-\int _{Q(s)}^{Q(z)}dq\,z(q)\,f''(q)}\;. \end{aligned}$$

Using the identity $\partial _z \lambda (\textbf{k},z)=z \partial _z g(\textbf{k},z)$ as well as the boundary condition $g(\textbf{k},w_M)=0$, one obtains the expression

196 $$\begin{aligned} g(\textbf{k},z)=\int _{w_M}^z dw\,\frac{Q'(w)f''(Q(w))}{\displaystyle \left( \lambda (\textbf{k},s)^{-1}-\int _{Q(s)}^{Q(w)}dq\,z(q)\,f''(q)\right) ^2} =\int _{0}^{Q(z)}dq\,f''(q)\lambda (\textbf{k},q)^2\;, \end{aligned}$$

where $\lambda (\textbf{k},q)=\lambda (\textbf{k},z(q))$. With these expressions, we are now able to express the CGF explicitly as

197 $$\begin{aligned}&\varphi _{L,\mathrm FRSB}(s,\mu )=-\frac{s}{2}\int _\textbf{k}\nu (\textbf{k})\left[ g(\textbf{k},s)+G_0(\textbf{k})\right] \nonumber \\&+\frac{s}{2}\left( -f'(0)\int _\textbf{k}v(\textbf{k})+s\,f\left( Q(s)\right) +\int _{s}^{w_M}dz\,z\,Q'(z)\,f'\left( Q(z)\right) \right) \end{aligned}$$

198 $$\begin{aligned}&+\frac{1}{2}\int _\textbf{k}\left[ \ln \left( 1+\frac{s G_0(\textbf{k})}{\lambda (\textbf{k},s)}\right) -s\int _{s}^{w_M}\frac{dz}{z}\frac{\partial _z\lambda (\textbf{k},z)}{\lambda (\textbf{k},z)}\right] \end{aligned}$$

199 $$\begin{aligned} =&-\frac{s}{2}\int _0^{Q(s)}dq f''(q)\int _\textbf{k}\lambda (\textbf{k},q)^2\left( \nu (\textbf{k})-\frac{1}{\lambda (\textbf{k},q)}\right) +\frac{1}{2}\int _\textbf{k}\left[ \ln \left( 1+\frac{s G_0(\textbf{k})}{\lambda (\textbf{k},s)}\right) -s\frac{G_0(\textbf{k})}{\lambda (\textbf{k},s)}\right] \end{aligned}$$

200 $$\begin{aligned}&+\frac{s^2}{2}f'(Q(s))-\frac{s}{2}\int _{0}^{Q(s)}dq\, z(q) f'(q)+\frac{s}{2}\int _\textbf{k}\left[ -v(\textbf{k})+G_0(\textbf{k})\left( \frac{1}{\lambda (\textbf{k},s)}-\nu (\textbf{k})\right) \right] \end{aligned}$$

201 $$\begin{aligned} =&-\frac{s}{2}\int _0^{Q(s)}dq \left( s f'(Q(s))+\int _{Q(s)}^q dr\,z(r) f''(r)\right) f''(q)\int _\textbf{k}\lambda (\textbf{k},q)^2-\frac{s}{2}\int _{0}^{Q(s)}dq\, z(q) f'(q)\end{aligned}$$

202 $$\begin{aligned}&+\frac{1}{2}\int _\textbf{k}\left[ \ln \left( 1+\frac{s G_0(\textbf{k})}{\lambda (\textbf{k},s)}\right) -s\frac{G_0(\textbf{k})}{\lambda (\textbf{k},s)}\right] +\frac{s^2}{2}f'(Q(s))-\frac{s}{2}\int _\textbf{k}v(\textbf{k})-\frac{s^2}{2}f'(Q(s))\int _\textbf{k}G_0(\textbf{k})\end{aligned}$$

203 $$\begin{aligned} =&\frac{s^2}{2}\left( f(Q(s))-Q(s) f'(Q(s))\right) +\frac{1}{2}\int _\textbf{k}\left[ \ln \left( 1-\frac{s f'[Q(s)]}{\nu (\textbf{k})}\right) +\frac{s f'(Q(s))}{\nu (\textbf{k})-s f'(Q(s))}\right] \nonumber \\&+\frac{s}{2}\int _0^{Q(s)}z(q)\left[ q f''(q)-f'(q)\right] -\frac{s f'(0)}{2}\int _\textbf{k}\frac{1}{\nu (\textbf{k})-s f'(Q(s))+\int _0^{Q(s)}z(q)f''(q)dq}\;. \end{aligned}$$

Let us now consider the expression of the variance. The latter is computed from the properties of the typical solution obtained in the limit $s\rightarrow 0$. In particular, the important parameter is $Q_\textrm{typ}=Q(s=0)=\int _\textbf{k}g(\textbf{k},0)$. In that limit, the eigenvalue function and overlap read

204 $$\begin{aligned} \lambda (\textbf{k},z)&=\frac{1}{\lambda (\textbf{k},0)^{-1}-z\sigma (z)+\int _{0}^z du\,\sigma (u)}=\frac{1}{\nu (\textbf{k})-z\sigma (z)+\int _{0}^z du\,\sigma (u)}\;,\end{aligned}$$

205 $$\begin{aligned} Q(z)&={f''}^{-1}\left( \frac{1}{\int _\textbf{k}\left( \nu (\textbf{k})-z\sigma (z)+\int _{0}^z du\,\sigma (u)\right) ^{-2}}\right) \nonumber \\&={f''}^{-1}\left( \frac{1}{I_2\left( \mu -z\sigma (z)+\int _{0}^z du\,\sigma (u)\right) }\right) \;. \end{aligned}$$

Taking in particular its value as $z\rightarrow 0$, one obtains in the FRSB phase

206 $$\begin{aligned} Q_\textrm{typ}=Q(0)={f''}^{-1}\left( \frac{1}{I_2\left( \mu \right) }\right) \;. \end{aligned}$$

Using that expression, the variance can be computed explicitly as a function of $\mu$ as

207 $$\begin{aligned} \mathcal{V}_{L}(\mu )=&f(Q_\textrm{typ})-\frac{f'(Q_\textrm{typ})^2}{2f''(Q_\textrm{typ})} =f\left( {f''}^{-1}\left( \frac{1}{I_2(\mu )}\right) \right) -\frac{I_2(\mu )}{2}f'\left( {f''}^{-1}\left( \frac{1}{I_2(\mu )}\right) \right) ^2\;. \end{aligned}$$

The variance can be shown to display a second order phase transition as $\mu \rightarrow \mu _{c}$, with

208 $$\begin{aligned} \mathcal{V}_\textrm{FRSB}(\mu )-\mathcal{V}_\textrm{RS}(\mu )=2\frac{I_3(\mu _{c})^2 f''(0)^3}{f^{(3)}(0)}f'(0)(\mu -\mu _{c})^2+O(\mu -\mu _{c})^3\;. \end{aligned}$$

Comparing this result with Eq. (157), one obtains that the leading order behaviour at the transition is independent of whether the RSB transition is from a 1RSB or FRSB phase as long as the transition is continuous. From the identity

209 $$\begin{aligned} \int _\textbf{k} \frac{f''(Q(s))}{\left( \nu (\textbf{k})-s f'(Q(s))\right) ^2}=\int _\textbf{k} \frac{f''(0)}{\left( \nu (\textbf{k})-s f'(Q(s))+\int _0^{Q(s)}z(q)f''(q)dq\right) ^2}=1\;, \end{aligned}$$

one can derive the additional identity

210 $$\begin{aligned} Q'(s)=\frac{f'(Q(s))}{(z(Q(s))-s)f''(Q(s))}\;. \end{aligned}$$

It can then be used to show that one can associate an energy $e_{L,\mathrm FRSB}(s,\mu )$ to each value of *s*, of the form

211 $$\begin{aligned} e_{L,\mathrm FRSB}(s,\mu )&=-\partial _s\varphi _{L,\mathrm FRSB}(s,\mu )\nonumber \\&=-s f(0)-\frac{1}{2}\int _0^{Q}(z(q)-s)\left[ q f''(q)-f'(q)\right] \nonumber \\&\qquad +\frac{f'(0)}{2}\int _\textbf{k}\frac{1}{\nu (\textbf{k})-s f'(Q)+\int _0^{Q}z(q)f''(q)dq}\;. \end{aligned}$$

Taking an additional derivative with respect to *s*, one can check explicitly that the CGF is convex with

212 $$\begin{aligned} \partial _s^2\varphi _{L,\mathrm FRSB}(s,\mu )=f(Q(s))-\frac{f'(Q(s))^2}{2f''(Q(s))}> 0\;, \end{aligned}$$

for any $0\le Q(s)<\infty$. As the CGF is convex, one can compute explicitly its Legendre transform and obtain the large deviation function parametrically in terms of *s* as

213 $$\begin{aligned}&\Lambda _{L,\mathrm FRSB}(e=e_{L,\mathrm FRSB}(s,\mu ),\mu )=s\partial _s\varphi _{L,\mathrm FRSB}(s,\mu )-\varphi _{L,\mathrm FRSB}(s,\mu )\nonumber \\&\quad =\frac{s^2}{2}f\left( Q(s)\right) -\frac{1}{2}\int _\textbf{k}\left[ \ln \left( 1-\frac{s f'(Q(s))}{\nu (\textbf{k})}\right) +\frac{s f'(Q(s))}{\nu (\textbf{k})-s f'(Q(s))}\right] \;. \end{aligned}$$

For any $\mu >0$, the large deviation function behaves quadratically in the vicinity of the typical energy

214 $$\begin{aligned} \Lambda _{L,\mathrm FRSB}(e)&=\frac{\displaystyle (e-e_\textrm{typ})^2}{\displaystyle 2\mathcal{V}_L(\mu )}+O(e-e_\textrm{typ})^3\;,\end{aligned}$$

215 $$\begin{aligned} \textrm{with}\;\;e_\textrm{typ}&=e_{L,\mathrm FRSB}(0,\mu )=-\frac{1}{2}\int _0^{Q_\textrm{typ}}z(q)\left[ q f''(q)-f'(q)\right] \nonumber \\&\quad +\frac{f'(0)}{2}\int _\textbf{k}\frac{1}{\nu (\textbf{k})+\int _0^{Q_\textrm{typ}}z(q)f''(q)dq}\;. \end{aligned}$$

Let us consdier the transition between the RS and FRSB branch of the cumulant generating function and large deviation function. Using the definition of $s_\textrm{AT}$ in Eq. (137) as well as the equation for *Q*(*s*) in Eq. (187), one can check explicitly that $Q(s_\textrm{AT})=0$. Comparing Eq. (212) with Eq. (139), one concludes that the transition is at least of third order. Expanding the CGF difference, one obtains explicitly

216 $$\begin{aligned} \varphi _{L,\mathrm FRSB}(s,\mu )-\varphi _{L,\mathrm RS}(s,\mu )=\frac{I_3(\mu _c)^2 s_\textrm{AT}(f'(0)f''(0))^3}{3\left( f^{(3)}(0)+2s_\textrm{AT}f''(0)^3 I_3(\mu _c)\right) }(s-s_\textrm{AT})^3+O(s-s_\textrm{AT})^4\;, \end{aligned}$$

confirming that the transition is of third order, except for $\mu =\mu _{c}$ where $s_\textrm{AT}=0$ and the transition is of fourth order, i.e.

217 $$\begin{aligned} \varphi _{L,\mathrm FRSB}(s,\mu )-\varphi _{L,\mathrm RS}(s,\mu )=\frac{I_3(\mu )^2 (f'(0)f''(0))^3}{3f^{(3)}(0)}s^4+O(s)^5\;,\;\;\mu =\mu _{c}\;. \end{aligned}$$

Similarly the large deviation function difference behaves as

218 $$\begin{aligned}&\Lambda _{L,\mathrm FRSB}(e,\mu )-\Lambda _{L,\mathrm RS}(e,\mu )=\frac{I_3(\mu _c)^2 s_\textrm{AT}(f'(0)f''(0))^3}{3\mathcal{V}_L(\mu _c)^3\left( f^{(3)}(0)+2s_\textrm{AT}f''(0)^3 I_3(\mu _c)\right) }(e-e_\textrm{AT})^3 \nonumber \\&\quad +O(e-e_\textrm{AT})^4\;, \end{aligned}$$

which can be obtained simply from the behaviour of the CGF above and the identity

219 $$\begin{aligned} e_\textrm{FRSB}(s)\approx e_\textrm{RS}(s)\approx e_\textrm{AT}+\mathcal{V}_L(\mu _c)\left( s-s_\textrm{AT}\right) +O\left( s-s_\textrm{AT}\right) ^2\;. \end{aligned}$$

By comparing the result above with Eq. (170), one concludes that the leading behaviour of the large deviation function difference at the continuous transition is independent of the nature of the RSB phase (1RSB or FRSB).

Finally, let us consider the asymptotic limit as $s\rightarrow -\infty$. In that limit, one expects that $Q(s)>0$ and thus $\int _0^{Q(s)}z(q)f''(q)dq>0$. As $s\rightarrow -\infty$, one must ensure that $f'(Q(s))\rightarrow 0$ to ensure that the left-hand-side of Eq. (209) remains finite. Thus, in the limit, one must have that $Q(s)\rightarrow +\infty$ as $s\rightarrow -\infty$. On the other hand, from Eq. (187), as $f''(Q(s))\rightarrow 0$ in that limit, the integral $I_2(\mu -s f'(Q(s)))$ must diverge for their product to remain finite. Thus, one must have that at leading order $s f'(Q(s))\rightarrow \min _\textbf{k}\nu (\textbf{k})=\mu$. The energy can be rewritten as

220 $$\begin{aligned} e_{L,\mathrm FRSB}(s,\mu )=&-s f(Q(s))+\frac{s Q(s) f'(Q(s))}{2}-\frac{1}{2}\int _0^{Q(s)}z(q)\left[ q f''(q)-f'(q)\right] \nonumber \\&+\frac{f'(0)}{2}\int _\textbf{k}\frac{1}{\nu (\textbf{k})-s f'(Q)+\int _0^{Q}z(q)f''(q)dq}\;. \end{aligned}$$

In the limit $s\rightarrow -\infty$, one obtains at leading order $e_{L,\mathrm FRSB}(s,\mu )\sim \mu Q(s)/2$. Using that behaviour as well as the expression of the large-deviation function in Eq. (213), one obtains that the asymptotic behaviour as $s\rightarrow -\infty$, i.e. as $\epsilon \rightarrow +\infty$ reads

221 $$\begin{aligned} \Lambda _{L,\mathrm FRSB}(e,\mu )\approx \frac{\mu ^2 f\left( 2e/\mu \right) }{2f'\left( 2e/\mu \right) ^2}\;,\;\;e\rightarrow +\infty \;. \end{aligned}$$

Thus for any positive $\mu >0$, comparing with the result obtained in the 1RSB pattern in Eq. (175), the asymptotic behaviour of the large deviation function as $e\rightarrow +\infty$ does not seem to depend on the RSB pattern or the dimension but depends explicitly on the correlation function of the disorder. For models displaying a FRSB pattern as well and $\mu >0$, as the large deviation function is well-defined for any $e\in \mathbb {R}$, one does not expect a regime of large deviation with speed $N^2$, in contrast to what has been observed for spherical spin-glasses [21, 22]. In the following section, we apply our general results to a few special cases and consider in particular some cases where the unconfined limit $\mu \rightarrow 0$ can be taken.

This concludes our general analysis of the general properties of the different branch of the large deviation function. In the next section, we consider some special explicit cases.

## Additional Explicit Results: Exponential Correlation Function

In the following, we analyse the expression of the large deviation function in the continuous limit for an elastic term of the form $\nu (\textbf{k})=\mu +\textbf{k}^2$. The function that we consider in particular is

222 $$\begin{aligned} \mathcal{L}(\epsilon ,\mu )=\lim _{L\rightarrow \infty }\Lambda _L(e_{L,\mathrm typ}(\mu )+\epsilon ,\mu )\;. \end{aligned}$$

The analysis of the massless limit is done in some detail in section 2.4. In this section, we consider the exponential correlation function $f_{\infty }(q)=\exp (-c q)$ which corresponds to short-range disorder. The RSB pattern is of 1RSB type in dimensions $d<2$, it is marginal for $d=2$ and of FRSB type for $d>2$. In the following we detail the cases $d=1$ and $d=2$.

### Dimension One - 1RSB

In one dimension, the expression of the typical ground-state energy reads

223 $$\begin{aligned} e_\textrm{typ}(\mu )=\lim _{L\rightarrow \infty }e_{L,\mathrm typ}(\mu )=-\frac{c}{4\sqrt{\mu }}+w_\textrm{typ}(\mu )\left( e^{-c Q_\textrm{typ}(\mu )}-1+\frac{c Q_\textrm{typ}(\mu )}{2} \left[ 1+e^{-c Q_\textrm{typ}(\mu )}\right] \right) \;, \end{aligned}$$

where $Q_\textrm{typ}(\mu )=0$ and $w_\textrm{typ}(\mu )=0$ in the RS phase, i.e. for $\mu >\mu _c=(c/2)^{4/3}$, and is given in the 1RSB phase by the non-trivial solution of

224 $$\begin{aligned} Q_\textrm{typ}(\mu )&=\frac{1}{2w_\textrm{typ}(\mu )}\left( \frac{1}{\sqrt{\mu }}-\frac{1}{\sqrt{\mu +c w_\textrm{typ}(\mu )(1-e^{-c Q_\textrm{typ}(\mu )})}}\right) \;,\end{aligned}$$

225 $$\begin{aligned} e^{-c Q_\textrm{typ}(\mu )}(1+c Q_\textrm{typ}(\mu ))-1&=\frac{1}{w_\textrm{typ}(\mu )^2}\left( \sqrt{\mu }-\frac{2\mu +c w_\textrm{typ}(\mu )(1-e^{-c Q_\textrm{typ}(\mu )})}{2\sqrt{\mu +c w_\textrm{typ}(\mu )(1-e^{-c Q_\textrm{typ}(\mu )})}}\right) \;. \end{aligned}$$

The large deviation function displays both a RS and a 1RSB branch. The transition between the two branches is discontinuous (in terms of the order parameter *Q*) for $\mu <\mu _\textrm{dis}=(c/2)^{4/3}/3=\mu _c/3$ and occurs at the energy

226 $$\begin{aligned} e_\textrm{dis}(\mu )=-s_\textrm{dis}(\mu )-\frac{c}{4\sqrt{\mu +c s_\textrm{dis}(\mu )}}\;, \end{aligned}$$

where $s_\textrm{dis}(\mu )$ is the solution of the equation

227 $$\begin{aligned}&Q_\textrm{dis}(\mu )=\frac{1}{2s_\textrm{dis}(\mu )}\left( \frac{1}{\sqrt{\mu +c s_\textrm{dis}(\mu ) e^{-c Q_\textrm{dis}(\mu )}}}-\frac{1}{\sqrt{\mu +c s_\textrm{dis}(\mu )}}\right) \;,\end{aligned}$$

228 $$\begin{aligned}&\quad 1-e^{-Q_\textrm{dis}(\mu )}(1+c Q_\textrm{dis}(\mu )) \nonumber \\&\quad =\frac{1}{s_\textrm{dis}(\mu )^2}\left[ \sqrt{\mu +c s_\textrm{dis}(\mu )}-\sqrt{\mu +c s_\textrm{dis}(\mu )e^{-c Q_\textrm{dis}(\mu )}}-\frac{c s_\textrm{dis}(\mu )(1-e^{-c Q_\textrm{dis}(\mu )})}{2\sqrt{\mu +c s_\textrm{dis}(\mu )}}\right] \;. \end{aligned}$$

For $\mu >\mu _\textrm{dis}=(c/2)^{4/3}/3$, the transition is continuous (in terms of the order parameter *Q*) and occurs at the energy

229 $$\begin{aligned} e_\textrm{AT}(\mu )=\mu -\left( \frac{c}{2}\right) ^{1/3}\;. \end{aligned}$$

Using these expressions, one can plot the phase diagram in Fig. 2. The transition line satisfies

230 $$\begin{aligned} \epsilon _\textrm{t}(\mu )={\left\{ \begin{array}{ll} \displaystyle \epsilon _\textrm{dis}(\mu )=e_\textrm{dis}(\mu )-e_\textrm{typ}(\mu )=-s_\textrm{dis}(\mu )-\frac{c}{4\sqrt{\mu +c s_\textrm{dis}(\mu )}}-e_\textrm{typ}(\mu )& \;,\;\;\mu \le \mu _\textrm{dis}=\frac{1}{3}\left( \frac{c}{2}\right) ^{4/3}\;, \\ \displaystyle \epsilon _\textrm{AT}(\mu )=e_\textrm{AT}(\mu )-e_\textrm{typ}(\mu )=\frac{\mu }{2}-\left( \frac{c}{2}\right) ^{1/3}-e_\textrm{typ}(\mu )& \;,\;\;\mu > \mu _\textrm{dis}\;. \end{array}\right. } \end{aligned}$$

The large deviation function can be expressed as

231 $$\begin{aligned} \mathcal{L}(\epsilon ,\mu )&={\left\{ \begin{array}{ll} \displaystyle \frac{1}{2}\left( s_\textrm{RS}(\epsilon )^2+\sqrt{\mu }-\sqrt{\mu +c s_\textrm{RS}(\epsilon )}+\frac{c s_\textrm{RS}(\epsilon )}{2\sqrt{\mu +s_\textrm{RS}(\epsilon )}}\right) & \;,\;\;\epsilon \le \epsilon _\textrm{t}(\mu )\;,\\ & \\ \displaystyle \frac{1}{2}\left( s_{1}(\epsilon )^2 e^{-c Q_1(\epsilon )}+\sqrt{\mu }-\sqrt{\mu +c s_{1}(\epsilon )e^{-c Q_1(\epsilon )}}+\frac{c s_{1}(\epsilon )e^{-c Q_1(\epsilon )}}{2\sqrt{\mu +c s_{1}(\epsilon )e^{-c Q_1(\epsilon )}}}\right) & \;,\;\;\epsilon >\epsilon _\textrm{t}(\mu )\;, \end{array}\right. }\end{aligned}$$

232 $$\begin{aligned} \epsilon&=-s_\textrm{RS}(\epsilon )-\frac{c}{4\sqrt{\mu +c s_\textrm{RS}(\epsilon )}}-e_\textrm{typ}(\mu )\;,\end{aligned}$$

233 $$\begin{aligned} \epsilon&=-w(\epsilon )-(s_{1}(\epsilon )-w(\epsilon ))e^{-c Q_1(\epsilon )}\left( 1-\frac{c Q_1(\epsilon )}{2}\right) \nonumber \\&-\frac{1}{4\sqrt{\mu +c s_{1}(\epsilon )e^{-c Q_1(\epsilon )}+c w(\epsilon )(1-e^{-c Q_1(\epsilon )})}}-e_\textrm{typ}(\mu )\;, \end{aligned}$$

where the parameters $w(\epsilon )$ and $Q_1(\epsilon )$ are solution of

234 $$\begin{aligned}&Q_1(\epsilon )\nonumber \\&=\frac{1}{2w(\epsilon )}\left( \frac{1}{\sqrt{\mu +c s_{1}(\epsilon )e^{-c Q_1(\epsilon )}}}-\frac{1}{\sqrt{\mu +c s_{1}(\epsilon )e^{-c Q_1(\epsilon )}+c w(\epsilon )(1-e^{-c Q_1(\epsilon )})}}\right) \;, \end{aligned}$$

235 $$\begin{aligned}&1-e^{-c Q_1(\epsilon )}(1+c Q_1(\epsilon ))\nonumber \\&=\frac{1}{w(\epsilon )^2}\left[ \sqrt{\mu +c s_{1}(\epsilon )e^{-c Q_1(\epsilon )}+c w(\epsilon )(1-e^{-Q_1(\epsilon )})}-\sqrt{\mu +c s_{1}(\epsilon )e^{-c Q_1(\epsilon )}}\right] \nonumber \\&-\frac{c(1-e^{-c Q_1(\epsilon )})}{2w(\epsilon )\sqrt{\mu +c s_{1}(\epsilon )e^{-c Q_1(\epsilon )}+c w(\epsilon )(1-e^{-c Q_1(\epsilon )})}}\;. \end{aligned}$$

In the massless limit, the ground-state energy is finite and reads

236 $$\begin{aligned} e_\textrm{typ}(0)=-3\left( \frac{c}{4}\right) ^{1/3}\;. \end{aligned}$$

The large deviation function only displays a RS branch and is given parametrically as

237 $$\begin{aligned}&\mathcal{L}(\epsilon )=\mathcal{L}(\epsilon ,0)=\frac{\sqrt{s_\textrm{RS}(\epsilon )}}{2}\left( s_\textrm{RS}(\epsilon )^{3/2}-\frac{\sqrt{c}}{2}\right) \;, \nonumber \\&\epsilon =-s_\textrm{RS}(\epsilon )-\frac{1}{4}\sqrt{\frac{c}{s_\textrm{RS}(\epsilon )}}+3\left( \frac{c}{4}\right) ^{1/3}\;,\;\;\epsilon \le 0\;. \end{aligned}$$

The LDF vanishes linearly in that limit with $\mathcal{L}'(0)=-(c/2)^{1/3}$.

### Dimension Two - Marginal RSB

The expression for the large deviation function is much more explicit in dimension $d=2$. In that case, the transition occurs at a value of the energy difference satisfying

238 $$\begin{aligned} \epsilon _t(\mu )&=\epsilon _\textrm{AT}(\mu )=\frac{\mu }{c}-\frac{c}{4\pi }-\frac{c}{8\pi }\ln \frac{4\pi \mu }{c^2}-\epsilon _\textrm{RSB}(\mu )\Theta (\mu _c-\mu )\;,\end{aligned}$$

239 $$\begin{aligned} \epsilon _\textrm{RSB}(\mu )&=\frac{\mu }{c}-\frac{c}{4\pi }-\frac{c^2+4\pi \mu }{8\pi c}\ln \frac{4\pi \mu }{c^2}\;, \end{aligned}$$

where $\mu _c=\frac{c^2}{4\pi }$. The large deviation function reads

240 $$\begin{aligned} \mathcal{L}(\epsilon ,\mu )={\left\{ \begin{array}{ll} \displaystyle \mathcal{L}_\textrm{RS}(\epsilon +\epsilon _\textrm{RSB}(\mu )\Theta (\mu _c-\mu ),\mu )\;,\;\;\epsilon \le \epsilon _\textrm{AT}(\mu )\;, \\ \displaystyle \mathcal{L}_\textrm{RSB}(\epsilon -\epsilon _\textrm{RSB}(\mu )\Theta (\mu -\mu _c),\mu )\;,\;\;\epsilon > \epsilon _\textrm{AT}(\mu )\;. \end{array}\right. } \end{aligned}$$

where the RS branch reads

241 $$\begin{aligned} \mathcal{L}_\textrm{RS}(\epsilon ,\mu )=&\frac{\mu }{8\pi } \left( 1+\frac{4\pi \mu }{c^2}+W_{-1}\left( -\frac{8\pi \mu }{c^2}e^{\frac{8\pi (c\epsilon -\mu )}{c^2}}\right) +\ln \left( -\frac{c^2}{8\pi \mu }W_{-1}\left( -\frac{8\pi \mu }{c^2}e^{\frac{8\pi (c\epsilon -\mu )}{c^2}}\right) \right) \right) \nonumber \\&+\frac{c^2}{128\pi ^2}W_{-1}\left( -\frac{8\pi \mu }{c^2}e^{\frac{8\pi (c\epsilon -\mu )}{c^2}}\right) \left( 2+W_{-1}\left( -\frac{8\pi \mu }{c^2}e^{\frac{8\pi (c\epsilon -\mu )}{c^2}}\right) \right) \;, \end{aligned}$$

with $W_{-1}(x)$ is the $-1$ branch of the Lambert W function, while the RSB branch reads

242 $$\begin{aligned} \mathcal{L}_\textrm{RSB}(\epsilon ,\mu )=\frac{\mu }{8\pi }\left( e^{\frac{2c\epsilon }{\mu }}-\frac{2c\epsilon }{\mu }-1\right) \;. \end{aligned}$$

Using the expressions above, one can check that the transition between the two branches is generally of third order apart for $\mu =\mu _{c}$ where the transition is of fourth order. Similarly, both branches vanish quadratically as $\epsilon \rightarrow 0$ for any finite $\mu$, which is consistent with a central limit theorem for typical fluctuation. In the limit where $\mu \rightarrow 0$, the large deviation function only displays an RS branch and reads

243 $$\begin{aligned}&\mathcal{L}(\epsilon )=\lim _{\mu \rightarrow 0}\mathcal{L}_\textrm{RS}(\epsilon +\epsilon _\textrm{RSB}(\mu ),\mu )\end{aligned}$$

244 $$\begin{aligned}&\quad =\frac{c^2}{128\pi ^2}W_{-1}\left( -2e^{\frac{8\pi }{c}\epsilon -2}\right) \left( 2+W_{-1}\left( -2e^{\frac{8\pi }{c}\epsilon -2}\right) \right) \;,\;\;\epsilon \le 0\;. \end{aligned}$$

Note that in this limit instead, the large deviation function vanishes linearly with $\mathcal{L}'(0)=-c/(4\pi )$.

### Dimension Three - FRSB

In dimension three, the large deviation function displays a RS and a FRSB branch. The transition occurs at an energy difference

245 $$\begin{aligned} \epsilon _t(\mu )=\epsilon _\textrm{AT}(\mu )&=\frac{\mu }{c}-\frac{c\sqrt{\mu }}{8\pi }-\epsilon _\textrm{FRSB}(\mu )\Theta \left( \mu _c-\mu \right) \;,\end{aligned}$$

246 $$\begin{aligned} \epsilon _\textrm{FRSB}(\mu )&=\frac{1}{4}\left( \frac{c^3}{(8\pi )^2}- \frac{c\sqrt{\mu }}{2\pi }+\frac{3\mu }{c}-\frac{\mu }{c}\ln \frac{(8\pi )^2\mu }{c^4}\right) \;, \end{aligned}$$

with $\mu _c=\left( c^2/(8\pi )\right) ^2$ and where we used that in $d=3$ one has $z(q)=-f^{(3)}(q)/(32\pi ^2)=(c^3/(32\pi ^2)) e^{-c q}$ and $Q_\textrm{typ}(\mu )=1/(2c)\ln (\mu _c/\mu )\Theta (\mu _c-\mu )$. Using the expression above, one can obtain the phase diagram in Fig. 3. The large deviation function reads

247 $$\begin{aligned} \mathcal{L}(\epsilon ,\mu )={\left\{ \begin{array}{ll} \displaystyle \mathcal{L}_\textrm{RS}(\epsilon +\epsilon _\textrm{FRSB}(\mu )\Theta (\mu _c-\mu ),\mu )& \;,\;\;\epsilon \le \epsilon _\textrm{AT}(\mu )\;, \\ \displaystyle \mathcal{L}_\textrm{FRSB}(\epsilon +\epsilon _\textrm{FRSB}(\mu )\Theta (\mu _c-\mu ),\mu )& \;,\;\;\epsilon > \epsilon _\textrm{AT}(\mu )\;, \end{array}\right. } \end{aligned}$$

where the RS branch is expressed as

248 $$\begin{aligned} \mathcal{L}_\textrm{RS}(\epsilon )=&\frac{s_\textrm{RS}(\epsilon )^2}{2}+\frac{2\mu \sqrt{\mu +c s_\textrm{RS}(\epsilon )}-2\mu ^{3/2}-c s_\textrm{RS}(\epsilon )\sqrt{\mu +c s_\textrm{RS}(\epsilon ) }}{24\pi }\;,\end{aligned}$$

249 $$\begin{aligned} \textrm{with}\;\;s_\textrm{RS}(\epsilon )=&-\epsilon +\frac{c}{2(8\pi )^2}\left( c^2-16\pi \sqrt{\mu }+\sqrt{(c^2-16\pi \sqrt{\mu })^2-4(8\pi )^2 c\epsilon }\right) \;, \end{aligned}$$

while the FRSB branch is expressed as

250 $$\begin{aligned} \mathcal{L}_\textrm{FRSB}(\epsilon )&=\frac{c^6}{6(8\pi )^4}e^{-3c Q(\epsilon )}+\frac{\mu ^2}{2c^2}e^{c Q(\epsilon )}-\frac{\mu ^{3/2}}{12\pi }\;,\end{aligned}$$

251 $$\begin{aligned} \textrm{with}\;\;Q(\epsilon )&=\frac{2\epsilon }{\mu }-\frac{(c^2-16\pi \sqrt{\mu })^2}{2(8\pi )^2\mu c}+\frac{1}{2c} W_{-1}\left( \frac{c^4}{(8\pi )^2\mu }e^{-2c\left( \frac{2\epsilon }{\mu }-\frac{(c^2-16\pi \sqrt{\mu })^2}{2(8\pi )^2\mu c}\right) }\right) \;. \end{aligned}$$

In the massless limit, the transition occurs at the value

252 $$\begin{aligned} \epsilon _t(0)=\epsilon _\textrm{AT}(0)=-\epsilon _\textrm{FRSB}(0)=-\frac{c^3}{4(8\pi )^2}\;. \end{aligned}$$

The LDF still has two branches given by

253 $$\begin{aligned}&\mathcal{L}(\epsilon )=\mathcal{L}(\epsilon ,0)\end{aligned}$$

254 $$\begin{aligned}&\quad ={\left\{ \begin{array}{ll} \displaystyle \frac{1}{2}\left( -\epsilon +\frac{c^3}{4(8\pi )^2}+\frac{c}{8\pi }\sqrt{-c \epsilon }\right) ^2-\frac{c^{3/2}}{24\pi }\left( -\epsilon +\frac{c^3}{4(8\pi )^2}+\frac{c}{8\pi }\sqrt{-c \epsilon }\right) ^{3/2}& \;,\;\;\epsilon \le \epsilon _\textrm{AT}(0)\;, \\ \displaystyle \frac{(-c \epsilon )^{3/2}}{6\pi }& \;,\;\;\epsilon > \epsilon _\textrm{AT}(0)\;. \end{array}\right. } \end{aligned}$$

In particular, the LDF vanishes with a nontrivial exponent 3/2. This concludes our analysis of the disorder with (short-range) exponential correlation function.

## Conclusion and Discussion

We have computed explicitly the cumulant generating function and large deviation function of the rescaled ground-state energy $e_{\min }=\frac{1}{NL^d}\min _\textbf{u}\mathcal{H}[\textbf{u}]$ of the elastic manifold with internal dimension $0<d<4$ and in the limit $N\gg 1$ using replica computations. We have shown that for any positive mass $\mu >0$, the typical fluctuations of $e_{\min }$ are normal and of order $\sim (NL^d)^{-1/2}$. The associated cumulant generating function is expressed as a Parisi formula. The large deviation function $\Lambda _L(e,\mu )$ of speed $NL^d$, describing atypical fluctuations of order *O*(1) of the energy is well defined for any $e\in \mathbb {R}$ and displays both a replica symmetric and replica symmetry broken branch for any $\mu >0$. In the massless and continuous limit $\mu =0$, the ground-state energy super-concentrates and typical fluctuations are of order $\sim N^{-1/\xi }$ where $\xi <2$ is computed explicitly. In that limit, the large deviation function $\mathcal{L}(\epsilon )=\lim _{L\rightarrow \infty }\Lambda _L(\epsilon +e_{L,\mathrm typ}(0),0)$ of speed $NL^d$ is one-sided, describing only atypical fluctuations with $\epsilon <0$ and vanishes with an exponent $\mathcal{L}(\epsilon )\sim (-\epsilon )^{\xi }$. We thus conjecture that the distribution of typical fluctuations displays a left tail with the same exponent $\xi$.

## Typical Ground-State Energy

The average and typical ground-state energy $e_\textrm{typ}\equiv e_{L,\mathrm typ}(\mu )$ only takes a finite value for an internal dimension $d<2$. It is obtained as

A1 $$\begin{aligned} e_\textrm{typ}=\lim _{NL^d\rightarrow \infty }\left\langle e_{\min } \right\rangle =-\partial _s\varphi _L(0,\mu )=\displaystyle \max _{g(\textbf{k},z),G_0(\textbf{k}),v(\textbf{k}),w_M}\mathcal{S}_{L,\mathrm typ}(\mu ) \end{aligned}$$

where the functional $\mathcal{S}_\textrm{typ}$ is defined as

A2 $$\begin{aligned}&\mathcal{S}_{L,\mathrm typ}(\mu )=\frac{1}{2}\int _\textbf{k}\nu (\textbf{k})g(\textbf{k},0)+\frac{1}{2}\left[ f'(0)\int _\textbf{k}v(\textbf{k})-w_M\,f\left( 0\right) +\int _{0}^{w_M}dz\,f\left( \int _\textbf{k}g(\textbf{k},z)\right) \right] \nonumber \\&\quad +\frac{1}{2}\int _\textbf{k}\left[ \frac{1}{w_M}\ln \frac{v(\textbf{k})}{\lambda (\textbf{k},0)}+\int _{0}^{w_M}\frac{dz}{z^2}\ln \frac{\lambda (\textbf{k},z)}{\lambda (\textbf{k},0)}\right] +\frac{1}{2}\int _\textbf{k}G_0(\textbf{k})\left[ \nu (\textbf{k})-\frac{1}{\lambda (\textbf{k},0)}\right] \;. \end{aligned}$$

where the function $\lambda (\textbf{k},z)$ is defined as

A3 $$\begin{aligned} \lambda (\textbf{k},z)=v(\textbf{k})+z\,g(\textbf{k},z)+\int _{z}^{w_M}dw\,g(\textbf{k},w)\;, \end{aligned}$$

and the function $g(\textbf{k},z)$ satisfies the boundary condition $g(\textbf{k},z\ge w_M)=0$.

Let us denote with an index _typ_ the optimal value of the different parameters to optimise. The corresponding minimazation equations read

A4 $$\begin{aligned}&\frac{\partial \mathcal{S}_\textrm{typ}}{\partial w_M}[g_\textrm{typ},v_\textrm{typ},G_\textrm{typ},w_\textrm{typ}]=0\end{aligned}$$

A5 $$\begin{aligned}&=\frac{f\left( \int _\textbf{k}g_\textrm{typ}(\textbf{k},w_\textrm{typ})\right) -f(0)}{2}\nonumber \\&+\int _\textbf{k}\frac{g_\textrm{typ}(\textbf{k},w_\textrm{typ})}{2}\left[ \int _0^{w_\textrm{typ}}\frac{1}{z^2}\left( \frac{1}{\lambda _\textrm{typ}(\textbf{k},0)}-\frac{1}{\lambda _\textrm{typ}(\textbf{k},z)}\right) +\frac{G_\textrm{typ}(\textbf{k})}{\lambda _\textrm{typ}(\textbf{k},0)^2}-\frac{1}{w_\textrm{typ}\lambda _\textrm{typ}(\textbf{k},0)}\right] \;,\nonumber \\&\frac{\delta \mathcal{S}_\textrm{typ}}{\delta G_0(\textbf{k})}[g_\textrm{typ},v_\textrm{typ},G_\textrm{typ},w_\textrm{typ}]=0=\frac{1}{2}\left[ \nu (\textbf{k})-\frac{1}{\lambda _\textrm{typ}(\textbf{k},0)}\right] \;,\end{aligned}$$

A6 $$\begin{aligned}&\frac{\delta \mathcal{S}_\textrm{typ}}{\delta v(\textbf{k})}[g_\textrm{typ},v_\textrm{typ},G_\textrm{typ},w_\textrm{typ}]=0\end{aligned}$$

A7 $$\begin{aligned}&=\frac{1}{2}\left[ f'(0)+\frac{G_\textrm{typ}(\textbf{k})}{\lambda _\textrm{typ}(\textbf{k},0)^2}+\frac{1}{w_\textrm{typ}}\left( \frac{1}{v_\textrm{typ}(\textbf{k})}-\frac{1}{\lambda _\textrm{typ}(\textbf{k},0)}\right) +\int _0^{w_\textrm{typ}}\frac{dz}{z^2}\left( \frac{1}{\lambda _\textrm{typ}(\textbf{k},z)}-\frac{1}{\lambda _\textrm{typ}(\textbf{k},0)}\right) \right] \;,\nonumber \\&\frac{\delta \mathcal{S}_\textrm{typ}}{\delta g(\textbf{k},z_0)}[g_\textrm{typ},v_\textrm{typ},G_\textrm{typ},w_\textrm{typ}]=0\end{aligned}$$

A8 $$\begin{aligned}&=\frac{1}{2}\left[ f'\left( \int _\textbf{k}g_\textrm{typ}(\textbf{k},z_0)\right) +\frac{G_\textrm{typ}(\textbf{k})}{\lambda _\textrm{typ}(\textbf{k},0)^2}+\frac{1}{z_0}\left( \frac{1}{\lambda _\textrm{typ}(\textbf{k},z_0)}-\frac{1}{\lambda _\textrm{typ}(\textbf{k},0)}\right) \right. \nonumber \\&\quad \left. +\int _0^{z_0}\frac{dz}{z^2}\left( \frac{1}{\lambda _\textrm{typ}(\textbf{k},z)}-\frac{1}{\lambda _\textrm{typ}(\textbf{k},0)}\right) \right] \;. \end{aligned}$$

Note that the first equation is trivially ensured by imposing the boundary condition $g_\textrm{typ}(\textbf{k},z\ge w_\textrm{typ})=0$. Taking the limit $z_0\rightarrow 0$ in Eq. (A7) and using Eq. (A5) yields the simple relation

A9 $$\begin{aligned} G_\textrm{typ}(\textbf{k})=-\frac{\displaystyle f'\left( \int _\textbf{k}g_\textrm{typ}(\textbf{k},0)\right) }{\displaystyle \nu (\textbf{k})^2}=-\frac{f'\left( Q_\textrm{typ}\right) }{\nu (\textbf{k})^2}\;,\;\;\textrm{with}\;\;Q_\textrm{typ}=\int _\textbf{k}g_\textrm{typ}(\textbf{k},0)\;. \end{aligned}$$

In the replica-symmetric phase, corresponding either to $w_\textrm{typ}=0$ or equivalently to $g(\textbf{k},z)\equiv 0$, the parameters take simple expressions

A10 $$\begin{aligned} \lambda _\textrm{typ}(\textbf{k},0)=v_\textrm{typ}(\textbf{k})=\frac{1}{\nu (\textbf{k})}\;,\;\;G_\textrm{typ}(\textbf{k})=-\frac{f'\left( 0\right) }{\nu (\textbf{k})^2}\;,\;\;Q_\textrm{typ}=0\;. \end{aligned}$$

The expression for the average ground-state energy simplifies drastically, yielding

A11 $$\begin{aligned} e_{L,\mathrm RS}(\mu )=-\frac{f'(0)}{2}\int _\textbf{k}\frac{1}{\nu (\textbf{k})}\;. \end{aligned}$$

In the 1RSB phase instead the function $g_\textrm{typ}(\textbf{k},z)=g_\textrm{typ}(\textbf{k})\Theta (w_\textrm{typ}-z)$, where $g_\textrm{typ}(\textbf{k})$ and $w_\textrm{typ}$ optimal values of the corresponding parameters. In addition to the equations above, which remain valid, one needs to consider the optimisation over $w_\textrm{typ}$, yielding

A12 $$\begin{aligned} \frac{1}{2}\left( f\left( \int _\textbf{k}g_\textrm{typ}(\textbf{k})\right) -f(0)\right) +\frac{1}{2}\int _\textbf{k}\left[ \frac{g_\textrm{typ}(\textbf{k})}{\lambda _\textrm{typ}(\textbf{k},0)}\left( \frac{G_\textrm{typ}(\textbf{k})}{\lambda _\textrm{typ}(\textbf{k},0)}-\frac{1}{w_\textrm{typ}}\right) -\frac{1}{w_\textrm{typ}^2}\ln \frac{v_\textrm{typ}(\textbf{k})}{\lambda _\textrm{typ}(\textbf{k},0)}\right] =0\;, \end{aligned}$$

where $\lambda _\textrm{typ}(\textbf{k},0)=v_\textrm{typ}(\textbf{k})+w_\textrm{typ}g_\textrm{typ}(\textbf{k})$. The equations read explicitly

A13 $$\begin{aligned}&\lambda _\textrm{typ}(\textbf{k},0)=v_\textrm{typ}(\textbf{k})+w_\textrm{typ}g_\textrm{typ}(\textbf{k})=\frac{1}{\nu (\textbf{k})}\;,\end{aligned}$$

A14 $$\begin{aligned}&G_\textrm{typ}(\textbf{k})=-\frac{f'\left( Q_\textrm{typ}\right) }{\nu (\textbf{k})^2}\;,\;\;Q_\textrm{typ}=\int _\textbf{k}g_\textrm{typ}(\textbf{k})\;,\end{aligned}$$

A15 $$\begin{aligned}&f'(0)-f'\left( Q_\textrm{typ}\right) +\frac{1}{w_\textrm{typ}}\left( \left( \frac{1}{\nu (\textbf{k})}-w_\textrm{typ}g_\textrm{typ}(\textbf{k})\right) ^{-1}-\nu (\textbf{k})\right) =f'(0)-f'\left( Q_\textrm{typ}\right) \nonumber \\&+\frac{\nu (\textbf{k})^2 g_\textrm{typ}(\textbf{k})}{1-\nu (\textbf{k}) w_\textrm{typ}g_\textrm{typ}(\textbf{k})}=0\;, \end{aligned}$$

where $g_\textrm{typ}(\textbf{k})$ can be obtained explicitly from the last equation and $Q_\textrm{typ}$ can be shown to satisfy a self-consistent equation reading

A16 $$\begin{aligned} g_\textrm{typ}(\textbf{k})&=\frac{1}{w_\textrm{typ}}\left( \frac{1}{\nu (\textbf{k})}-\frac{1}{\nu (\textbf{k})+w_\textrm{typ}(f'\left( Q_\textrm{typ}\right) -f'(0))}\right) \;,\end{aligned}$$

A17 $$\begin{aligned} Q_\textrm{typ}&=\int _\textbf{k}\frac{1}{w_\textrm{typ}}\left( \frac{1}{\nu (\textbf{k})}-\frac{1}{\nu (\textbf{k})+w_\textrm{typ}(f'\left( Q_\textrm{typ}\right) -f'(0))}\right) \;. \end{aligned}$$

The remaining equation can be rewritten as

A18 $$\begin{aligned}  &   f\left( Q_\textrm{typ}\right) -f(0)-Q_\textrm{typ} f'\left( Q_\textrm{typ}\right) \nonumber \\= &   \frac{1}{w_\textrm{typ}^2}\int _\textbf{k}\left[ \frac{w_\textrm{typ}(f'\left( Q_\textrm{typ}\right) -f'(0))}{\nu (\textbf{k})+w_\textrm{typ}(f'\left( Q_\textrm{typ}\right) -f'(0))}-\ln \left( 1+\frac{w_\textrm{typ}(f'\left( Q_\textrm{typ}\right) -f'(0))}{\nu (\textbf{k})}\right) \right] \;. \end{aligned}$$

The expression of the ground-state energy can be simplified using these identities and reads

A19 $$\begin{aligned} e_{L,\mathrm 1RSB}(\mu )=e_{L,\mathrm RS}(\mu )+w_\textrm{typ}\left( f(Q_\textrm{typ})-f(0)-\frac{Q_\textrm{typ}}{2} \left[ f'(Q_\textrm{typ})+f'(0)\right] \right) \;. \end{aligned}$$

In particular, expanding in powers of $Q_\textrm{typ}$, one observed that the continuous transition from the RS to the 1RSB phase, occurring as $Q_\textrm{typ}\rightarrow 0$, is of third order. A priori, a discontinuous transition could occur if the parameter $w_\textrm{typ}\rightarrow 0$ for a finite value of $Q_\textrm{typ}$. Analysing Eqs. (A17-A18) in the limit $w_\textrm{typ}\rightarrow 0$, one realises that the value $Q_\textrm{d}$ at this transition must satisfy

A20 $$\begin{aligned} f(0)-f(Q_\textrm{d})+\frac{Q_\textrm{d}}{2} \left[ f'(Q_\textrm{d})+f'(0)\right] =0 \end{aligned}$$

for a value $Q_\textrm{d}>0$. If the function $f'(q)$ is strictly concave, this equation only has the solution $Q_\textrm{d}=0$. As for short-range potentials $f^{(3)}(q)=-\int dk\,k^6\,\tilde{f}(k)\,e^{-q k^2}<0$ with $\tilde{f}(k)\ge 0$ for any finite *q*, this is always the case.

Finally, in the full replica symmetry broken phase, One may rewrite Eq. (A7) as

A21 $$\begin{aligned} f'\left( \int _\textbf{k}g_\textrm{typ}(\textbf{k},z)\right) =f'(Q_\textrm{typ}(z))=\sigma _\textrm{typ}(z)=\int _0^{z}dw\frac{\partial _w g_\textrm{typ}(\textbf{k},w)}{\lambda _\textrm{typ}(\textbf{k},w)^2}-\frac{G_\textrm{typ}(\textbf{k})}{\lambda _\textrm{typ}(\textbf{k},0)^2}\;, \end{aligned}$$

which must be valid in the whole range $z_0\in [0,w_\textrm{typ}]$. One may check explicitly that the function $\sigma _\textrm{typ}(z)$ is the Parisi function associated to the coefficients $G_l(\textbf{k})^{-1}$. Thus, there exists a non-trivial relationship between the Parisi function $g_\textrm{typ}(\textbf{k},z)$ with coefficients $G_l(\textbf{k})$ and the Parisi function $\sigma _\textrm{typ}(z)$ which is most simply expressed as

A22 $$\begin{aligned}&-z\sigma _\textrm{typ}(z)+\int _{0}^z du\,\sigma _\textrm{typ}(u) \nonumber \\&=\frac{1}{\lambda _\textrm{typ}(\textbf{k},z)}-\frac{1}{\lambda _\textrm{typ}(\textbf{k},0)}\nonumber \\&=\frac{1}{v_\textrm{typ}(\textbf{k})+z g_\textrm{typ}(\textbf{k},z) +\int _{z}^{w_\textrm{typ}} du\,g_\textrm{typ}(\textbf{k},u)}-\frac{1}{v_\textrm{typ}(\textbf{k})+\int _{0}^{w_\textrm{typ}} du\,g_\textrm{typ}(\textbf{k},u)}\nonumber \\&=\left( \frac{1}{\nu (\textbf{k})}+z g_\textrm{typ}(\textbf{k},z) +\int _{z}^{w_\textrm{typ}} du\,g_\textrm{typ}(\textbf{k},u)\right) ^{-1}-\nu (\textbf{k})\;. \end{aligned}$$

An explicit expression for $\lambda _\textrm{typ}(\textbf{k},z)$ in terms of $\sigma _\textrm{typ}$ can thus be derived, reading

A23 $$\begin{aligned} \lambda _\textrm{typ}(\textbf{k},z)= &   \frac{1}{\nu (\textbf{k})-z\sigma _\textrm{typ}(z)+\int _{0}^z du\,\sigma _\textrm{typ}(u)}\nonumber \\= &   \frac{1}{\nu (\textbf{k})-\int _{0}^z du\,z\,\sigma _\textrm{typ}'(u)}=\frac{1}{\nu (\textbf{k})+\int _{Q_\textrm{typ}(z)}^{Q_\textrm{typ}} du\,z_\textrm{typ}(q)\,f''(q)}\;, \end{aligned}$$

where we have used that $\sigma _\textrm{typ}(z)=f'(Q_\textrm{typ}(z))$ and defined $Q_\textrm{typ}\equiv Q_\textrm{typ}(0)$.

Taking a derivative with respect to $z_0$ of Eq. (A21) yields

A24 $$\begin{aligned} \int _\textbf{k}\partial _{z}g_\textrm{typ}(\textbf{k},z)f''\left( \int _\textbf{k}g_\textrm{typ}(\textbf{k},z)\right) -\frac{\partial _{z} g_\textrm{typ}(\textbf{k},z)}{\lambda _\textrm{typ}(\textbf{k},z)^2}=0\;. \end{aligned}$$

After multiplication by $\lambda _\textrm{typ}(\textbf{k},z)^2$ and integration over $\textbf{k}$, one obtains the identity

A25 $$\begin{aligned} \int _\textbf{k}\lambda _\textrm{typ}(\textbf{k},z)^2=\frac{1}{\displaystyle f''\left( \int _\textbf{k}g_\textrm{typ}(\textbf{k},z)\right) }\;,\;\;z\in [0,w_\textrm{typ}]\;. \end{aligned}$$

Introducing $Q_\textrm{typ}(z)=\int _\textbf{k}g_\textrm{typ}(\textbf{k},z)$, one obtains the identity

A26 $$\begin{aligned} I_2\left( \mu -zf'(Q_\textrm{typ}(z))+\int _{0}^z du\,f'(Q_\textrm{typ}(u))\right) f''(Q_\textrm{typ}(z))=1\;,\;\;z\in [0,w_\textrm{typ}]\;, \end{aligned}$$

where we have used that

A27 $$\begin{aligned} \int _\textbf{k}\lambda _\textrm{typ}(\textbf{k},z)^p&=\int _\textbf{k}\frac{1}{\left( \nu (\textbf{k})-z\sigma _\textrm{typ}(z)+\int _{0}^z du\,\sigma _\textrm{typ}(u)\right) ^p}\nonumber \\&=I_p\left( \mu -zf'(Q_\textrm{typ}(z))+\int _{0}^z du\,f'(Q_\textrm{typ}(u))\right) \nonumber \\&=I_p\left( \mu +\int _{Q_\textrm{typ}(z)}^{Q_\textrm{typ}} dq\,z_\textrm{typ}(q)\,f''(q)\right) \;. \end{aligned}$$

The function $z_\textrm{typ}(q)$ is the inverse function of $Q_\textrm{typ}(z)$ and $Q_\textrm{typ}\equiv Q_\textrm{typ}(0)$. If the function $f''$ is monotonic and thus invertible, one may thus obtain the identity

A28 $$\begin{aligned} Q_\textrm{typ}(z)&=\int _\textbf{k}g_\textrm{typ}(\textbf{k},z)={f''}^{-1}\left( \frac{1}{\displaystyle \int _\textbf{k}\lambda _\textrm{typ}(\textbf{k},z)^2}\right) \nonumber \\&={f''}^{-1}\left( \frac{1}{\displaystyle \int _\textbf{k}\frac{1}{\left( \nu (\textbf{k})+\int _{Q_\textrm{typ}(z)}^{Q_\textrm{typ}} dq\,z_\textrm{typ}(q)\,f''(q)\right) ^2}}\right) \;. \end{aligned}$$

In particular, one obtains that

A29 $$\begin{aligned} Q_\textrm{typ}\equiv Q_\textrm{typ}(0)={f''}^{-1}\left( \frac{1}{\displaystyle \int _\textbf{k}\frac{1}{\nu (\textbf{k})^2}}\right) ={f''}^{-1}\left( \frac{1}{I_2(\mu )}\right) \;. \end{aligned}$$

Finally, the function $\sigma _\textrm{typ}(z)$ is shown to satisfy the self-consistent equation

A30 $$\begin{aligned}  &   \sigma _\textrm{typ}(z)=f'\left( Q_\textrm{typ}(z)\right) =f'\left[ {f''}^{-1}\left( \frac{1}{\int _\textbf{k}\lambda _\textrm{typ}(\textbf{k},z)^2}\right) \right] \nonumber \\= &   f'\left[ {f''}^{-1}\left( \frac{1}{\displaystyle \int _\textbf{k}\frac{1}{\left( \nu (\textbf{k})-z\sigma _\textrm{typ}(z)+\int _{0}^z du\,\sigma _\textrm{typ}(u)\right) ^2}}\right) \right] \;. \end{aligned}$$

Taking an additional derivative, one obtains the expression

A31 $$\begin{aligned} z= &   -\frac{\left( \int _\textbf{k}\lambda _\textrm{typ}(\textbf{k},z)^2\right) ^{3}}{2\int _\textbf{k}\lambda _\textrm{typ}(\textbf{k},z)^3}f^{(3)}\left( {f''}^{-1}\left( \frac{1}{\int _\textbf{k}\lambda _\textrm{typ}(\textbf{k},z)^2}\right) \right) \nonumber \\= &   -\frac{f^{(3)}\left( Q_\textrm{typ}(z)\right) }{2f''\left( Q_\textrm{typ}(z)\right) ^3 I_3\left( \mu -zf'(Q_\textrm{typ}(z))+\int _{0}^z du\,f'(Q_\textrm{typ}(u))\right) }\;. \end{aligned}$$

Taking an additional derivative with respect to *z*, one obtains

A32 $$\begin{aligned}&-\frac{1}{z\sigma _\textrm{typ}'(z)}=&\frac{3}{2}\left( 2-\frac{\int _\textbf{k}\lambda _\textrm{typ}(\textbf{k},z)^4\int _\textbf{k}\lambda _\textrm{typ}(\textbf{k},z)^2}{\left( \int _\textbf{k}\lambda _\textrm{typ}(\textbf{k},z)^3\right) ^2}\right) \frac{f^{(3)}\left( Q_\textrm{typ}(z)\right) }{f''\left( Q_\textrm{typ}(z)\right) ^2}\nonumber \\&\quad -\frac{f^{(4)}\left( Q_\textrm{typ}(z)\right) }{f^{(3)}\left( Q_\textrm{typ}(z)\right) f''\left( Q_\textrm{typ}(z)\right) }\;. \end{aligned}$$

Finally, let us use the identity $\partial _z \lambda (\textbf{k},z)=z\partial _z g(\textbf{k},z)$ to express

A33 $$\begin{aligned} g(\textbf{k},z)&=\int _{w_M}^{z}dz_0\frac{Q_\textrm{typ}'(z_0)f''[Q_\textrm{typ}(z_0)]}{\left( \nu (\textbf{k})+\int _{Q_\textrm{typ}(z_0)}^{Q_\textrm{typ}} du\,z_\textrm{typ}(q)\,f''(q)\right) ^2}\nonumber \\&=\int _{0}^{Q_\textrm{typ}(z)}\frac{f''(q)dq}{\left( \nu (\textbf{k})+\int _{q}^{Q_\textrm{typ}} dr\,z_\textrm{typ}(r)\,f''(r)\right) ^2}\;. \end{aligned}$$

To simplify the expression of the typical energy, we re-express

A34 $$\begin{aligned}&\mathcal{S}_\textrm{typ}+\frac{f'(0)}{2}\int _\textbf{k}\frac{1}{\nu (\textbf{k})}\nonumber \\&=\frac{1}{2}\int _\textbf{k}\nu (\textbf{k})\left[ g(\textbf{k},0)+G_0(\textbf{k})\right] +\frac{1}{2}\left[ f'(0)\int _\textbf{k}v(\textbf{k})-w_M\,f\left( 0\right) +\int _{0}^{w_M}dz\,f\left( Q(z)\right) \right] \nonumber \\&+\frac{1}{2}\int _\textbf{k}\left[ \frac{1}{w_M}\ln \frac{v(\textbf{k})}{\lambda (\textbf{k},0)}-\frac{G_0(\textbf{k})}{\lambda (\textbf{k},0)}+\int _{0}^{w_M}\frac{dz}{z^2}\ln \frac{\lambda (\textbf{k},z)}{\lambda (\textbf{k},0)}\right] \;,\nonumber \\ =&\frac{f'(0)}{2}\int _\textbf{k}\frac{1}{\nu (\textbf{k})+\int _{0}^{Q_\textrm{typ}} dq\,z_\textrm{typ}(q)\,f''(q)}+\frac{1}{2}\int _0^{w_M} dz\,z\,Q'(z)\,f'(Q(z))\nonumber \\&+\frac{1}{2}\int _\textbf{k}\int _{0}^{w_M}\partial _z g(\textbf{k},z)\left[ \frac{1}{\lambda (\textbf{k},z)}-\nu (\textbf{k})\right] \nonumber \\ =&\frac{f'(0)}{2}\int _\textbf{k}\frac{1}{\nu (\textbf{k})+\int _{0}^{Q_\textrm{typ}} dq\,z_\textrm{typ}(q)\,f''(q)}\nonumber \\&\quad +\frac{1}{2}\int _0^{w_M} dz\,Q'(z)\,\left[ z f'(Q(z))+\int _{Q(z)}^{Q_\textrm{typ}}dq\,z_\textrm{typ}(q)\,f''(q)\right] \nonumber \\ =&\frac{f'(0)}{2}\int _\textbf{k}\frac{1}{\nu (\textbf{k})+\int _{0}^{Q_\textrm{typ}} dq\,z_\textrm{typ}(q)\,f''(q)} \nonumber \\&\quad -\frac{1}{2}\int _0^{Q_\textrm{typ}} dq\,\left[ z_\textrm{typ}(q) f'(q)+\int _{q}^{Q_\textrm{typ}}dr\,z_\textrm{typ}(r)\,f''(r)\right] \end{aligned}$$

In the FRSB phase, the typical energy can thus be expressed as

A35 $$\begin{aligned} e_{L,\mathrm FRSB}(\mu )=-\frac{1}{2}\int _0^{Q_\textrm{typ}}z_\textrm{typ}(q)\left[ q f''(q)-f'(q)\right] +\frac{f'(0)}{2}\int _\textbf{k}\frac{1}{\nu (\textbf{k})+\int _0^{Q_\textrm{typ}}z_\textrm{typ}(q)f''(q)dq}\;. \end{aligned}$$

## Complexity

Let us compute the density of minima of the Hamiltonian at fixed energy density *e*, which reads

B1 $$\begin{aligned} \rho _{\min }(e)=&\int \mathcal{D}  \textbf{u}(\textbf{x})\,\left\langle \delta ^N\left( \frac{\delta \mathcal{H}[\textbf{u}]}{\delta \textbf{u}(\textbf{x})}\right) \det \mathcal{K}[\textbf{u}(\textbf{x})]\Theta (\mathcal{K}[\textbf{u}(\textbf{x})])\delta \left( NL^d e-\mathcal{H}[\textbf{u}]\right) \right\rangle \end{aligned}$$

B2 $$\begin{aligned} =&\sum _{\alpha :\textrm{minima}}\left\langle \delta \left( \frac{\mathcal{H}[\textbf{u}_{\alpha }]}{NL^d}-e\right) \right\rangle \ge P_{\min }(e)=\left\langle \delta \left( e_{\min }-e\right) \right\rangle \;, \end{aligned}$$

where $\mathcal{K}_{i \textbf{x},j \textbf{y}}=\frac{\delta ^2 \mathcal{H}[\textbf{u}]}{\delta u_i(\textbf{x})\delta u_j(\textbf{y})}$. In fact, one can compute exactly the double-sided Laplace transform of this quantity, i.e.

B3 $$\begin{aligned} \tilde{\rho }_{\min }(s)&=NL^d\int _{-\infty }^{\infty }\,de\,\rho _{\min }(e)\,e^{-NL^d s e} \nonumber \\&=\int \mathcal{D}  \textbf{u}(\textbf{x})\,\left\langle \delta ^N\left( \frac{\delta \mathcal{H}[\textbf{u}]}{\delta \textbf{u}(\textbf{x})}\right) \det \mathcal{K}[\textbf{u}(\textbf{x})]\Theta (\mathcal{K}[\textbf{u}(\textbf{x})])e^{-s\mathcal{H}[\textbf{u}]} \right\rangle \end{aligned}$$

B4 $$\begin{aligned}&=\sum _{\alpha :\textrm{minima}}\left\langle e^{-s \mathcal{H}[u_{\alpha }]} \right\rangle \ge \left\langle e^{-s N L^d e_{\min }} \right\rangle \;. \end{aligned}$$

Note that for $s=0$, $\tilde{\rho }_{\min }(s=0)=\left\langle \mathcal{N}_{\min }(\mu ) \right\rangle$ is simply the average number of minima. To perform this computation, one should compute the statistics of the Hessian conditioned on (i) a vanishing gradient and (ii) a fixed value of the disordered potential. One can simply realise using the translationally invariant form of the covariance, similarly to the computation of $\left\langle \mathcal{N}_{\min }(\mu ) \right\rangle$ [18], that the gradient $\frac{\delta \mathcal{H}[\textbf{u}]}{\delta \textbf{u}(\textbf{x})}$ is uncorrelated with both the Hessian $\mathcal{K}[\textbf{u}(\textbf{x})]$ and the disordered potential $V(\textbf{u},\textbf{x})$. One can compute explicitly the conditional expectation of the Hessian as

B5 $$\begin{aligned} \left\langle \mathcal{K}_{i\textbf{x},j\textbf{y}}|V(\textbf{u},\textbf{x})=Nv(\textbf{x}) \right\rangle =-t\Delta _{\textbf{x},\textbf{y}}\delta _{ij}+\left( \mu +\frac{f'(0)}{f(0)}v(\textbf{x})\right) \delta _{ij}\delta _{\textbf{x}  \textbf{y}} \end{aligned}$$

while its conditional covariance reads

B6 $$\begin{aligned}  &   \textrm{Cov}\left[ \frac{\partial ^2}{\partial u_i\partial u_j}V(\textbf{u},\textbf{x}),\frac{\partial ^2}{\partial u_k\partial u_l}V(\textbf{u},\textbf{x})|V(\textbf{u},\textbf{x})=Nv(\textbf{x})\right] =\frac{f''(0)}{N}(\delta _{ik}\delta _{jl}+\delta _{il}\delta _{jk}) \nonumber \\  &   +\frac{1}{N}\left( f''(0)-\frac{f'(0)^2}{f(0)}\right) \delta _{ij}\delta _{kl}\;. \end{aligned}$$

Computing explicitly the Gaussian integration over the field $\textbf{u}(\textbf{x})$, one obtains

B7 $$\begin{aligned}&\int \mathcal{D}  \textbf{u}(\textbf{x})\,\left\langle \delta ^N\left( \frac{\delta \mathcal{H}[\textbf{u}]}{\delta \textbf{u}(\textbf{x})}\right) e^{-s\mathcal{H}[\textbf{u}]} \right\rangle \end{aligned}$$

B8 $$\begin{aligned}&=\left( -2\pi f'(0)\right) ^{-\frac{NL^d}{2}}\int \mathcal{D}  \textbf{u}(\textbf{x})\,e^{\frac{1}{2f'(0)}\sum _{\textbf{x},\textbf{y}}  \textbf{u}(\textbf{x})\left[ (\mu \mathbb {I}-t\Delta )((\mu -s f'(0))\mathbb {I}-t\Delta )\right] _{\textbf{x}  \textbf{y}}  \textbf{u}(\textbf{y})}\left\langle e^{-s\sum _\textbf{x}V(\textbf{u},\textbf{x})} \right\rangle \end{aligned}$$

B9 $$\begin{aligned}&=\prod _\textbf{x}\int _{-\infty }^{\infty }\frac{dv(\textbf{x})}{\sqrt{2\pi f(0)/N}}e^{-N\left[ s v(\textbf{x})+\frac{v(\textbf{x})^2}{2f(0)}\right] }\det \left( K(\mu )K(\mu -s f'(0))\right) ^{-1/2}\;, \end{aligned}$$

where $K_{i\textbf{x},j\textbf{y}}(\mu )=\delta _{ij}\left( \mu \delta _{\textbf{x}  \textbf{y}}-t\Delta _{\textbf{x}  \textbf{y}}\right)$. The function $\tilde{\rho }_{\min }(s)$ can be computed exactly as

B10 $$\begin{aligned} \tilde{\rho }_{\min }(s)=&\prod _\textbf{x}\int _{-\infty }^{\infty }\frac{N dv(\textbf{x})d\xi (\textbf{x})}{2\pi \sqrt{f''(0)f(0)-f'(0)^2}}e^{-N\left[ s v(\textbf{x})+\frac{v(\textbf{x})^2}{2f(0)}+\frac{\left( \xi (\textbf{x})-\frac{f'(0)}{f(0)}v(\textbf{x})\right) ^2}{2\left( f''(0)-\frac{f'(0)^2}{f(0)}\right) }-s e_\textrm{RS}\right] } \end{aligned}$$

B11 $$\begin{aligned}&\times \frac{\det (K(\mu )+X+\sqrt{f''(0)}H)\Theta \left( K(\mu )+X+\sqrt{f''(0)}H\right) }{\det \left( K(\mu )^{1/2}K(\mu -s f'(0))^{1/2}\right) }\end{aligned}$$

B12 $$\begin{aligned}&=\prod _\textbf{x}\int _{-\infty }^{\infty }\sqrt{\frac{N}{2\pi f''(0)}}d\xi (\textbf{x})e^{-N\left[ \frac{\left( \xi (\textbf{x})+s f'(0)\right) ^2}{2 f''(0)}-\frac{s^2}{2}f(0)\right] } \nonumber \\&\frac{\det (K(\mu )+X+\sqrt{f''(0)}H)\Theta \left( K(\mu )+X+\sqrt{f''(0)}H\right) }{\det \left( K(\mu )^{1/2}K(\mu -s f'(0))^{1/2}\right) }\end{aligned}$$

B13 $$\begin{aligned}&=e^{NL^d\frac{s^2 f(0)}{2}}\left( \frac{\det (K(\mu -s f'(0))) }{\det (K(\mu ))}\right) ^{1/2}\left\langle \mathcal{N}_{\min }(\mu -s f'(0)) \right\rangle \;, \end{aligned}$$

where $X_{i\textbf{x},j\textbf{y}}=\delta _{ij}\delta _{\textbf{x}  \textbf{y}}\xi (\textbf{x})$, $H_{i\textbf{x},j\textbf{y}}=\delta _{\textbf{x}  \textbf{y}}h_{ij}(\textbf{x})$ where the $h(\textbf{x})$’s are iid standard GOE matrices. Note that the computation above is exact. One can then use the results from [18] to express the annealed complexity of minima as

B14 $$\begin{aligned}  &   \Sigma _{\min }(\mu )=\lim _{NL^d\rightarrow \infty }\frac{1}{NL^d}\ln \left\langle \mathcal{N}_{\min }(\mu ) \right\rangle =\nonumber \\  &   {\left\{ \begin{array}{ll} \displaystyle 0\;,\;\;\mu \ge \mu _{c}\;, \\ &  \\ \displaystyle -\frac{(\mu -\mu _{c})^2}{2}+\int _{\mu }^{\mu _{c}}dx\int _\textbf{k}\left[ \frac{1}{\nu (\textbf{k})-\mu +x}-\frac{1}{\nu (\textbf{k})-\mu +\mu _{c}}\right] \;,\;\;\mu \le \mu _{c}\;, \end{array}\right. } \end{aligned}$$

where $\mu _{c}$ satisfies $f''(0)I_2(\mu _c)=1$. Thus, as a function of *s*,

B15 $$\begin{aligned}&\Sigma _{\min }(\mu -s f'(0))=\lim _{NL^d\rightarrow \infty }\frac{1}{NL^d}\ln \left\langle \mathcal{N}_{\min }(\mu -s f'(0)) \right\rangle \end{aligned}$$

B16 $$\begin{aligned}&\quad ={\left\{ \begin{array}{ll} \displaystyle 0\;,\;\;s\ge s_\textrm{AT}\;,\\ & \\ \displaystyle -\frac{f'(0)^2(s-s_\textrm{AT})^2}{2}+\int _\textbf{k}\left[ \ln \frac{\nu (\textbf{k})-s f'(0)}{\nu (\textbf{k})-s_\textrm{AT} f'(0)}+\frac{(s-s_\textrm{AT})f'(0)}{\nu (\textbf{k})-s_\textrm{AT}f'(0)}\right] \;,\;\;s\le s_\textrm{AT}\;, \end{array}\right. } \end{aligned}$$

where the parameter $s_\textrm{AT}$ satisfies

B17 $$\begin{aligned} s_\textrm{AT}=\frac{\mu -\mu _c}{f'(0)}\;. \end{aligned}$$

For general value of *s*, one obtains that

B18 $$\begin{aligned} \tilde{\Sigma }_{\min }(s,\mu )=\lim _{NL^d\rightarrow \infty }\frac{1}{NL^d}\ln \tilde{\rho }_{\min }(s)&=\frac{s^2 f(0)}{2}+\frac{1}{2}\int _\textbf{k}\ln \left( 1-\frac{s f'(0)}{\nu (\textbf{k})}\right) +\Sigma _{\min }(\mu -s f'(0))\nonumber \\&\ge \lim _{NL^d\rightarrow \infty }\frac{1}{NL^d}\ln \left\langle e^{-N L^d s e_{\min }} \right\rangle =\varphi _L(s,\mu )\;. \end{aligned}$$

In the regime $s>s_\textrm{AT}$ where $\Sigma _{\min }(\mu -s f'(0))=0$ one obtains in particular that

B19 $$\begin{aligned} \tilde{\Sigma }_{\min }(s,\mu )&=\frac{s^2 f(0)}{2}+\frac{1}{2}\int _\textbf{k}\ln \left( 1-\frac{s f'(0)}{\nu (\textbf{k})}\right) =\varphi _{L,\mathrm RS}(s,\mu )\;. \end{aligned}$$

Thus, if the transition is continuous, i.e. it occurs at $s=s_\textrm{AT}$, the bound in Eq. (B18) is tight for any $s>s_\textrm{AT}$. If the transition is is a discontinuous transition, i.e. the transition occurs at $s_\textrm{dis}>s_\textrm{AT}$, this bound is still tight for any $s>s_\textrm{dis}$.

By Legendre transform, the expression for the annealed complexity of minima at fixed centred energy reads

B20 $$\begin{aligned} \Sigma _{\min }(e,\mu )=\min _{s\in \mathbb {R}}\left[ s e+\tilde{\Sigma }_{\min }(s,\mu )\right] \ge -\Lambda _L(e,\mu )\;, \end{aligned}$$

where the bound is a simple consequence of Eq. (B2). One recovers similarly that the bound is tight in the regime of validity of the RS ansatz. If the transition is continuous, the bound is tight for any $e\le e_\textrm{AT}$ while if the transition is discontinuous, it is tight only for $e\le e_\textrm{dis}<e_\textrm{AT}$.

## Some Integrals

In the expressions below, we use the following integrals:

C1 $$\begin{aligned}&\frac{S_{d-1}}{(2\pi )^d} L^{d- 2}\int _{2\pi /L}^{\infty }dk\,\frac{k^{d-1}}{\mu +k^{2}}=\mathcal{D}_{1,d}(\mu L^2)\end{aligned}$$

C2 $$\begin{aligned}&\mathcal{D}_{1,d}(x)=\frac{2\pi ^{d/2}}{2(2\pi )^2\Gamma (d/2)}\left( -\frac{x}{4\pi ^2}\right) ^{\frac{d-2}{2}}B\left( -\frac{x}{(4\pi )^2};\frac{2-d}{2},0\right) \;,\end{aligned}$$

C3 $$\begin{aligned}&\frac{S_{d-1}}{(2\pi )^d} L^{d- 4}\int _{2\pi /L}^{\infty }dk\,\frac{k^{d-1}}{(\mu +k^{2})^2}=\mathcal{D}_{2,d}(\mu L^2)\end{aligned}$$

C4 $$\begin{aligned}&\mathcal{D}_{2,d}(x)=\frac{2\pi ^{d/2}}{(2\pi )^4\Gamma (d/2)}\left[ \frac{1}{2(1+\frac{x}{4\pi ^2})}+\frac{d-2}{4}\left( -\frac{x}{4\pi ^2}\right) ^{\frac{d-4}{2}}B\left( -\frac{x}{(4\pi )^2};\frac{4-d}{2},0\right) \right] \;. \end{aligned}$$
